# Anoxygenic phototroph of the Chloroflexota uses a type I reaction centre

**DOI:** 10.1038/s41586-024-07180-y

**Published:** 2024-03-13

**Authors:** J. M. Tsuji, N. A. Shaw, S. Nagashima, J. J. Venkiteswaran, S. L. Schiff, T. Watanabe, M. Fukui, S. Hanada, M. Tank, J. D. Neufeld

**Affiliations:** 1 University of Waterloo, Waterloo, Ontario Canada; 2 Institute of Low Temperature Science, Hokkaido University, Sapporo, Japan; 3 Japan Agency for Marine-Earth Science and Technology, Yokosuka, Japan; 4 Tokyo Metropolitan University, Tokyo, Japan; 5 Wilfrid Laurier University, Waterloo, Ontario Canada; 6 Leibniz Institute DSMZ-German Collection of Microorganisms and Cell Cultures GmbH, Braunschweig, Germany; 7 Present Address: Kanagawa University, Yokohama, Japan; 8 Present Address: Bioproduction Research Institute, National Institute of Advanced Industrial Science and Technology (AIST), Tsukuba, Japan

**Keywords:** Microbial ecology, Ecophysiology, Bacterial evolution, Photosystem I, Water microbiology

## Abstract

Scientific exploration of phototrophic bacteria over nearly 200 years has revealed large phylogenetic gaps between known phototrophic groups that limit understanding of how phototrophy evolved and diversified^1,2^. Here, through Boreal Shield lake water incubations, we cultivated an anoxygenic phototrophic bacterium from a previously unknown order within the Chloroflexota phylum that represents a highly novel transition form in the evolution of photosynthesis. Unlike all other known phototrophs, this bacterium uses a type I reaction centre (RCI) for light energy conversion yet belongs to the same bacterial phylum as organisms that use a type II reaction centre (RCII) for phototrophy. Using physiological, phylogenomic and environmental metatranscriptomic data, we demonstrate active RCI-utilizing metabolism by the strain alongside usage of chlorosomes^3^ and bacteriochlorophylls^4^ related to those of RCII-utilizing Chloroflexota members. Despite using different reaction centres, our phylogenomic data provide strong evidence that RCI-utilizing and RCII-utilizing Chloroflexia members inherited phototrophy from a most recent common phototrophic ancestor. The Chloroflexota phylum preserves an evolutionary record of the use of contrasting phototrophic modes among genetically related bacteria, giving new context for exploring the diversification of phototrophy on Earth.

## Abstract (web summary)

Cultivation of a new anoxygenic phototrophic bacterium from Boreal Shield lake water—representing a transition form in the evolution of photosynthesis—offers insights into how the major modes of phototrophy diversified.

## Main

Chlorophyll-based phototrophy sustains life on Earth through the conversion of light into biologically usable energy^5,6^. Diverse microorganisms affiliated with at least eight bacterial phyla, discovered over nearly 200 years of scientific exploration^7–15^, perform this key process. Although these bacteria share common phototrophic ancestry^1,16^, many steps in their diversification remain unclear. Substantial gaps in the evolutionary record of phototrophy, apparent through inconsistent topology of photosynthesis gene phylogenies^2,17^ and a lack of transition forms between anoxygenic and oxygenic phototrophs^16^, have hindered our ability to answer fundamental questions about the order and timing of phototrophic evolution^17,18^. Discovery of evolutionary intermediates between known radiations of phototrophic life can help to resolve how phototrophs gained their modern functional characteristics.

Anoxygenic phototrophs belonging to the Chloroflexota (formerly Chloroflexi) phylum were first cultivated nearly 50 years ago^11^ and have since been characterized from diverse aquatic ecosystems^19–22^, but the evolution of phototrophy in this group has remained unclear. All known phototrophic Chloroflexota members use a RCII for light energy conversion^7,23,24^, yet several phototrophs within the Chloroflexota also contain chlorosomes, which are bacteriochlorophyll *c*-containing protein–pigment complexes, involved in light harvesting^3,25^, that are otherwise associated with RCI^13^. Although structurally homologous, RCI and RCII are functionally distinct and are well separated in the modern tree of life^1,16,26^. With the exception of oxygenic phototrophs, the only known lineage where RCI and RCII are used in tandem for electron flow^16^, each major lineage of phototrophic life is associated with only one of these two reaction centre classes^7,27^, and no examples of gene exchange between RCI-utilizing and RCII-utilizing phototroph groups have been reported in nature. How RCI-associated genes came to be encoded by RCII-utilizing Chloroflexota members thus represents an enigma linked to fundamental knowledge gaps in how the major modes of phototrophy diversified^16–18^.

Here we report the cultivation of a highly novel phototrophic bacterium that is phylogenetically related to known RCII-utilizing Chloroflexota members but uses chlorosomes and RCI, not RCII, for conversion of light energy. Discovery of the novel bacterium clarifies how chlorosomes came to be used by modern Chloroflexota members and substantially revises our view of the diversity of phototrophy. In this work, we demonstrate active usage of RCI by the novel strain and discuss the implications of our findings for the evolution of photosynthesis.

## Enrichment cultivation

**Extended Data Fig. 1:**
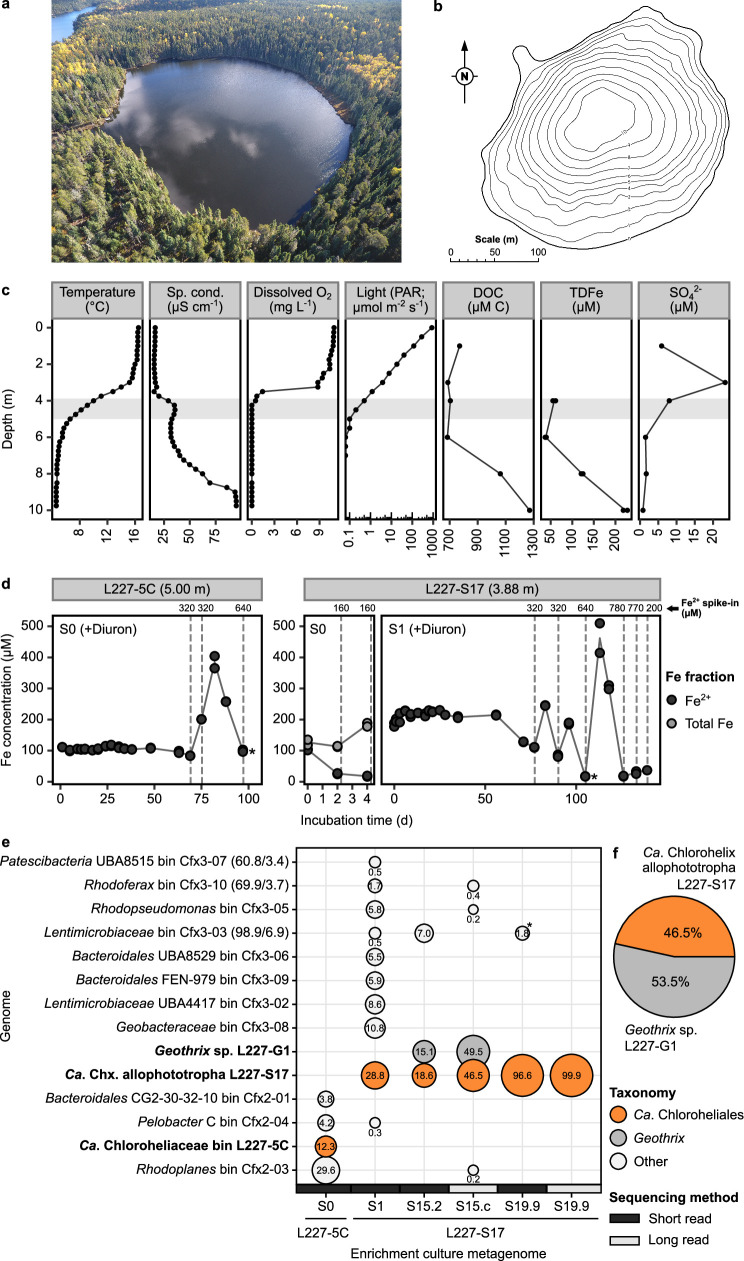
Sampling and enrichment cultivation of “*Ca*. Chloroheliales” members. **a**, Aerial photograph of Lake 227 (IISD-ELA, Canada), facing eastward. Reproduced with permission from IISD Experimental Lakes Area Inc. **b**, Bathymetry map of Lake 227. Contour lines of 1 m are shown. Adapted with permission from IISD Experimental Lakes Area Inc. **c**, Physicochemistry of the Lake 227 water column. A grey box marks the region of the water column from which water samples were collected for enrichment cultivation (top: 3.88 m; bottom: 5.00 m); these samples were collected during the same week as when samples for physicochemical parameters were collected. Biological replicate lake samples (n = 2) are shown as individual data points for TDFe measurements (lines show mean values). **d**, Fe concentrations of the L227-5C and L227-S17 enrichment cultures over time. The primary enrichment (S0) is shown for the L227-5C culture, whereas the primary enrichment (S0) and first subculture (S1) are shown for the L227-S17 culture. Asterisks mark the time points where samples were collected for metagenome sequencing. Technical replicates (n = 2) of Fe concentration samples are shown as individual data points (and lines as mean values) except for days 25, 28, and 139 of L227-S17 S1, where only single measurements were available. **e-f**, Microbial community composition the L227-5C and L227-S17 enrichment cultures over time. The bubble plot (**e**) shows the relative abundances of MAGs or closed genomes within enrichment culture metagenomes. Bubbles are shown where a genome recruited >0.1% of metagenome reads from a sample. The subculture generation of each enrichment is indicated by the number before the decimal place in its culture ID (e.g., S15.2 is a 15^th^ generation subculture). Estimated completeness and contamination (based on CheckM) are indicated in the taxon label for any MAG with <95% or >5% completeness or contamination, respectively. Nearly all (>95%) of the reads mapping to bin Cfx3-03 from the S19.9 short read metagenome (marked with an asterisk) were mapped to short contigs in Cfx3-03 that had >99% identity across 100% of their sequence to the strain L227-S17 genome. The pie chart (**f**) shows the microbial community composition of a representative subculture (S21.2c) of the stabilized L227-S17 enrichment culture. Relative abundances in the pie chart are based on 16S rRNA gene (V4 region) short read amplicon sequencing data with a detection sensitivity of 0.004%. Abbreviations: Sp. cond. Specific conductivity; PAR photosynthetically active radiation; DOC dissolved organic carbon; Fe iron; TDFe total dissolved iron.

With the original intention of cultivating anoxygenic phototrophs from the Chlorobiales order (phylum Bacteroidota), we sampled the anoxic water column of an iron-rich Boreal Shield lake (Extended Data Fig. 1a–c) and gradually amended lake water, incubated under light, with a previously published freshwater medium^28^ and ferrous chloride, using Diuron as an inhibitor of oxygenic phototrophs (Extended Data Fig. 1d). On the basis of 16S rRNA gene profiles, some of the incubated batch cultures developed high relative abundances of novel microbial populations that were only distantly associated with known Chloroflexota members (Supplementary Data 1). We used agar-containing medium to further enrich a novel strain, named L227-S17, from one batch culture that represented one of the sequence variants from earlier culture profiles (Extended Data Fig. 1e). In addition, via metagenome sequencing of a separate batch culture, we recovered a metagenome-assembled genome (MAG) corresponding to a second novel sequence variant, named strain L227-5C (Extended Data Fig. 1e and Supplementary Note 1).

After 19 subcultures over 4 years, strain L227-S17 was brought into a stable enrichment culture that included a putative iron-reducing bacterium, associated with the *Geothrix* genus^29^, named strain L227-G1 (Extended Data Fig. 1f and Supplementary Note 1). Under phototrophic growth conditions, only strains L227-S17 and L227-G1 were detectable in the culture, using 16S rRNA gene amplicon sequencing, to a detection limit of 0.004% (Extended Data Fig. 1f), allowing us to characterize the physiology of strain L227-S17 within a two-member culture system. On the basis of the RCI-utilizing phototrophic metabolism of L227-S17, we provisionally name the strain ‘*Candidatus* Chlorohelix allophototropha’ (a green spiral, phototrophic in a different way; the full etymology is provided in the ‘Species description’ section).

## Phototrophic physiology

**Fig. 1:**
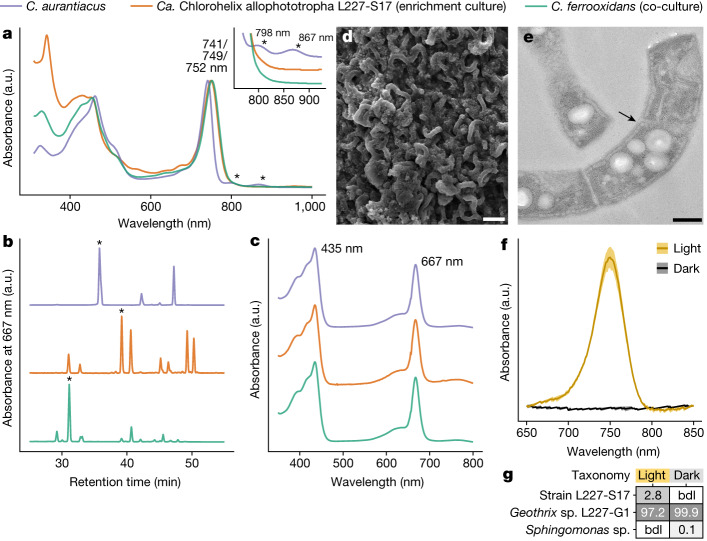
Phototrophic physiology of the L227-S17 culture. **a**, In vivo absorption spectrum of the L227-S17 culture compared with reference cultures. The inset shows the 760–925-nm region with spectra separated on the *y* axis. a.u., arbitrary units. **b**,**c**, Bacteriochlorophyll *c* species in the cultures. High-performance liquid chromatography (HPLC) profiles (**b**) and the in vitro absorption spectra associated with the largest HPLC peaks in the profiles (**c**) are shown. The largest HPLC peaks are marked with an asterisk in **b**. **d**, Scanning electron microscopy image of L227-S17 colony material from an early enrichment culture. **e**, Transmission electron microscopy image showing a longitudinal section of cells from an early L227-S17 enrichment culture. An example chlorosome-like structure is marked with an arrow. Scale bars, 3 μm (**d**) and 0.3 μm (**e**). Panels **d** and **e** are representative of imaging repeated more than five times on different regions of the same or related sample preparations. **f**,**g**, Light versus dark growth test of the L227-S17 culture amended with iron(II) and acetate. In vivo absorption spectra (**f**) and a heatmap of relative abundances (%) of 16S rRNA gene operational taxonomic units (**g**) are shown for the culture after two subcultures in the light or dark. Standard deviations (*n* = 3; biological replicate cultures) of the mean are shown as shaded areas in **f**. bdl, below detection limit. Source Data

**Extended Data Fig. 2:**
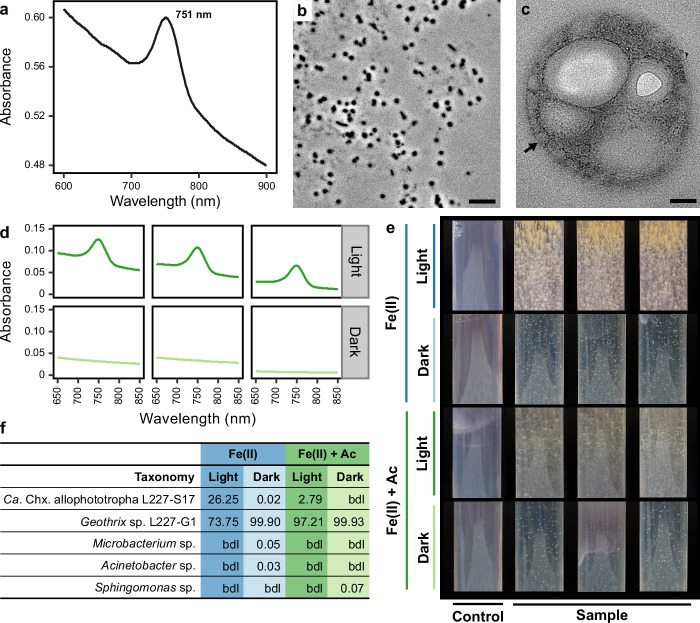
Supporting data for the phototrophic physiology of strain L227-S17. **a**, Absorption spectrum of suspended whole cells from the L227-S17 culture. **b**, Light microscopy of *Geothrix* sp. L227-G1 (dry mount) grown under fermentative conditions. **c**, Transmission electron microscopy image showing a cross section of filamentous cells in the L227-S17 culture. An example chlorosome-like structure is marked with an arrow. **d**, Non-normalized absorption spectra for cultures grown in iron(II) with acetate in the light vs. dark; normalized data are shown in Fig. 1f. **e**, Appearance of agar shake tubes containing L227-S17 cultures (with resazurin) grown in the light or dark. Tube diameter is 1.55 cm. **f**, Microbial community of L227-S17 cultures grown in the light or dark based on V1-V9 16S rRNA gene amplicon sequencing data. Data for iron(II) treatments with acetate are also shown in Fig. 1g but are shown here for comparison to iron(II) only (autotrophic) treatments. The scale bars in panels **b** and **c** represent 5 μm and 0.1 μm, respectively, and experiments for panels **b** and **c** were independently repeated >3 times (with different subcultures) and >5 times (on different zones of the same sample preparation), respectively, such that the images presented are representative microscopy results. Abbreviations: Fe(II) iron(II); Ac acetate.

We compared the phototrophic properties of the L227-S17 enrichment culture to properties of known bacterial phototrophs (Fig. 1 and Extended Data Fig. 2). The in vivo absorption spectrum of the culture included a strong absorbance peak at 749 nm, which is characteristic of chlorosome-containing phototrophic bacteria^13^ (Fig. 1a and Extended Data Fig. 2a; see Extended Data Table 1 for microbial community data associated with spectroscopy and microscopy analyses). Using high-performance liquid chromatography, we confirmed that the L227-S17 culture contained multiple bacteriochlorophyll *c* species that had absorbance peaks at 435 nm and 667 nm (ref. ^13^) (Fig. 1b,c), as well as bacteriochlorophyll *a* that can serve as a core reaction centre pigment^30^ (Supplementary Fig. 1). Large spiralling filaments composed of cells 0.5–0.6 µm wide and 2–10 µm long were visible in the culture (Fig. 1d,e) and were accompanied by smaller rod-shaped cells. The rod-shaped cells corresponded to the *Geothrix* L227-G1 strain based on enrichment of L227-G1 under dark conditions and subsequent microscopy (Extended Data Fig. 2b). Thus, we could establish that strain L227-S17 corresponded to the filamentous cells. The inner membranes of the filamentous cells contained electron-transparent and spherical structures, which matched the expected appearance of chlorosomes after fixation with osmium tetroxide^31^ (Fig. 1e and Extended Data Fig. 2c). Furthermore, strain L227-S17 and the 749-nm absorbance peak were consistently absent when the culture was incubated in the dark (Fig. 1f,g and Extended Data Fig. 2d). Although the culture was typically grown photoheterotrophically to stabilize growth, we could also grow the culture photoautotrophically and reproduce the loss of strain L227-S17 in the dark (Extended Data Fig. 2e,f). These data demonstrate that strain L227-S17 is the phototrophic and chlorosome-containing member of the enrichment culture.

Our spectroscopic data indicate that strain L227-S17 uses a related but novel phototrophic pathway compared with the RCII-utilizing *Chloroflexus aurantiacus*, a chlorosome-containing and phototrophic Chloroflexota member^11^. Although both strains shared the major chlorosome-associated peak at 740–750 nm in their in vivo absorption spectra, the spectrum of *C. aurantiacus* had additional absorbance peaks at 798 nm and 867 nm (Fig. 1a, inset). These peaks, as observed previously^32^, may represent the RCII-associated B808–866 complex and were absent in the RCI-associated *Chlorobium ferrooxidans* culture (Fig. 1a, inset). Suspended whole cells from the L227-S17 culture also lacked these peaks (Extended Data Fig. 2a). The L227-S17 and *C. aurantiacus* cultures both included multiple bacteriochlorophyll *c* species (Fig. 1b,c), but modifications to bacteriochlorophyll *c* species in the L227-S17 culture more closely matched those of the *C. ferrooxidans* culture (Fig. 1c). On the basis of these data, the L227-S17 culture had RCI-associated physiological properties, despite using chlorosomes and bacteriochlorophyll *c* species like previously known RCII-utilizing Chloroflexota members.

## Phototrophic gene pathway

**Extended Data Fig. 3:**
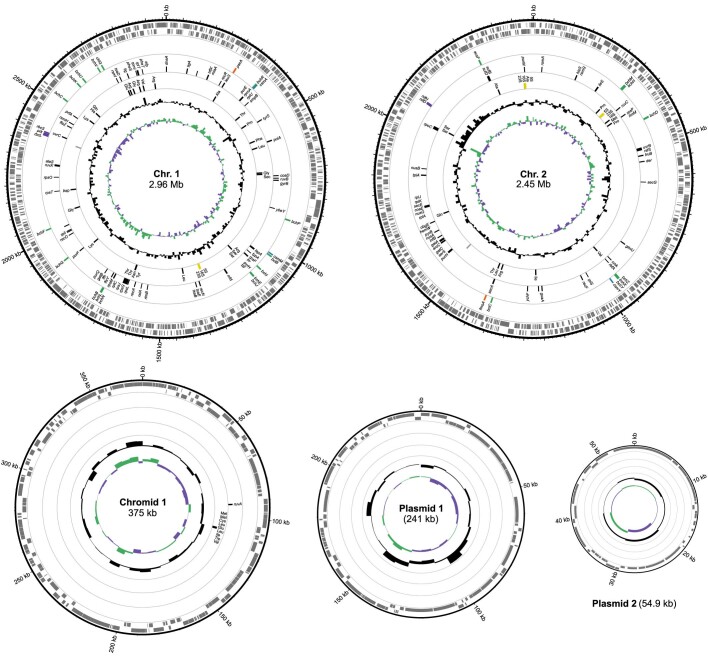
Complete genome of “*Ca*. Chx. allophototropha” strain L227-S17. A map of each of the five circular chromosomes in the genome is shown. In each map, data rings (from outside to inside) show the following information: forward-orientation genes; reverse-orientation genes; photosynthesis-related genes as shown in Extended Data Table 2 (following the colouring scheme of Fig. 3); single copy marker genes identified by CheckM; non-coding genes including rRNA genes (yellow), tRNA genes (black), and others (grey); GC content, shown as ±11.2% of the genome-wide average GC content of 46.8%; and GC skew, shown as ±23.5% of 0%. The GC content and GC skew are shown based on a sliding window of 10 kb. Chr., chromosome.

We identified the RCI-based metabolic potential of strain L227-S17 by analysing its complete genome sequence. We obtained a single, isolated colony of strain L227-S17 within an agar shake tube (subculture 19.9; Extended Data Fig. 1e) and confirmed that the colony was devoid of the *Geothrix* L227-G1 partner strain, using 16S rRNA gene amplicon sequencing (Extended Data Table 1), before proceeding with genomics. The closed L227-S17 genome consisted of two circular chromosomes (chromosome 1, 2.96 Mb; and chromosome 2, 2.45 Mb), one circular chromid (375 kb)^33^ and two circular plasmids (241 kb and 55 kb; Extended Data Fig. 3). Chromosomes 1 and 2 both encoded at least one copy of identical 16S rRNA genes (Extended Data Fig. 3) and encoded more than 98% non-overlapping single-copy marker genes. By similarly performing DNA sequencing from early enrichment cultures (subcultures 15.2 and 15.c, respectively; Extended Data Fig. 1e), we obtained a closed genome bin for the *Geothrix* L227-G1 partner strain that consisted of a single circular chromosome (3.73 Mb) and encoded no phototrophic marker genes.

**Fig. 2:**
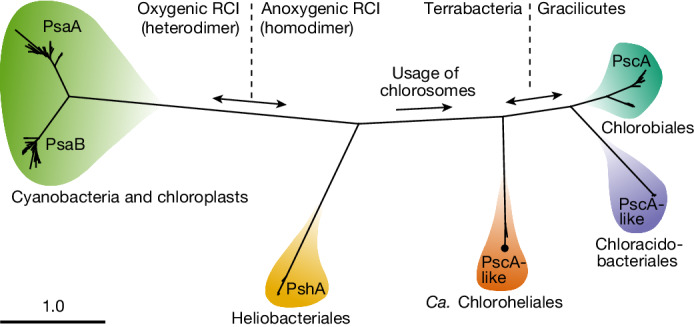
Maximum-likelihood phylogeny of RCI primary sequences. Chlorophototrophic lineages are summarized by order name (except Cyanobacteria and chloroplasts). The placement of strain L227-S17 is indicated by a black dot. The expected proportion of amino acid change is indicated by the scale bar. All internal nodes between shaded lineages had 100% bootstrap support.

**Extended Data Fig. 4:**
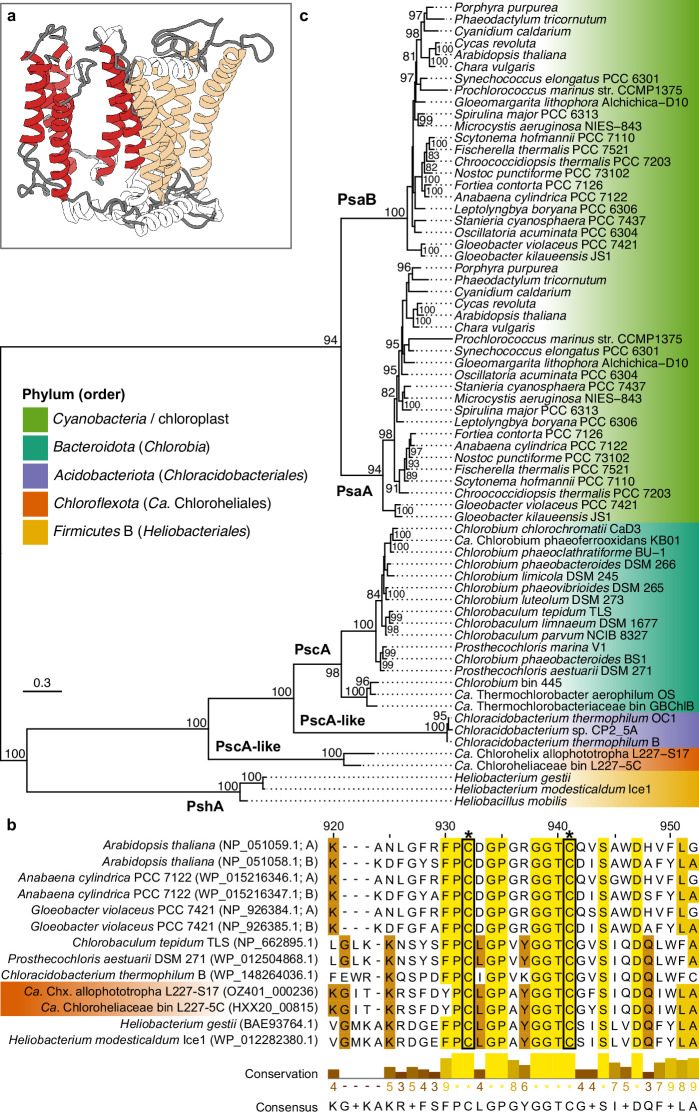
Additional information about the Type I reaction center of strain L227-S17. **a**, Predicted tertiary structure of the novel PscA-like primary sequence encoded by strain L227-S17 based on homology modelling. The six N-terminal and five C-terminal transmembrane helices expected for RCI are coloured in red and tan, respectively. **b**, Amino acid sequence alignment of selected RCI sequences. The alignment shows the [4Fe-4S] binding site that is conserved among known RCI-utilizing phototrophs. Cysteine residues thought to bind the [4Fe-4S] cluster are marked with a black outline and asterisk. **c**, Maximum likelihood phylogeny of oxygenic and anoxygenic Type I reaction center predicted protein sequences. A simplified depiction of the same phylogeny is shown in Fig. 2. The phylogeny is midpoint rooted, and bootstrap values of at least 80% are shown. The scale bar represents the expected proportion of amino acid change.

We found no evidence of the RCII-associated *pufLM* genes, used by all known phototrophic Chloroflexota members, in the strain L227-S17 genome. Instead, we identified a remote homologue of known RCI genes, analogous to *pscA*^34^, on chromosome 1 (Fig. 2 and Extended Data Fig. 4). The PscA-like primary sequence had only approximately 30% amino acid identity to closest-matching RCI sequences in RefSeq^35^ (as of January 2023), but the sequence included a conserved [4Fe–4S] cluster-binding site and was predicted to fold into 11 transmembrane helices as expected for a RCI protein, supporting its functional role^36^ (Extended Data Fig. 4a,b). On the basis of a maximum-likelihood amino acid sequence phylogeny (Fig. 2 and Extended Data Fig. 4c), the novel PscA-like predicted protein represents a distinct fifth clade of RCI protein, excluding the possibility of recent lateral gene transfer from other known phototrophic groups. We could replicate our findings of the novel *pscA*-like gene in the MAG of the uncultured L227-5C strain, adding further support that the novel RCI gene is associated with Chloroflexota (Extended Data Fig. 4c).

**Fig. 3:**
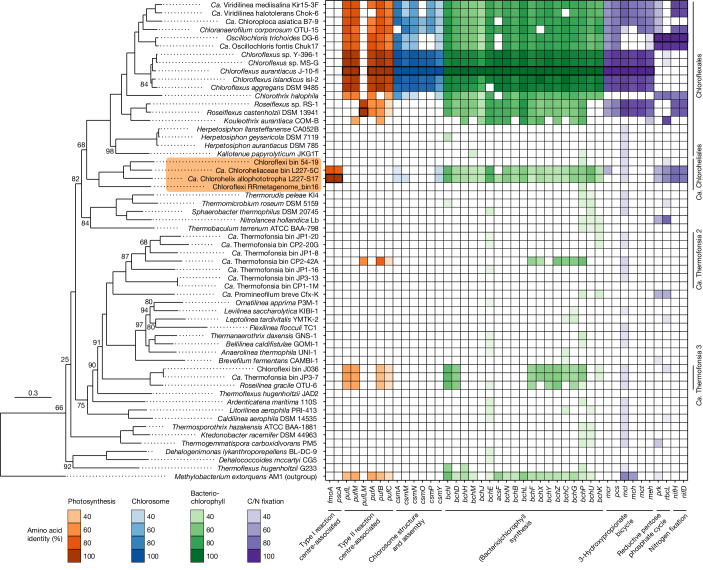
Genomic potential for phototrophy among Chloroflexota members. The maximum-likelihood phylogeny is based on a set of 74 concatenated core bacterial proteins. The scale bar indicates the expected proportion of amino acid change. Bootstrap values of 100% are omitted for readability; all other bootstrap values are shown. The ‘*Ca*. Chloroheliales’ clade is highlighted in orange. The heatmap shows the presence or absence of genes involved in photosynthesis or related processes based on bidirectional BLASTP. Heatmap tiles of query genes have bolded outlines. Source Data

**Extended Data Fig. 5:**
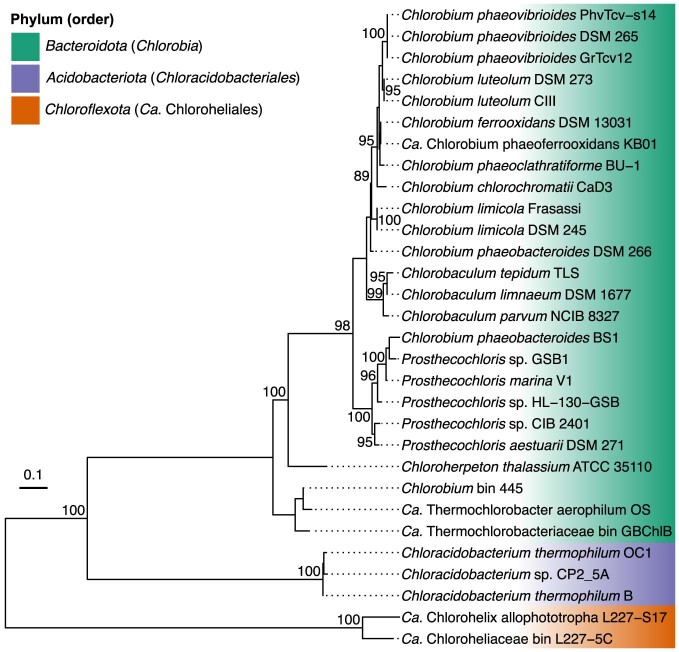
Maximum likelihood phylogeny of Fenna-Matthews-Olson (FMO) protein (FmoA) sequences. The phylogeny is midpoint rooted, and bootstrap values of at least 80% are shown. The scale bar represents the expected proportion of amino acid change.

Along with the *pscA*-like gene, we detected genes for a complete RCI-based phototrophic pathway in the strain L227-S17 genome (Fig. 3, Extended Data Table 2 and Supplementary Data 2). We detected a remote homologue of *fmoA*, which encodes the Fenna–Matthews–Olson (FMO) protein involved in energy transfer from chlorosomes to RCI^37^ (Extended Data Fig. 5), making the Chloroflexota the third known phylum to potentially use the FMO protein for phototrophy^13^. Matching physiological observations, we detected a homologue of the key chlorosome-associated gene *csmA* involved in chlorosome baseplate formation^3,4^. Chlorosome-associated proteins outside CsmA are poorly conserved across different species^3^, so although we could detect only two other homologues to chlorosome-associated proteins used by *C. aurantiacus*, strain L227-S17 may use additional novel proteins in its chlorosomes. In place of alternative complex III, which is encoded by previously known Chloroflexota members and is associated with photosynthetic electron transfer^38^, we found a cytochrome *b*_*6*_*f*-like gene cluster^39^ related to that of Heliobacteriales members (Supplementary Note 2). Most other genes involved in photosynthetic electron transfer are not conserved among RCI-utilizing phototrophic lineages, but we identified a possible homologue of the Chlorobiales-associated *pscB* gene^34^, whose gene product holds the terminal-bound electron acceptors of RCI, in the strain L227-S17 genome, along with ferredoxin gene homologues that could be involved in subsequent electron flow (Supplementary Note 2).

**Extended Data Fig. 6:**
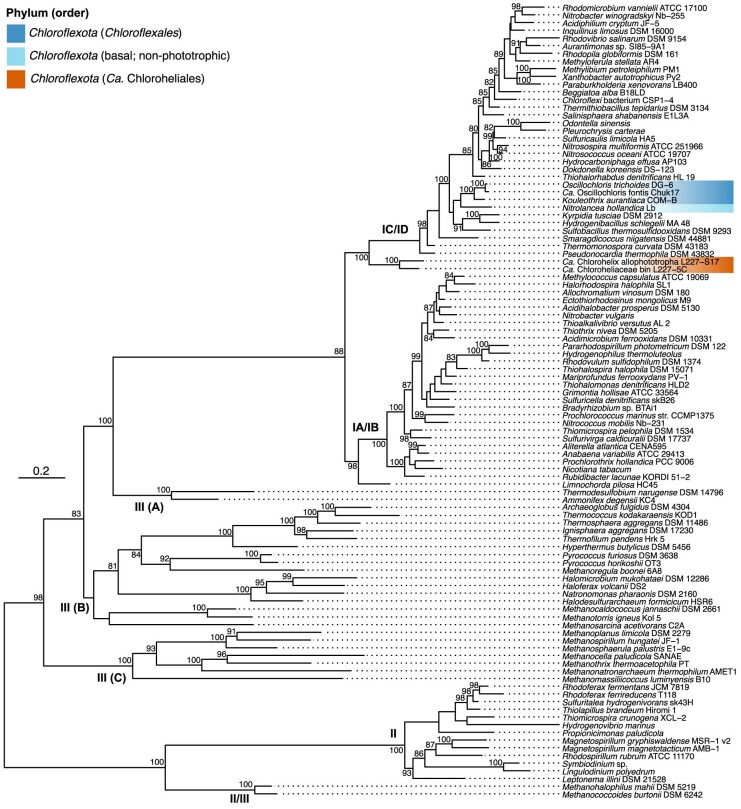
Maximum likelihood phylogeny of RuBisCO large subunit (RbcL) predicted protein sequences. Group I to III RbcL sequences are included. The phylogeny is midpoint rooted, and bootstrap values of at least 80% are shown. The scale bar represents the expected proportion of amino acid change.

Supporting spectroscopic data, we detected genes for the entire biosynthetic pathway of bacteriochlorophylls *a* and *c* from protoporphyrin IX in the strain L227-S17 genome^30^ (Extended Data Table 2). In addition, we detected a deeply branching *chlG*-like paralogue of *bchG* that may serve as chlorophyll *a* synthase, although we note that this gene placed phylogenetically sister to Heliobacteriales-associated bacteriochlorophyll *g* synthases (Supplementary Fig. 2). We identified genes unique to the reductive pentose phosphate (also known as Calvin–Benson–Bassham) cycle involved in carbon fixation, including a deep-branching class IC-ID *rbcL* gene^40^ representing the large subunit of RuBisCO (Extended Data Fig. 6). We did not detect genomic potential for the 3-hydroxypropionate bicycle, which is used for carbon fixation by some RCII-utilizing Chloroflexales members, aside from detection of malyl-CoA lyase (MCL), which is also encoded by Chloroflexota members incapable of this carbon fixation pathway^41^. At the whole-genome level, we observed no large photosynthetic gene clusters in the strain L227-S17 genome and saw no clear tendency for phototrophy-related genes to be encoded by chromosome 1 versus chromosome 2 (Extended Data Fig. 3). Together, genomic data demonstrate that ‘*Ca*. Chlorohelix allophototropha’ L227-S17 has metabolic potential for RCI-driven phototrophy using several highly novel genes compared with known phototrophs.

## Ecology in Boreal Shield lakes

**Fig. 4:**
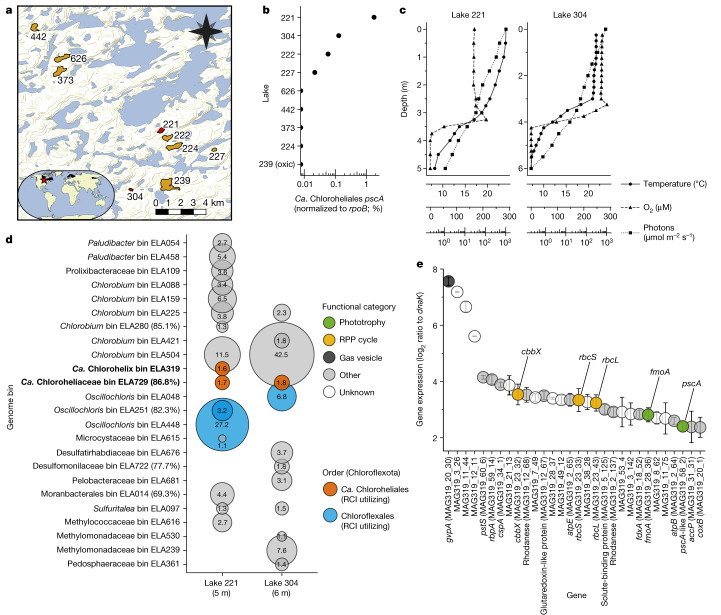
Distribution and activity of strain L227-S17 relatives in Boreal Shield lakes. **a**, Map of the nine sampled Boreal Shield lakes. The inset shows the location of the sampling site (red star) and the approximate range of Boreal Shield regions on Earth (black highlights). Basemap data are from refs. ^51–54^. **b**, Detection of strain L227-S17-like RCI genes based on unassembled Boreal Shield lake metagenomes. **c**, Physical profile data for lakes 221 and 304, sampled in July 2018. **d**, Bubble plot showing active microbial populations in the water columns of lakes 221 and 304 based on mapping of metatranscriptome data to MAGs. The bubble size reflects the mean relative expression (*n* = 3, metatranscriptomes from biological replicate filters; see Methods) of each MAG, and MAGs with greater than 1% relative expression are shown. Standard deviations were 7.0% of the mean on average; see Supplementary Data 5 for exact values. All MAGs had greater than 90% estimated completion unless indicated beside the MAG ID. RPP, reductive pentose phosphate. **e**, Gene expression of bin ELA319 in the Lake 221 (5 m) dataset. Mean normalized gene expression values are shown for the top 30 highest expressed protein-coding genes; the error bars show the standard deviation of metatranscriptomes derived from biological replicate filters (*n* = 3). Additional gene expression data are presented in Extended Data Fig. 7 and Supplementary Data 6. Source Data

We examined the ecology and environmental activity of relatives of strain L227-S17 in Boreal Shield lakes^42^ to verify their RCI-based metabolic lifestyle (Fig. 4). Selecting eight seasonally anoxic Boreal Shield lakes nearby (and including) Lake 227, we sampled the depth profile of water columns over 3 years for DNA and optionally RNA sequencing (Fig. 4a). All but one of the eight lakes developed ferruginous (that is, iron-rich and sulfate-poor) waters after the onset of anoxia (that is, all but Lake 626), and the lakes had measurable light penetration into their anoxic zones despite contrasting physicochemical properties, such as dissolved organic carbon and total dissolved iron concentrations (Extended Data Table 3 and Supplementary Data 3). We detected L227-S17-associated *pscA*-like genes in four of the eight seasonally anoxic lakes (that is, lakes 221, 304, 222 and 227) based on searching unassembled metagenome data, with *rpoB*-normalized abundances of up to 1.8% in Lake 221 (Fig. 4b). Metagenome assembly, genome binning and bin dereplication allowed us to recover two MAGs (out of a total of 756 MAGs) that were affiliated with the Chloroflexota phylum and encoded a L227-S17-like RCI gene homologue. We detected these two dereplicated MAGs in samples from the same four lakes at greater than 0.01% relative abundance and sometimes across multiple sampling years (Supplementary Data 4), demonstrating that RCI-associated Chloroflexota members can form robust populations that are widespread among Boreal Shield lakes in this region despite seasonal lake mixing.

**Extended Data Fig. 7:**
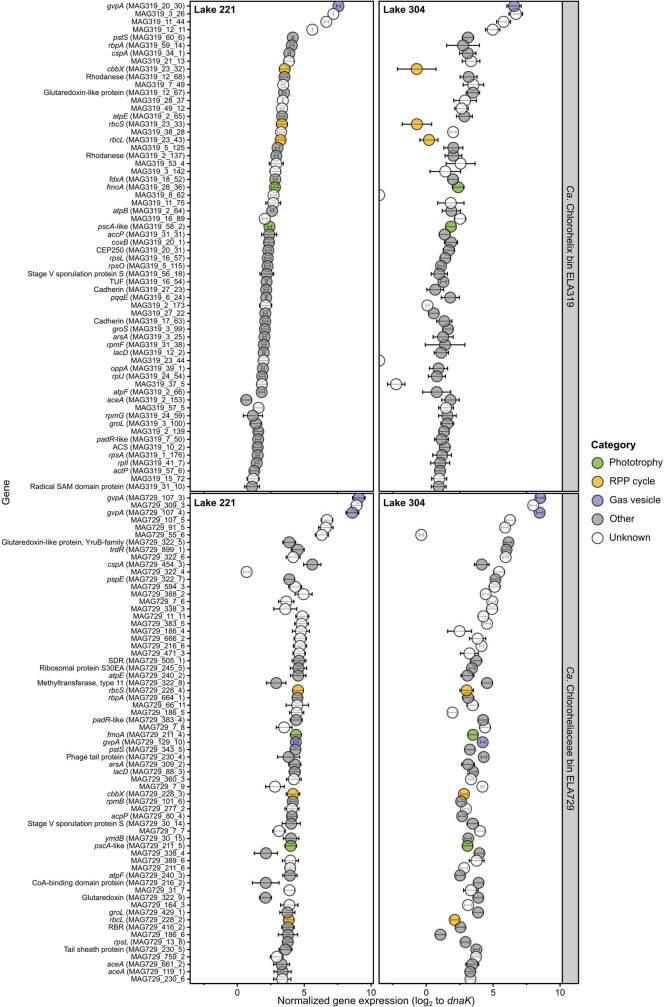
Highly expressed genes among “*Ca*. Chloroheliales”-associated genome bins within Lake 221 and 304 metatranscriptome data. The top 50 protein-coding genes, based on mean normalized gene expression, are shown for genome bins ELA319 and ELA729. Error bars represent the standard deviation of the log_2_ expression ratio (n = 3) for replicate metatranscriptomes derived from biological replicate filters. Genes potentially involved in phototrophy, carbon fixation, and buoyancy are highlighted. Normalized expression values for all genes, along with their predicted amino acid sequences and annotations, are included in Supplementary Data 6.

We probed the in situ gene expression of the two RCI-encoding Chloroflexota MAGs using metatranscriptomes associated with the illuminated and anoxic water columns of lakes 221 and 304. Light penetration into the anoxic zones of lakes 221 and 304 (Fig. 4c) was roughly an order of magnitude higher than Lake 227 (Extended Data Fig. 1c), where a surface cyanobacterial bloom blocks light penetration in the summer^42,43^. The two RCI-encoding Chloroflexota MAGs were highly active compared with other bacterial populations based on RNA data, recruiting as much as 1.8% of mappable metatranscriptome reads from the Lake 221 and Lake 304 samples (Fig. 4d and Supplementary Data 5). Both MAGs had upregulated expression of the *pscA*-like RCI gene, the *fmoA* gene and the *rbcLS* genes encoding RuBisCO (Fig. 4e; the full dataset is available in Extended Data Fig. 7 and Supplementary Data 6). The MAGs also had high expression levels of homologues of *gvpA*, involved in the formation of gas vesicles that may function in buoyancy regulation^44^. Moreover, the MAGs co-occurred with RCII-encoding Chloroflexota and RCI-encoding Chlorobiales-associated MAGs, which were among the highest RNA read-recruiting MAGs in the dataset (Fig. 4d). Our data thus demonstrate that RCI-based phototrophy is actively used by Chloroflexota members in natural environments. These RCI-utilizing Chloroflexota members potentially form part of a more complex phototrophic microbial consortium in Boreal Shield lake anoxic waters. Given that Boreal Shield lakes number in the millions globally^42^ and might commonly develop iron-rich and anoxic bottom waters, as observed in Fennoscandian lakes geographically distant from the lakes presented in this study^45^, phototrophic consortia including RCI-utilizing Chloroflexota members could be relevant to widespread northern ecosystems.

## Phylogenomic properties

Strain L227-S17, the uncultured L227-5C strain and the two RCI-encoding environmental MAGs belong to a previously uncultivated order within the Chloroflexota, based on classification according to the Genome Taxonomy Database^46^. We provisionally name this order the ‘*Ca*. Chloroheliales’ (in place of the former taxon name, ‘54-19’; Supplementary Note 3). This novel order places within the same class, Chloroflexia, as the Chloroflexales order that contains RCII-utilizing phototrophs. Although separated from RCII-utilizing phototroph families by the non-phototrophic Herpetosiphonaceae family (in the Chloroflexales order)^47^, the ‘*Ca*. Chloroheliales’ order placed directly sibling and basal to the Chloroflexales order (Fig. 3), suggesting that RCI-utilizing and RCII-utilizing Chloroflexota members are closely related.

**Fig. 5:**
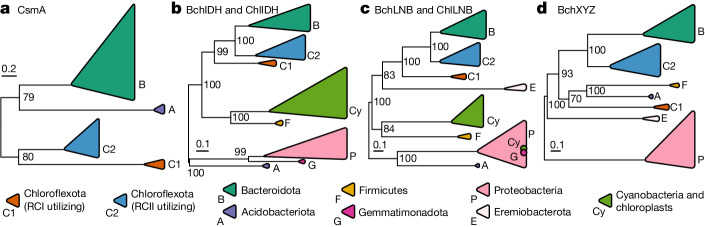
Phylogenetic relationships of photosynthesis-related genes among known phototrophs. **a**–**d**, Maximum-likelihood phylogenies are shown for the chlorosome baseplate-associated protein CsmA (**a**) and for proteins associated with (bacterio)chlorophyll synthesis: BchIDH and ChlIDH (**b**), BchLNB and ChlLNB (**c**) and BchXYZ (**d**). The phylogenies are midpoint rooted, and bootstrap values of greater than 50% are shown. The two dots within the Proteobacteria clade (**c**) indicate placement of some Cyanobacteria and Gemmatimonadota sequences within this clade. Scale bars represent the expected proportion of amino acid change. Detailed versions of these phylogenies, as well as phylogenies of non-concatenated genes, are available in the code repository associated with this work.

We probed the phylogenetic relationships between photosynthesis genes encoded by ‘*Ca*. Chloroheliales’ members and those of other phototrophs to explore their evolutionary relationship (Fig. 5). In a maximum-likelihood phylogeny of the chlorosome structural protein CsmA, the ‘*Ca*. Chloroheliales’ clade placed sibling and basal to the RCII-utilizing Chloroflexota clade (Fig. 5a). Similarly, in maximum-likelihood phylogenies of the (bacterio)chlorophyll synthesis proteins BchIDH and ChlIDH (Fig. 5b) and BchLNB and ChlLNB (Fig. 5c), ‘*Ca*. Chloroheliales’ sequences placed basal to the sister grouping of RCII-utilizing Chloroflexota and RCI-utilizing Bacteroidota (Chlorobiales) members. Placement of ‘*Ca*. Chloroheliales’ was less stable in a maximum-likelihood phylogeny of BchXYZ proteins (Fig. 5d) and varied between individual BchX, BchY and BchZ phylogenies, yet in all cases, the clade placed either directly basal to the RCII-utilizing Chloroflexales and RCI-utilizing Bacteroidota groups or placed as the basal member of a clade adjacent to these groups (as in Fig. 5d). Sequences from RCII-utilizing ‘*Ca*. Thermofonsia’ members, which are thought to have acquired phototrophy by recent lateral gene transfer from Chloroflexales members^24^, grouped together with sequences of RCII-utilizing Chloroflexales members and separately from the novel RCI clade in phylogenies where these sequences were included.

## Evolutionary implications

The sister and basal phylogenetic placement of the RCI-utilizing ‘*Ca*. Chloroheliales’ clade to the RCII-utilizing Chloroflexales clade in both photosynthesis gene trees (Fig. 5) and the Chloroflexota species tree (Fig. 3) provides strong evidence that, despite using different photosynthetic reaction centres, RCI-utilizing and RCII-utilizing Chloroflexota members have shared phototrophic ancestry. Because the relative placement of the RCI-utilizing ‘*Ca*. Chloroheliales’ and RCII-utilizing Chloroflexales clades are consistent between these phylogenies, vertical inheritance from a common ancestor, rather than lateral gene transfer, was probably the dominant factor driving the diversification of phototrophy in the Chloroflexia class. The sister grouping of the Bacteroidota clade and the RCII-utilizing Chloroflexota clade in phylogenies of bacteriochlorophyll synthesis genes (Fig. 5b–d) could be due to a lateral gene transfer event from Chloroflexota members to Bacteroidota members^4^. The most parsimonious explanation for our phylogenomic data is thus that RCI-utilizing and RCII-utilizing Chloroflexia members share a most recent common phototrophic ancestor, which may have encoded genes for bacteriochlorophyll and chlorosome synthesis and at least one class of the photosynthetic reaction centre.

Two scenarios could explain how RCI-utilizing and RCII-utilizing Chloroflexota members could have diversified from a shared phototrophic ancestor. In one scenario, the most recent common phototrophic ancestor of the Chloroflexia encoded a single photosynthetic reaction centre class that was displaced in some descendants by another reaction centre, derived from lateral gene transfer^4^. In the alternative scenario, the most recent common phototrophic ancestor encoded both reaction centre classes, but only one reaction centre class was ultimately retained in known modern clades due to differential gene loss. Either of these ‘genetic displacement’ or ‘differential loss’ scenarios clarifies how chlorosomes came to be encoded by both RCI-utilizing and RCII-utilizing phototrophic Chloroflexota members, because genes associated with chlorosome synthesis could have been derived predominantly vertically by both RCI-utilizing and RCII-utilizing phototrophs from the common ancestor, followed by adaptation. In either scenario, RCI and RCII came to function independently in the conversion of light energy among Chloroflexota members that share a related physiological and genetic background.

## Discussion

Despite only being associated with RCII previously, our combined physiological, genomic and environmental survey data demonstrate that members of the Chloroflexota phylum can use RCI for phototrophic growth. Use of contrasting modes of light energy conversion by related phototrophs has, to our knowledge, never been reported in nature and has substantial implications for photosynthesis research. Understanding the physiological advantages of RCI-based versus RCII-based phototrophy by ‘*Ca*. Chloroheliales’ versus Chloroflexales members could lead to fundamental new insights into the ecology and relative functional properties of reaction centres that are core to photosynthesis. Metabolic flexibility previously observed in RCII-utilizing Chloroflexota members^48^ could be linked to how members of the Chloroflexota phylum adapted to support multiple phototrophic modes. We anticipate that exploring this metabolic flexibility, along with the specific adaptations that allowed contrasting photosynthetic reaction centre classes to function in related genetic backgrounds, will shed new light on the biochemical and genetic basis of phototrophy. Probing the evolutionary history of RCI-based versus RCII-based phototrophy within the Chloroflexota phylum could yield fresh insights into how oxygenic photosynthesis, the only known process to combine RCI and RCII for electron flow, arose and evolved. Indeed, recent phylogenomic data suggesting that oxygen-tolerant form I RuBisCO originated within the Chloroflexota^49,50^ further point to the importance of understanding the connection between the Chloroflexota phylum and oxygenic photosynthesis.

**Fig. 6:**
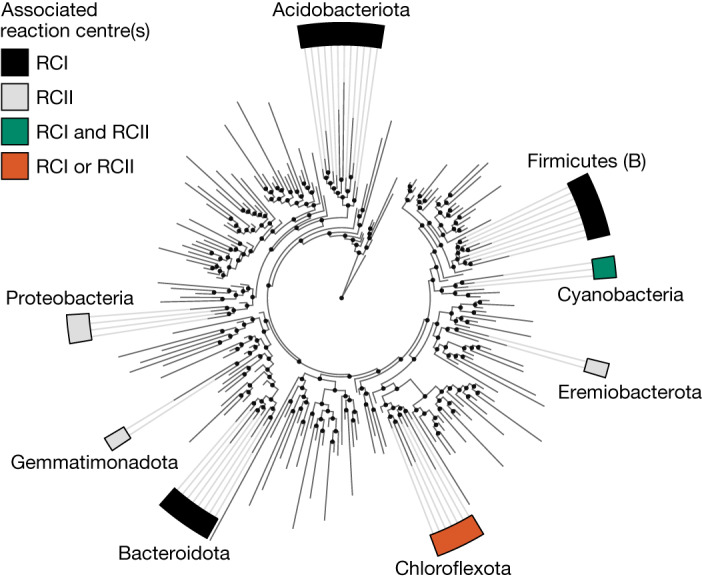
Revised view of the diversity of phototrophic life. Bacterial phyla containing cultured chlorophototrophic representatives are shown. The Genome Taxonomy Database bacterial reference tree (release 89), collapsed at the class level, was used as the phylogeny.

Discovery and characterization of ‘*Ca*. Chlorohelix allophototropha’ strain L227-S17 demonstrates that phototrophy can be driven by solely RCI or solely RCII in related anoxygenic phototrophs and changes our view of the diversity and evolution of phototrophic life (Fig. 6). On the basis of the data that we present, phototrophy within the Chloroflexota represents a unique case study in which enigmatic photosynthesis gene distributions can be resolved through cultivation-based discovery. Existing perspectives on the evolution of photosynthesis will need to be revisited in light of the phototrophic diversity within this phylum, informed by future research investigating the physiology, evolution and ecology of novel RCI-utilizing Chloroflexota phototrophs.

## Species description

*Candidatus* Chlorohelix allophototropha

**Etymology.** Chlo.ro.he’lix. Gr. (Ancient Greek) adj. (adjective) *chloros*, green; Gr. fem. (feminine) n. (noun) *helix*, spiral; N.L. (Neo-Latin) fem. n. *Chlorohelix*, a green spiral. Al.lo.pho.to.tro’pha. Gr. adj. *allos*, other, different; Gr. n. *phos*, *-otos*, light; N.L. suffix *-tropha*, feeding; N.L. fem. adj. *allophototropha*, phototrophic in a different way.

**Locality.** Cultivated from water obtained from the anoxic layer of Lake 227, an iron-rich Boreal Shield lake near Kenora, Ontario, Canada.

**Diagnosis.** Anoxygenic phototrophic bacterium containing bacteriochlorophylls *a* and *c*. Cells are 0.5–0.6 µm wide and 2–10 µm long and grow in curved filaments. Uses a RCI.

## Methods

### Enrichment cultivation

To culture RCI-utilizing Chloroflexota members, we sampled Lake 227 (49.69° N, 93.69° W), a seasonally anoxic and ferruginous Boreal Shield lake at the International Institute for Sustainable Development Experimental Lakes Area (IISD-ELA). The IISD-ELA sampling site (49.50–49.75° N, 93.50–94.00° W), located near Kenora, Ontario, Canada, has been previously described in detail^42,55–58^. Lake 227 develops more than 100 μM concentrations of dissolved iron in its anoxic water column^42^ (Extended Data Fig. 1c), and anoxia is more pronounced than expected naturally due to long-term experimental eutrophication of the lake^43^. We collected water from the illuminated portion of the upper anoxic zone of Lake 227 in September 2017, at depths of 3.88 m and 5.00 m, and transported this water to the laboratory under anoxic and chilled conditions in 120-ml glass serum bottles sealed with black rubber stoppers (Geo-Microbial Technology Company).

Water was supplemented with 2% v/v of a freshwater medium^28^, amended with 8 mM ferrous chloride, and was distributed anoxically into 120-ml glass serum bottles, sealed with black rubber stoppers (Geo-Microbial Technology Company), that had a headspace of dinitrogen (N_2_) gas at a final pressure of 1.5 atm. Bottles were spiked with a final concentration of 50 μM Diuron or 3-(3,4-dichlorophenyl)-1,1-dimethylurea (Sigma-Aldrich) to block oxygenic phototrophic activity^59^. Spiking was performed either at the start of the experiment (for the L227-5C culture, from 5.00-m samples) or as needed following observations of oxygenic phototroph growth (for the L227-S17 culture, from 3.88-m samples). Bottles were incubated at 10 °C under white light (20–30 μmol photons m^−2^ s^−1^; blend of incandescent and fluorescent sources), for experiments that resulted in the enrichment of strain L227-5C, or at 22 °C under far-red LED light (using a PARSource PowerPAR LED bulb; LED Grow Lights Depot) for experiments that resulted in the enrichment of strain L227-S17. Cultures were monitored regularly for ferrous iron concentration using the ferrozine assay^60^ and were amended with additional freshwater medium or ferrous chloride when ferrous iron levels decreased, presumably due to iron oxidation (Extended Data Fig. 1d). Up to three subcultures were performed in liquid medium for cultures that depleted ferrous iron (Supplementary Note 1), and the concentration of the liquid freshwater medium was gradually increased during subculturing to 100% (that is, undiluted) with 8 mM ferrous chloride.

Following initial liquid enrichment, deep agar dilution series^61^ was used to stabilize growth of the L227-S17 culture and to eliminate contaminants. After several rounds of agar cultivation (Supplementary Note 1), the agar-containing medium was adjusted to the following composition. A solution with a final concentration of 2.80 mM ammonium chloride, 1.01 mM magnesium sulfate and 0.34 mM calcium chloride was autoclaved and cooled under N_2_ gas. Sterile potassium phosphate monobasic and sodium bicarbonate were added to a final concentration of 2.20 mM and 11.07 mM, respectively, along with 0.5 ml l^−1^ of trace element solution SLA (optionally selenite-free)^62^, 0.5 ml l^−1^ of a previously published vitamin solution^28^, 0.5 ml l^−1^ of selenite-tungstate solution^63^ and cobalamin to a final concentration of 18.4 nM. Concentrations of calcium pantothenate and thiamine were doubled (to 105 µM and 296 µM, respectively) in the vitamin solution compared with the reference. Resazurin was optionally added, before initial autoclave sterilization, to a final concentration of 2.0 µM. The medium was kept at pH 7.5 and stored under 90:10 or 80:20 N_2_ to carbon dioxide (CO_2_) ratio. We refer to this adjusted freshwater medium as ‘Chx3.1 medium’. This medium was amended with a final concentration of 0.2–0.8% molten agar, 2 mM ferrous chloride and 1.2 mM acetate while preparing agar shake tubes, which were then kept stoppered under a N_2_–CO_2_ headspace. Triple-washed Bacto Agar (Becton, Dickinson and Company) or Agar A (Bio Basic) could be used as the agar source. Unless otherwise noted, all L227-S17 agar cultures in subsequent methods were grown using Chx3.1 medium, including 0.2–0.3% (w/v) agar, 2 mM ferrous chloride and 1.2 mM acetate, and were incubated at 22 °C. We also enriched the main contaminating bacterium in the culture, *Geothrix* sp. L227-G1, and obtained a closed genome bin for this bacterium, as described in the Supplementary Methods.

### Culture physiology

Spectroscopic analyses were carried out on cultures of L227-S17 and other phototrophs. Cultures of L227-S17 were incubated under 735-nm light via an ISL-150 × 150 series LED panel (CCS; a distance of approximately 30 cm at maximum intensity) until green or golden colonies were visible, which were picked and concentrated by centrifugation (Supplementary Methods). Cultures of *C. ferrooxidans* and *C. aurantiacus* were also grown and harvested (Supplementary Methods). To obtain in vivo absorption spectra, culture biomass was sonicated in 10 mM Tris-HCl (pH 8) using a VP-050 ultrasonic homogenizer (TAITEC Corporation). Sonication was performed for 1 min (in approximately 10 s on, 10 s off pulses), followed by 1 min on ice, for five cycles, and resulting crude cell extracts were centrifuged at 10,000*g* for 5 min. Absorption spectra of the supernatant were measured from 300 nm to 1,000 nm using a UV-1800 UV-Visible Scanning Spectrophotometer (Shimadzu). For in vitro measurement of bacteriochlorophyll *c* species, pigments were extracted by gentle disruption of cells diluted at least 1:10 in methanol, followed by centrifugation at 15,000*g* for 10 min. Extracts were then analysed by high-performance liquid chromatography (HPLC) using a Discovery 5-μm C18 column (Supelco). Gradient conditions for the HPLC were as previously described (including a 15 min constant hold of solvent B)^64^. Absorption spectra were measured using a SPD-M10A diode array detector (Shimadzu). In each resulting HPLC profile, all retention time peaks at 667 nm that had more than 3.5% of the prominence of the largest peak were confirmed to have the same associated absorption spectral peaks.

A light–dark growth test was performed on cultures of L227-S17 to test the effect on culture growth. Cultures were incubated for two subculture generations in the light (735 nm, as above) or dark and were tested with or without acetate amendment. Triplicate agar shake tubes (that used 0.2% w/v agar in subculture generation 2) were used for all treatments. Cultures were incubated until green or golden colonies were visible in ‘light’ treatments. To harvest biomass from subculture generation 2 tubes, after discarding approximately the top 5–10% of the tube contents, soft agar from each tube was centrifuged at 12,000*g* for 5 min at 4 °C, followed by removal of the supernatant and an upper agar layer within the pellet. Pellets were washed once in 10 mM Tris-HCl (pH 8) and frozen at −30 °C for microbial community analysis. After DNA extraction (described below), DNA extracts for replicate samples were combined in equal DNA mass ratios for 16S rRNA gene sequencing. For acetate treatments, partial in vivo spectra were generated from a portion of the unfrozen pellet. Sonication was performed as described for in vivo spectra above, except two sonication cycles were used instead of five. Crude cell extracts were then centrifuged at 5,000*g* for 1 min at 4 °C, and the absorption spectrum of the supernatant was recorded from 500 nm to 1,000 nm using a UV-1800 UV-Visible Scanning Spectrophotometer (Shimadzu). Resulting absorption spectra were normalized using linear baseline correction between 650 nm and 850 nm.

To perform transmission electron microscopy, cell biomass was picked from L227-S17 cultures grown under white light (30 µmol photons m^−2^ s^−1^; mix of incandescent and fluorescent sources), and residual agar surrounding cells was digested using agarase. One unit of β-agarase I (New England Biolabs) and 10 µl of 10× reaction buffer was added to 100 μl of cell suspension and incubated at 42 °C for 1.5 h. Following cell pelleting and removal of supernatant, cells were then fixed for 2 h at 4 °C in a solution of 4% glutaraldehyde and 4% paraformaldehyde (dissolved in phosphate-buffered saline) and stored at 4 °C. Sample preparation, including fixation with osmium tetroxide, and imaging were performed at the Molecular and Cellular Imaging Facility of the Advanced Analysis Center (University of Guelph; Supplementary Methods). For scanning electron microscopy, cultures were grown as above but also included 120 µM sulfide. Fixed cells, digested with agarase as above, were prepared and imaged at the Molecular and Cellular Imaging Facility of the Advanced Analysis Center (University of Guelph; Supplementary Methods). For all physiological analyses and enrichment cultures, blinding and randomization were not used, because experimenters needed to be aware of the differences between samples to handle them appropriately, and because sample sizes were small. Statistical methods were not used to predetermine sample sizes.

### Microbial community profiling

To confirm the microbial community composition of enrichment cultures, genomic DNA was extracted from pelleted cell biomass using the DNeasy UltraClean Microbial Kit (Qiagen). For early enrichment cultures (L227-S17 and L227-5C subculture generations 0–1; Supplementary Data 1), a 10 min treatment at 70 °C was performed after adding Solution SL to enhance cell lysis. Resulting DNA extracts were quantified using the Qubit dsDNA HS Assay Kit (Thermo Fisher Scientific). Three different 16S rRNA gene amplicon sequencing methods were then used to analyse enrichment culture samples (Extended Data Table 1). For some cultures, the V4–V5 region of the 16S rRNA gene was amplified from extracted DNA via the universal prokaryotic PCR primers 515F-Y^65^ and 926R^66^ as previously described^67–69^. Library pooling, cleanup and sequencing on a MiSeq System (Illumina) were performed as previously described^67^ to generate 2 × 250-bp paired-end reads. Samples were randomized before PCR to avoid bias when libraries had a sufficiently high sample size (that is, of at least 24 samples; this was the case for early enrichment cultures of L227-S17 and L227-5C, subculture generations 0–1). For other cultures, the V4 region was amplified and sequenced by the Bioengineering Lab Co. using the universal prokaryotic primers 515F^70^ and 806R^70^. Sequencing was performed on a MiSeq System to generate 2 × 300-bp paired-end reads. For a final set of cultures, the 16S Barcoding Kit 1-24 (Oxford Nanopore Technologies) was used to amplify the near full-length 16S rRNA gene (V1–V9 region) using the universal bacterial PCR primers 27F^71^ and 1492R^71^. Resulting libraries were sequenced on a R9.4.1 Flongle flow cell (FLO-FLG001; Oxford Nanopore Technologies) via the MinKNOW software, v21.02.1 or v21.11.7 (Oxford Nanopore Technologies). Basecalling was performed using Guppy v5.0.16 or v5.1.12 (Oxford Nanopore Technologies) via the Super Accuracy model. Randomization and blinding were not performed for Nanopore sequencing due to the small number of samples in each library.

Sequence data analysis was performed for V4–V5 region samples using QIIME2 (v2019.10)^72^ via the AXIOME3 pipeline^73^, commit 1ec1ea6 (https://github.com/neufeld/axiome3), with default parameters. In brief, paired-end reads were trimmed, merged and denoised using DADA2 (v1.10.0)^74^ to generate an amplicon sequence variant (ASV) table. Taxonomic classification of ASVs was performed using QIIME2’s naive Bayes classifier^75^ trained against the SILVA SSU database^76,77^, release 132. The classifier training file was prepared using QIIME2 (v2019.7). Commit e35959d of AXIOME3 was used to analyse a single sample (subculture 13.2a-3) using the same QIIME2 and database versions as described above. For V4 region samples, QIIME2 (v2022.8) was used to analyse the samples directly. After removing forward and reverse PCR primers from the reads using CutAdapt (v4.1)^78^, reads were trimmed on the 3′ ends, merged and denoised using DADA2 (v1.22.0)^74^ to generate an ASV table. For V1–V9 region samples, NanoCLUST commit a09991c (fork: https://github.com/jmtsuji/nanoclust) was used to generate polished 16S rRNA gene sequence clusters^79^, followed by primer trimming, 99% operational taxonomic unit clustering and chimera removal as described in the Supplementary Methods. Resulting amplicon sequences (ASVs or operational taxonomic units) were then classified as strain L227-S17 or *Geothrix* sp. L227-G1 based on a 100% match across their complete sequence to reference 16S rRNA gene sequences generated during genome sequencing for these species. For one deeply sequenced V4 region sample (subculture 21.2; Extended Data Fig. 1f), a one-base mismatch to the strain L227-S17 or *Geothrix* sp. L227-G1 sequence was allowed during classification to assign taxonomy to four low-count ASVs (less than 0.3% relative abundance each) that may represent sequencing artefacts.

### Culture genome and metagenome analysis

The functional gene content of early liquid enrichment cultures (subculture generations 0–1) was assessed via short-read metagenome sequencing. Genomic DNA was extracted as above, and library preparation and sequencing were performed at The Centre for Applied Genomics (TCAG; The Hospital for Sick Children, Toronto, Ontario, Canada). The Nextera DNA Flex Library Prep Kit (Illumina) was used for metagenome library preparation, and libraries were sequenced using a HiSeq 2500 System (Illumina), with 2 × 125-bp paired-end reads, to obtain 5.0–7.3 million read pairs per sample. In addition, a read cloud metagenome of L227-S17 subculture 15.2 was sequenced as described in the Supplementary Methods. The resulting metagenomes were analysed using the ATLAS pipeline (v2.2.0)^80^ to generate a set of dereplicated MAGs, including a MAG of strain L227-5C (Supplementary Methods).

To close the genome of strain L227-S17, a single large colony was picked from an agar shake tube of L227-S17 subculture 19.9, which was grown with 0.4% (w/v) agar under 735-nm light (as for in vivo spectra above) and included an additional 100 µM sulfide. Genomic DNA was extracted from the picked colony (as above), and short-read metagenome sequencing was then performed by the Bioengineering Lab Co. In brief, input DNA was processed using the Nextera XT DNA Library Prep Kit (Illumina) followed by the MGIEasy Circularization Kit (MGI), and the resulting library was sequenced as 2 × 200-bp paired-end reads on a DNBSEQ-G400 (MGI) to generate 3.3 million read pairs. Remaining material from the same DNA extract of subculture 19.9 was then concentrated using the DNA Clean and Concentrator-5 kit (Zymo Research) and used for long-read sequencing. Using 225 ng of the DNA sample, spiked with 150 ng of Lambda DNA (EXP-CTL001; Oxford Nanopore Technologies), a long-read sequencing library was prepared using the Ligation Sequencing Kit (SQK-LSK110; Oxford Nanopore Technologies) with long fragment buffer, followed by sequencing using a R9.4.1 Flongle flow cell (FLO-FLG001; Oxford Nanopore Technologies). Reads matching the Lambda phage genome (NC_001416.1) were depleted during sequencing, using adaptive sampling, via MinKNOW v21.02.1 (Oxford Nanopore Technologies). Basecalling was performed using Guppy 5.0.16 (Oxford Nanopore Technologies) with the super accuracy model, generating 0.51 million reads with a mean length of 2.1 kb.

Long-read and short-read sequencing data from subculture 19.9 were used to generate a hybrid assembly of the strain L227-S17 genome. Nextera sequencing adapters on the 5′ ends of short reads were trimmed using CutAdapt (v3.4)^78^. Short-read quality control was then performed using the ‘qc’ module of ATLAS (v2.8.2)^80^. Within ATLAS, sequencing adapters used during MGI-based short-read library preparation were added to the ‘adapters.fa’ file to facilitate adapter removal. The quality control-processed short-read data were then combined with long-read data to perform hybrid genome assembly and sequence polishing using a custom pipeline that we developed named Rotary (https://github.com/rotary-genomics/rotary, 10.5281/zenodo.6951912), commit e636236 (Supplementary Methods). After running Rotary, single-copy marker genes were identified in the genome using CheckM (v1.0.18)^81^, and the genome was annotated using PGAP (v2022-02-10.build5872)^82^. A whole-genome visualization was constructed using Circos (v0.69.8)^83^, with Biopython (v1.81)^84^ used for GC content and GC skew calculations.

### Identification of RCI-associated genes

We searched for RCI-associated gene homologues in the genomes of strains L227-S17 and L227-5C using hmmsearch (v3.1b2)^85^ and profile hidden Markov models (HMMs) downloaded from Pfam^86^. Genes encoding the RCI (*pscA*, *pshA* or *psaAB*; PF00223), a RCI-associated protein (*Chlorobia*-associated *pscD*; PF10657), chlorosome structural units (*csmAC*; PF02043 and PF11098) and a bacteriochlorophyll *a*-binding protein (*fmoA*; PF02327) were queried. We also queried genes encoding an RCI-associated iron–sulfur protein (*pscB*; PF12838) and RCI-associated *c*-type cytochromes (*Chlorobia*-associated *pscC* or *Heliobacterium*-associated *petJ*; PF10643 or PF13442), involved in photosynthetic electron transport, although HMMs used for these genes were nonspecific. The genomes were confirmed to lack the *pufLM* genes associated with RCII using the ‘Photo_RC’ HMM (PF00124), which targets both the *pufL* and *pufM* genes associated with the RCII core. We used the same HMM set (excluding nonspecific HMMs mentioned above) with an *e* value threshold of 10^−1^ to confirm the lack of photosynthesis-associated marker genes in the *Geothrix* sp. L227-G1 genome.

The tertiary structures associated with *pscA*-like gene homologues, detected in the genomes of strains L227-S17 and L227-5C, were predicted using the I-TASSER web server^87^ (accessed in May 2019 and March 2020, respectively). Conserved [4Fe–4S] cluster-binding sites were identified by sequence alignment (see alignment methods below) and were visualized in JalView (2.11.2.7)^88^. Custom HMMs were also built for the *pscA*-like gene and the *fmoA* gene homologues encoded by the strains. Primary sequences were aligned using Clustal Omega (v1.2.3)^89^, and HMMs were generated using hmmbuild (v3.1b2)^85^. Custom HMMs and homology models generated by I-TASSER are available in the code repository associated with this work.

### Phylogenomics

We compared the phototrophy-related genes of strain L227-S17 with phototrophy-related genes of other Chloroflexota phylum members. Genomes of representative members of the Chloroflexota phylum were collected as described in the Supplementary Methods, and we used this genome set to construct a species tree via GToTree (v1.4.11)^90^ and IQ-TREE (v1.6.9)^91^. Within GToTree, the ‘Bacteria.hmm’ collection of 74 single-copy marker genes was used to generate a concatenated protein sequence alignment, with a minimum threshold of 30% of marker genes per genome. All but two genomes, Chloroflexi RRmetagenome_bin16 and ‘*Ca*. Thermofonsia’ bin CP2-42A, contained greater than 50% of the marker genes. A maximum-likelihood phylogeny was built using the resulting masked multiple sequence alignment via IQ-TREE with the LG + F + R6 evolutionary model, as determined by ModelFinder^92^, and 1,000 ultrafast bootstraps^93^. The length of the masked concatenated sequence alignment was 11,700 residues.

Photosynthesis-associated genes, including genes associated with photosynthetic reaction centres, antenna proteins, chlorosome structure and attachment, bacteriochlorophyll synthesis and carbon fixation were selected based on the genome analyses of Tang and colleagues^38^ and Bryant and colleagues^4^. Reference sequences were identified in the genomes of well-studied representatives of the Chloroflexota phylum, namely, *C. aurantiacus*^11^, *Oscillochloris trichoides*^20^ and *Roseiflexus castenholzii*^94^. Bidirectional BLASTP^95^ was performed against the entire Chloroflexota genome collection, using these reference sequences as queries, to detect potential orthologues. The BackBLAST pipeline^96^, v2.0.0-alpha3 (10.5281/zenodo.3697265), was used for bidirectional BLASTP, and cut-offs for the *e* value, per cent identity and query coverage of hits were empirically optimized to 10^−3^, 20% and 50%, respectively. Separately from the bidirectional BLASTP search, we identified additional genes potentially involved in bacteriochlorophyll synthesis for strain L227-S17 based on ref. ^30^.

In addition to genomic comparisons within the Chloroflexota phylum, we compared key phototrophy genes encoded by strain L227-S17 with those of other known bacterial phototrophs using phylogenetic analysis. Genes associated with RCI (*pscA*, *pshA* or *psaAB*)^97^, bacteriochlorophyll *a* binding (*fmoA*)^37^, chlorosome structure (*csmA*)^98^, (bacterio)chlorophyll synthesis (*bchIDH* and *chlIDH*, *bchLNB* and *chlLNB*, and *bchXYZ*)^4^ and carbon fixation via the reductive pentose phosphate cycle (*rbcL*)^99^ were identified among a diverse set of phototroph reference genomes as described in the Supplementary Methods. Predicted primary sequences were aligned using Clustal Omega (v1.2.3)^89^, followed by manually inspection. Alignments were masked using Gblocks (v0.91b)^100^ with relaxed settings (-t = p -b3 = 40 -b4 = 4 -b5=h) to preserve regions with remote homology. Maximum likelihood protein phylogenies were then built using IQ-TREE (v1.6.9)^91^ with 1,000 rapid bootstraps to calculate branch support values^93^. Evolutionary rate models, identified using ModelFinder^92^, were as follows: LG + F + G4 (RCI), LG + G4 (FmoA), LG + F + G4 (CsmA), LG + F + I + G4 (BchIDH and ChlIDH), LG + F + I + G4 (BchLNB and ChlLNB), LG + I + G4 (BchXYZ) and LG + I + G4 (RbcL). In addition, lengths of masked sequence alignments were 548 (RCI), 356 (FmoA), 74 (CsmA), 1,702 (BchIDH and ChlIDH), 1,034 (BchLNB and ChlLNB), 988 (BchXYZ) and 412 (RbcL) residues.

### Boreal Shield lake survey

We sampled eight seasonally anoxic lakes (lakes 221, 222, 224, 227, 304, 373, 442 and 626) within the IISD-ELA, along with a permanently oxic reference lake (Lake 239). The water columns of the lakes were sampled in the summer or autumn of 2016–2018 across four main sampling events (Supplementary Methods). Samples for water column DNA were collected by pumping water, using a closed system gear pump and line, through sterile 0.22-µm Sterivex polyvinyl fluoride filters (Merck Millipore). Water column RNA samples were collected similarly, except filter cartridges were filled immediately with 1.8 ml of DNA/RNA Shield (Zymo Research) once packed and purged of residual water. Filters were collected (and subsequently extracted and analysed) in triplicate for RNA. Sterivex filters were kept chilled after collection until being frozen (at −20 °C) upon return to the sampling camp the same day. Filters were then shipped chilled to the University of Waterloo and were kept frozen (at −20 °C) until processing.

Environmental DNA was extracted from the excised membranes of Sterivex filters using the DNeasy PowerSoil or DNeasy PowerSoil HTP 96 Kit (Qiagen). Environmental RNA extraction was performed using the ZymoBIOMICS DNA/RNA Miniprep Kit (Zymo Research) via the ‘DNA & RNA parallel purification’ protocol with in-column DNase I treatment. Modifications to the standard kit protocols for use of Sterivex filters are described in the Supplementary Methods. Samples were randomized before DNA extraction, but randomization was not performed before RNA extraction due to the small number of samples to be processed. Statistical methods were not used to predetermine sample sizes.

### Metagenome and metatranscriptome analysis

Metagenome sequencing was performed for June and September 2016 lake samples by the US Department of Energy Joint Genome Institute (Lawrence Berkeley National Laboratory). The Nextera XT DNA Library Preparation Kit (Illumina; including library amplification steps) was used, followed by 2 × 150-bp paired-end read sequencing, using a HiSeq 2500 System, to generate 44.1–136.8 million read pairs per sample. Metagenome sequencing for 2017 and 2018 field samples was performed by the McMaster Genome Facility (McMaster University, Hamilton, Ontario, Canada). Sequencing libraries were constructed using the NEBNext Ultra II DNA Library Prep Kit for Illumina (New England Biolabs), including library amplification steps, using input DNA sheared with an ultrasonicator (Covaris). The resulting library was sequenced using a HiSeq 2500 System with 2 × 200-bp paired-end reads, followed by a second sequencing run using a HiSeq 2500 System with 2 × 250-bp paired-end reads. Reverse reads for the 2 × 200-bp run were truncated at 114 bp due to a sequencer error. After pooling of data from both runs, a total of 20.6–64.1 million read pairs were generated per sample. Metatranscriptome sequencing for 2017 and 2018 field sampling was also performed by the McMaster Genome Facility (McMaster University). Following rRNA depletion using the Ribo-Zero rRNA Removal Kit (Bacteria; Illumina), library preparation was performed using the NEBNext Ultra II RNA Library Prep Kit for Illumina (New England Biolabs) using normalized RNA inputs per sample and without direction RNA selection. The resulting library was sequenced on a portion of a lane of a HiSeq 2500 System in rapid run mode with 2 × 200-bp paired-end reads. This was the same sequencing run as for 2017–2018 metagenomes. Sequencing generated 5.5–10.2 million read pairs per replicate. In total, 37 metagenomes were sequenced, along with 9 metatranscriptomes representing 3 samples (see Extended Data Table 3 and Supplementary Data 4 and 5).

All environmental metagenome data were processed using the ATLAS pipeline (v2.1.4)^80^. Default settings were used except that the minimum per cent identity threshold for read mapping (via ‘contig_min_id’) was set to 99%, and only MaxBin 2 (v2.2.4) and MetaBAT2 (v2.12.1) were used as binning algorithms^101,102^. To enhance genome binning quality, six lake metagenomes that were previously sequenced from the water columns of lakes 227 and 442 (ref. ^103^) were included in the ATLAS run, along with a single metagenome from the aphotic zone of the nearby and meromictic Lake 111, which was sampled in July 2018 and was not analysed further in the context of this work. The entire ATLAS pipeline, including quality control on raw reads, metagenome assembly of individual samples, metagenome binning, dereplication of bins from all samples and bin analysis, and gene clustering and annotation, was run end-to-end. For the genome-binning step, all metagenome samples from the same lake were summarized in the same ‘BinGroup’, allowing for differential abundance information between samples from the same lakes to be used to guide genome binning. After running ATLAS, dereplicated MAGs were taxonomically classified using the GTDB-Tk (v0.3.2)^104^, which relied on the GTDB (release 89)^46^. (Taxonomic names used in the GTDB release 89 are used throughout this work for consistency.) All MAGs had a minimum completeness of 50% and maximum contamination of 10% based on CheckM (v1.0.7)^81^; the average completeness and contamination of the dereplicated MAGs were 82.7% and 2.1%, respectively. The relative abundance of each MAG in a sample was calculated by dividing the number of quality control-processed reads mapped to the MAG by the total number of assembled reads (that is, raw reads that mapped to assembled contigs) for that sample.

Metagenomes were also searched at the unassembled read level for *pscA*-like genes similar to those used by strains L227-S17 and L227-5C. Short peptide sequences were predicted using FragGeneScanPlusPlus^105^ commit 471fdf7 (fork: https://github.com/LeeBergstrand/FragGeneScanPlusPlus), via the ‘illumina_10’ model, from forward (R1) metagenome reads that passed the quality control module of ATLAS (above). Peptide sequences were searched using the custom HMM developed in this study for PscA via hmmsearch (3.3.2)^85^ with an *e* value cut-off of 10^−10^. Raw hits were then filtered using a BLASTP^95^ search (BLAST v2.10.1) against the PscA-like sequences of strains L227-S17 and L227-5C. Only hits with more than 90% identity and with an *e* < 10^−10^ were retained. Predicted short peptide sequences were also searched using a HMM for the taxonomic marker gene *rpoB* from FunGene (June 2009 version)^106^ via hmmsearch (3.3.2)^85^ with a cut-off *e* value of 10^−10^. Counts of filtered PscA-like hits per metagenome were normalized to counts of RpoB hits following normalization by HMM length. Singleton PscA-like hits were excluded from the analysis.

Metatranscriptome data were processed using the ATLAS pipeline^80^. The ‘qc’ module of ATLAS commit 59da38f was run to perform quality control of raw read data. Then, a customized fork of ATLAS, commit 96e47df (available under the ‘maprna’ branch at https://github.com/jmtsuji/atlas), was used to map RNA reads onto the set of dereplicated MAGs obtained from metagenome analyses (above) and to summarize RNA read counts. In brief, the dereplicated MAGs were used as input for the ‘genomes’ module of ATLAS so that quality control-processed metatranscriptome reads were mapped onto the MAGs using BBMap (v37.78; Bushnell B., https://sourceforge.net/projects/bbmap/). The minimum per cent identity threshold for read mapping (‘contig_min_id’) was set to 99%. Following read mapping to MAGs, the counts of metatranscriptome read hits to genes within MAGs were summarized using featureCounts^107^, in v1.6.4 of the Subread package. Default settings were used, except for the following flags: ‘-t CDS -g ID --donotsort’. The raw analysis code is available in the GitHub repository associated with this work.

After generating RNA read mapping data via ATLAS, the relative expression of each dereplicated MAG was calculated within each metatranscriptome. To perform this calculation, the number of RNA reads that mapped to each MAG was divided by the total number of RNA reads that mapped to all MAGs. The resulting relative expression values were averaged between replicate metatranscriptomes. In addition, we calculated the expression levels of genes associated with RCI-encoding Chloroflexota MAGs based on normalization to *dnaK* and normalization by gene length, and we averaged gene expression levels between replicate metatranscriptomes (Supplementary Methods).

### Ethics and inclusion statement

All field samples were obtained from the IISD-ELA in northwestern Ontario (Canada) and in partnership with IISD-ELA staff. The IISD-ELA engages and partners with local and regional communities as described at https://www.iisd.org/ela. Researchers affiliated with Canadian institutions led the research project from study design to implementation, own the majority of the data and intellectual property generated from this project, and are co-authors on this work. Field safety training and risk management plans were implemented before all field work. Local research is cited as part of this study.

### Reporting summary

Further information on research design is available in the Nature Portfolio Reporting Summary linked to this article.

## Online content

Any methods, additional references, Nature Portfolio reporting summaries, source data, extended data, supplementary information, acknowledgements, peer review information; details of author contributions and competing interests; and statements of data and code availability are available at 10.1038/s41586-024-07180-y.

### Supplementary information

Supplementary Information This file contains Supplementary Figures 1–2, Supplementary Notes 1–3, and Supplementary Methods.

Reporting Summary

Peer Review File

Supplementary Data 1 Amplicon sequencing variant table of early phototroph enrichment cultures from this study

Supplementary Data 2 Bidirectional BLASTP results for photosynthesis-related genes among the Chloroflexota phylum

Supplementary Data 3 Physicochemical parameters for Boreal Shield lake samples

Supplementary Data 4 Mapping of metagenome reads to metagenome-assembled genomes

Supplementary Data 5 Mapping of metatranscriptome reads to metagenome-assembled genomes

Supplementary Data 6 Gene expression of “Ca. Chloroheliales”-associated genome bins

### Source data

Source Data Fig. 1

Source Data Fig. 3

Source Data Fig. 4

## Figures and Tables

**Extended Data Table 1:**
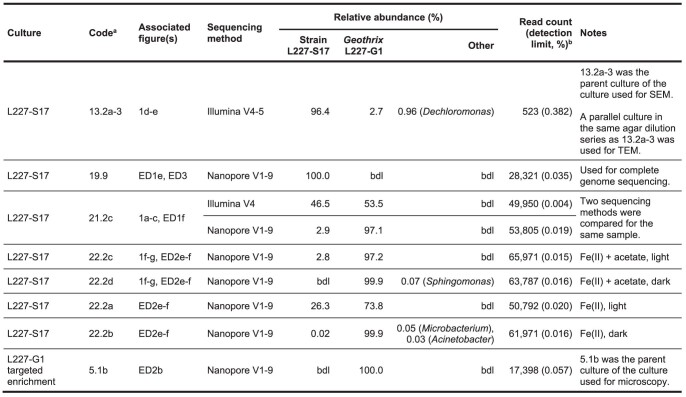
Microbial community composition of enrichment cultures of strain L227-S17 and *Geothrix* sp. L227-G1

The 16S rRNA gene amplicon data accompany spectroscopy, microscopy, and genomic analyses presented in this work. Sequences (i.e., ASVs or OTUs) were assigned as strain L227-S17 or *Geothrix* sp. L227-G1 as described in the Methods. Other ASVs or OTUs are presented in the “Other” category with their genus-level classification. Additional microbial community composition data, based on metagenome reads, are shown in Extended Data Fig. 1e. Abbreviations: bdl below detection limit. Footnotes: ^a^The number before the period (e.g., 12) refers to the subculture generation; subsequent numbers and letters were used internally to distinguish between cultures. ^b^A sequence was detectable if it generated at least 2 counts (Illumina) or 10 counts (Nanopore).

**Extended Data Table 2:**
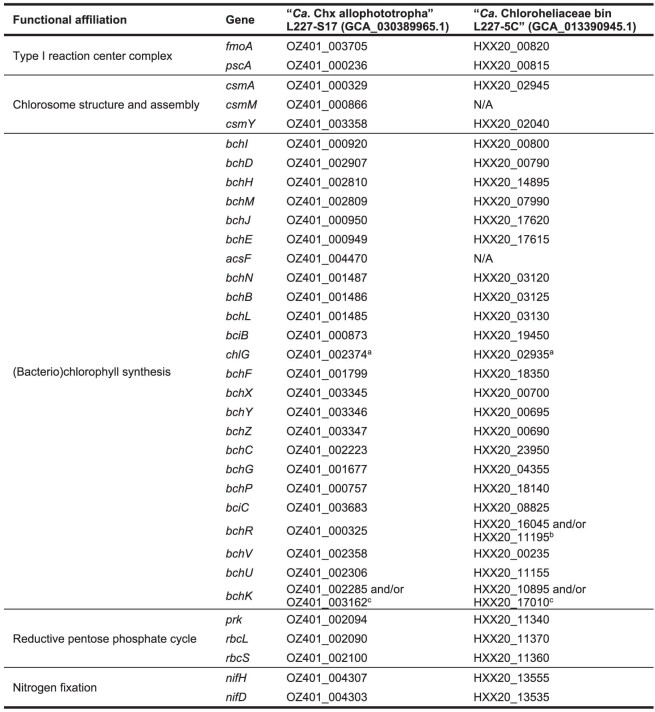
Phototrophy-related genes in the genomes of strains L227-S17 and L227-5C

Locus tags are shown for each gene. Results correspond to those shown in Fig. 3, except that homologs associated with the incomplete 3-hydroxypropionate bicycle are omitted for clarity, and additional genes involved in bacteriochlorophyll synthesis and the RPP cycle are shown. Footnotes: ^a^Assignment of the detected genes as *chlG* (chlorophyll *a* synthase) is uncertain; ^b^Two possible homologs of *bchR* were detected in the L227-5C genome; ^c^Two possible homologs of *bchK* were detected in the L227-S17 and L227-5C genomes.

**Extended Data Table 3:**
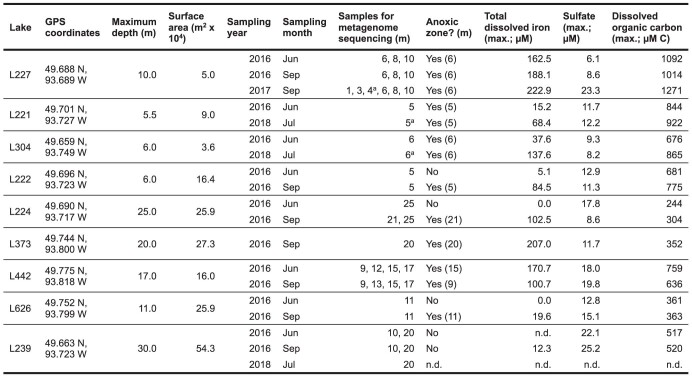
Summary of physicochemical parameters for sampled Boreal Shield lakes

The table includes the location, depth, and surface area of all nine sampled lakes, along with information on the depths sampled for metagenome and metatranscriptome sequencing between 2016−2018. Summary parameters on the right side of the table show the topmost depth (sampled for metagenome sequencing) where dissolved oxygen was undetectable and the maximum measured concentrations of total dissolved iron, sulfate, and dissolved organic carbon among the collected samples. Full physicochemical data are provided in Supplementary Data 3. Abbreviations: max. maximum; n.d. no data. Footnotes: ^a^Samples were also collected and used for metatranscriptome sequencing.

## Data Availability

Enrichment culture metagenomes and MAGs from the L227-S17 culture (subcultures 1 and 15.2) and the L227-5C (primary enrichment) culture are available under NCBI BioProject accession PRJNA640240. Amplicon sequencing data are available at the same BioProject accession. The complete strain L227-S17 genome, along with associated raw read and amplicon sequencing data, are available at BioProject accession PRJNA909349. Similarly, the complete *Geothrix* sp. L227-G1 genome and associated long-read data (subculture 15.c) are available at BioProject accession PRJNA975665. Metagenome data from 2016, sequenced by the JGI, are available in the JGI Genome Portal under Proposal ID 502896. Environmental metagenome and metatranscriptome data from 2017 to 2018 are available under NCBI BioProject accession PRJNA664486. The full set of 756 MAGs used for read mapping of metatranscriptome data are available at BioProject accession PRJNA1003647; genome and annotation versions used for read mapping are available in a Zenodo repository (10.5281/zenodo.3930110). The SILVA SSU database (release 132) and the Genome Taxonomy Database (release 89) are available at https://www.arb-silva.de/download/archive/ and https://data.gtdb.ecogenomic.org/releases/, respectively. In addition, the NCBI Protein Reference Sequences (RefSeq) database and 16S rRNA gene database for Bacteria-type and Archaea-type strains are both available at https://ftp.ncbi.nlm.nih.gov/blast/db/; taxonomy mapping information is available at https://ftp.ncbi.nih.gov/pub/taxonomy/. Source data are provided with this paper.

## Code availability

Custom scripts and additional raw data files used to analyse the metagenome and genome data are available at https://github.com/jmtsuji/Ca-Chlorohelix-allophototropha-RCI (10.5281/zenodo.3932366). A genome assembly pipeline named Rotary, developed for this project, is available at https://github.com/rotary-genomics/rotary (10.5281/zenodo.6951912).
