# Supplementary material for: Anoxygenic phototroph of the Chloroflexota uses a type I reaction centre
Source: Nature. 2024 Mar 13;627(8005):915–22. doi: 10.1038/s41586-024-07180-y (PMC10972752; doi:10.1038/s41586-024-07180-y)
Supplement: Supplementary file 1 — This file contains Supplementary Figures 1–2, Supplementary Notes 1–3, and Supplementary Methods. [file 41586_2024_7180_MOESM1_ESM.pdf]

nature portfolio

https://doi.org/10.1038/s41586-024-07180-y

Supplementary information

Anoxygenic phototroph of the Chloroflexota uses a type I reaction centre

In the format provided by the authors and unedited

Supplementary Information for:

Anoxygenic phototroph of the *Chloroflexota* uses a Type I reaction center

Tsuji JM[1,2,3]*, Shaw NA[1], Nagashima S[4†], Venkiteswaran JJ[1,5], Schiff SL[1], Watanabe T[2], Fukui M[2], Hanada S[4‡], Tank M[4,6], Neufeld JD[1]*

[1]University of Waterloo, 200 University Avenue West, Waterloo, Ontario, Canada, N2L 3G1
[2]Institute of Low Temperature Science, Hokkaido University, Kita-19, Nishi-8, Kita-ku, Sapporo, Japan, 060-0819
[3]Japan Agency for Marine-Earth Science and Technology, 2-15 Natsushima, Yokosuka, Kanagawa, Japan, 237-0061
[4]Tokyo Metropolitan University, 1-1 Minami-osawa, Hachioji, Tokyo, Japan, 192-0397
[5]Wilfrid Laurier University, 75 University Avenue West, Waterloo, Ontario, Canada, N2L 3C5
[6]Leibniz Institute DSMZ-German Collection of Microorganisms and Cell Cultures GmbH, Inhoffenstrasse 7B, 38124 Braunschweig, Germany

[†]Current address: Kanagawa University, 3-27-1 Rokkakubashi, Kanagawa Ward, Yokohama, Kanagawa, Japan, 221-8686
[‡]Current address: Bioproduction Research Institute, National Institute of Advanced Industrial Science and Technology (AIST), Tsukuba Central 6, 1-1-1 Higashi, Tsukuba, Ibaraki, Japan, 305-8566
*e-mail: jackson.tsuji@uwaterloo.ca; jneufeld@uwaterloo.ca

Supplementary Information

This PDF file contains Supplementary Figures 1-2, Supplementary Notes 1-3, and Supplementary Methods.

Contents

Supplementary Figures 2
- Supplementary Fig. 1 2
- Supplementary Fig. 2 3

Supplementary Notes 4
- Supplementary Note 1 4
- Supplementary Note 2 5
- Supplementary Note 3 7

Supplementary Methods 8
- Enrichment of *Geothrix* L227-G1 8
- Selection against sulfate-reducing bacteria in enrichment cultures 9
- Biomass collection for spectroscopy 9
- Identification of bacteriochlorophyll *a* 9
- Electron microscopy 10
- Long read amplicon sequence analysis 10
- Read cloud metagenome sequencing 11
- Culture metagenome analysis 12
- Manual genome bin curation 12
- Hybrid genome assembly 13
- *Geothrix* L227-G1 genome analysis 14
- Metagenome-based community comparisons 14
- Collection of reference genomes/genes 15
- Environmental RCI-associated gene search 16
- Electron transport gene identification 16
- (Bacterio)chlorophyll synthase phylogeny 16
- Lake sampling 17
- Environmental DNA/RNA extraction 17
- Gene expression calculations 18
- Geospatial data processing 19

Supplementary Figures

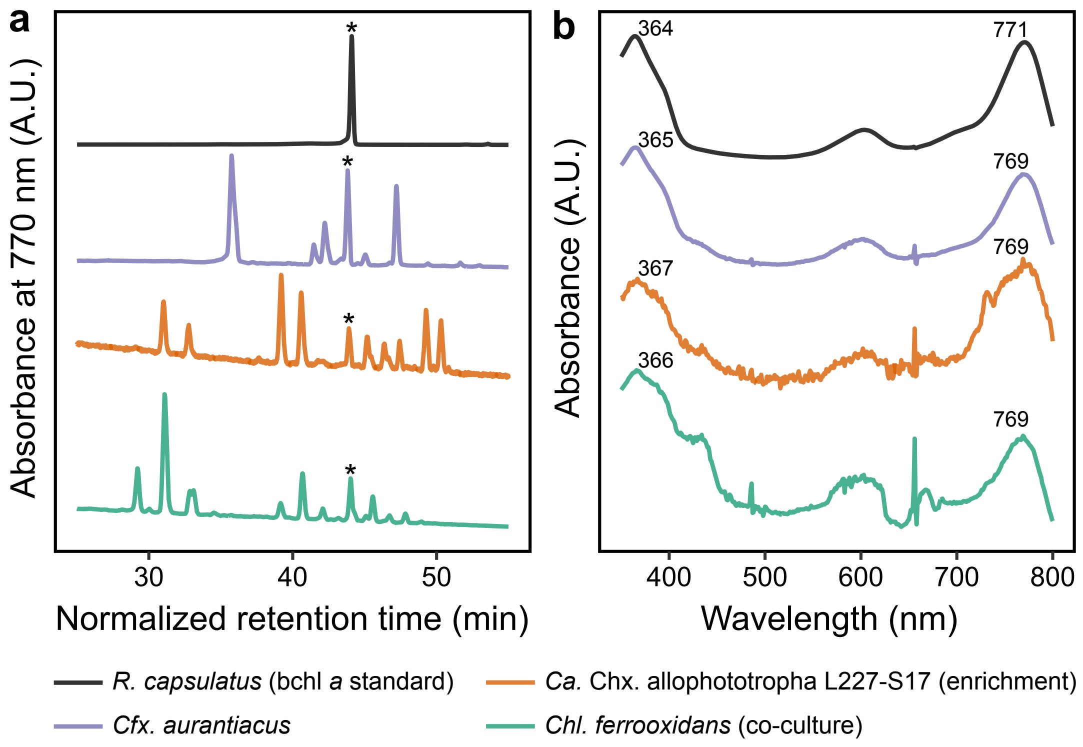

55 **Supplementary Fig. 1 | Identification of bacteriochlorophyll *a* in the L227-S17 culture. a**, HPLC profiles of absorbance at 770 nm. The HPLC profiles are the same as shown at an absorbance of 667 nm in Fig. 1b, except a sample of *Rhodobacter capsulatus* was added as a bacteriochlorophyll *a* standard. **b**, *In vivo* absorption spectra associated with HPLC peaks marked with asterisks in **a**. Sharp absorption peaks at 485-486 nm and 655-656 nm are anomalies associated with the HPLC system.

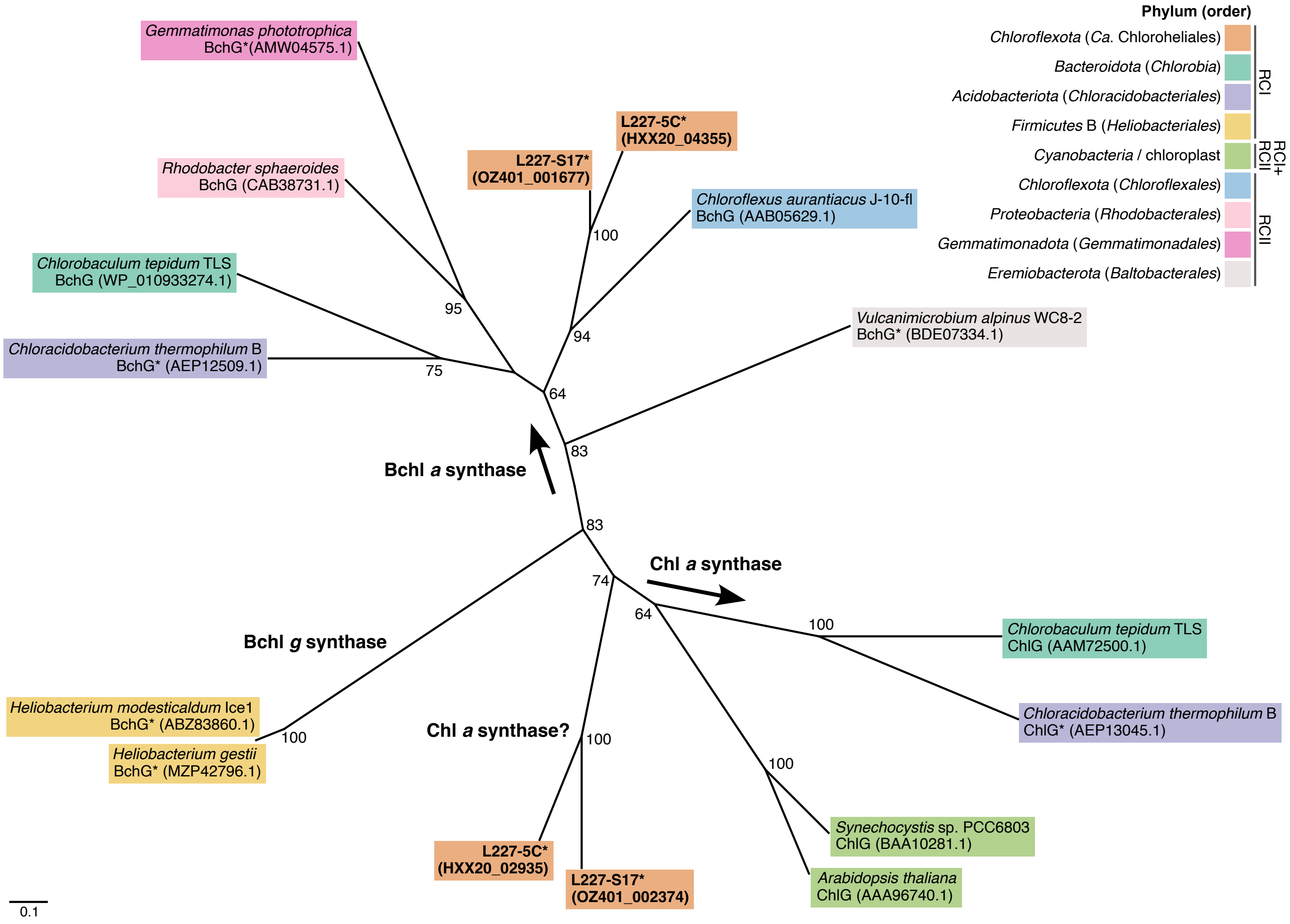

Supplementary Fig. 2 | Possible chlorophyll *a* synthase encoded by strain L227-S17. The maximum likelihood sequence phylogeny shows (bacterio)chlorophyll synthase genes associated with synthesis of chlorophyll *a*, bacteriochlorophyll *a*, and bacteriochlorophyll *g* from representatives of known phototroph-containing phyla. Sequence names followed by an asterisk indicate that the sequence was identified by homology rather than based on a direct reference in the literature. Bootstrap values over 50% are shown. The scale bar represents the expected proportion of amino acid change.

Supplementary Notes

Supplementary Note 1

Growth properties and metagenome-assembled genomes of enriched "*Ca.* Chloroheliales" members. Liquid medium was used for the initial enrichments of strain L227-S17. However, filamentous phototrophs eventually ceased growth or were overtaken by purple phototrophic bacteria in subcultures made in liquid medium. Transfer of enrichment cultures to agar-containing medium allowed us to continue to culture strain L272-S17. As inoculum into agar-containing medium, we ultimately had to use a liquid enrichment (subculture generation 3) of the L227-S17 strain that had been stored at 4°C (dark) for five months, instead of an active culture that had been subcultured in liquid medium for additional generations, due to poor growth of the active culture. Initial agar shake tubes included 10 mM ferrous chloride-containing agar plugs in the bottom of the tubes to form an iron concentration gradient, and these plugs were overlaid with a freshwater medium[28] mixed with 0.6% (w/v) Bacto Agar (Becton, Dickinson and Company). We qualitatively determined favourable ferrous iron concentrations for growth of the strain by observing the growth of colonies along the agar concentration gradient. Medium used for early agar shake tubes also included 6 mM of an acetate feeding solution[62], 50 µL L^{-1} of an additional vitamin solution[108], and 1.1 mM sodium thioglycolate as a reducing agent, although we eventually discontinued use of the additional vitamin solution and sodium thioglycolate due to a lack of observable benefit to culture growth. Tubes were initially incubated at 18°C under halogen light (<50 µmol photons m^{-2} s^{-1}). Further adjustment of these conditions over subsequent subcultures led us to develop Chx3.1 medium as described in the Methods.

During growth in deep agar dilution series, additional selective conditions were used to purify the culture of specific contaminants. To eliminate purple phototrophic bacteria from the culture related to the metabolically versatile *Rhodopseudomonas palustris*[109], agar tubes were incubated under 740 nm LED lights, because light at ~740 nm wavelength is not readily used for photosynthesis by these bacteria[110]. Similar far-red LED lights were also used as a general light source for the L227-S17 culture in later subcultures. The medium was adjusted to an elevated pH of ~7.5-8.5 for a time to select against green phototrophic bacteria belonging to the *Chlorobia* class, because these bacteria are known to grow poorly in moderately basic conditions[111]. Lastly, sodium molybdate was added to the medium at a final concentration of 100 µM, in a 1:10 concentration ratio relative to sulfate, to inhibit the activity of sulfate-reducing bacteria as optimized experimentally (Supplementary Methods). In addition, in some deep agar dilution series cultures, a carbonate-buffered sulfide feeding solution[62] was added at a final sulfide concentration of 100-120 µM to decrease the redox potential of the medium, although we did not observe an effect of this treatment on culture growth.

Physiologically, strain L227-S17 had several shared characteristics with other cultured and RCII-utilizing phototrophs belonging to the *Chloroflexota* phylum. Cells grew well in soft agar and developed as long, spiralling filaments, similar to those observed for *Chloronema* spp., a group of poorly studied *Chloroflexota* members characterized primarily by microscopy from stratified lakes[112]. Although gliding motility of strain L227-S17 cells was not observed, cells formed tight clumps, like *Chloroflexus aggregans*[19], that would readily bind to glass surfaces or to precipitates in culture

medium. We were unable to grow strain L227-S17 in freshwater medium amended with 0.5-3 mM sulfide in place of ferrous iron.

Although strain L227-5C was lost after the initial enrichment from L227, we recovered a high completeness but fragmented MAG of strain L227-5C from an enrichment culture metagenome. Following manual curation, the MAG was estimated by CheckM to have a completeness and contamination of 96.3% and 1.6%, respectively, and was composed of 546 contigs with a total length of 6.31 Mb and average GC content of 47.7 %. The MAG encoded 4794 genes, including 79 tRNA genes and a full-length 16S and 23S rRNA gene, and had a relative abundance of 12.3% within the enrichment culture metagenome based on recruitment of QC-processed short reads (Extended Data Fig. 1e). Three non-*Chloroflexota* genome bins were also recovered from the metagenome, including a RCII-encoding phototroph, classified to the *Rhodoplanes* genus, that likely corresponded to the purple phototrophic bacterium observed in the culture and had a relative abundance of 29.6% in the metagenome (Extended Data Fig. 1e). Genome bins classified to the *Pelobacter* genus and the CG2-30-32-10 family (in the *Bacteroidales* order), that had relative abundances of 4.2% and 3.8% in metagenome, respectively, were also recovered (Extended Data Fig. 1e).

Supplementary Note 2

Additional details on genomic potential for phototrophy by strain L227-S17. The FmoA predicted protein detected in the genome of strain L227-S17 had 21-26% amino acid identity to known FmoA sequences. This protein notably lacked the Cys49 and Cys353 residues previously thought to be necessary for quenching energy transfer to RCI[113]. Similarly, the possible CsmA homolog encoded by strain L227-S17 had only 33% amino acid identity to the CsmA primary sequence of *Chloroflexus aurantiacus*[4]. The predicted CsmA primary sequence was missing the His25 previously thought to be involved in bacteriochlorophyll *a* binding[98]. Biochemical characterization of FmoA and CsmA used by strain L227-S17 is needed to confirm their functional properties and could reveal novel molecular mechanisms involved in phototrophy.

We explored potential electron transport pathways used by strain L227-S17 by searching the genome sequence of the strain and by analyzing the gene expression, in Boreal Shield lake metatranscriptome data, of the related "*Ca.* Chlorohelix bin ELA319". Using reciprocal best hit amino acid sequence searches, followed by manual curation, we were able to associate 3073 of 4996 protein-coding genes of strain L227-S17 with likely orthologs in bin ELA319 (Supplementary Data 6). We then sought, where possible, to identify electron transport gene homologs in strain L227-S17 whose equivalent genes in bin ELA319 were upregulated in Lake 221 and Lake 304 metatranscriptome data, given that ELA319 was phototrophically active in both lakes at the time of sampling (Fig. 4e and Extended Data Fig. 7).

In other known RCI-utilizing phototrophs, noncyclic photosynthetic electron flow involves a large protein complex, analogous to Complex III in mitochondria, that transfers electrons to a RCI electron donor. Cytochrome bc_1 or b_6f complexes or Alternative Complex III have been identified in characterized RCI-utilizing anoxygenic and oxygenic phototrophs[4,114], and RCII-utilizing phototrophic *Chloroflexota* members also encode Alternative Complex III[38]. Searching the strain L227-S17 genome,

we detected a *petXCDB* gene cluster (GenBank locus tags OZ401_002244 to OZ401_002247) that was homologous to the *Heliobacterium modesticaldum petXDBC* gene cluster, which encodes a cytochrome b_6f-like complex[39,115,116]. Because most of this gene cluster was missing in bin ELA319, we could not verify its expression in environmental data. The large protein complex typically transfers electrons to a RCI electron donor protein, such as a soluble c_2 cytochrome (e.g., cytochrome c_{551})[4,117], a membrane-bound cytochrome (e.g., cytochrome c_{553})[115], or plastocyanin, a blue copper protein[38]. The RCII-utilizing *Chloroflexota* member *Chloroflexus aurantiacus* uses the blue copper protein auracyanin for electron transfer to RCII[38]. Searching the strain L227-S17 genome, we were unable to detect clear orthologs to any of these known electron donor proteins used by oxygenic phototrophs or by *Chlorobiales*, *Chloracidobacteriales*, *Heliobacteriales* or RCII-utilizing *Chloroflexales* members. Although we did identify a gene (locus OZ401_003595) encoding a possible membrane-bound cytochrome with distant homology to the cytochrome c_{553} (PetJ) of *Heliobacterium gestii*, the corresponding gene encoded by bin ELA319 was highly downregulated in metatranscriptome data (no detectable transcripts). Given that non-homologous RCI electron donor proteins are used in each known RCI-utilizing phototroph lineage, it is possible that a novel protein is used for this function by strain L227-S17.

Within RCI, in known phototrophs, a special (bacterio)chlorophyll pair serves as the primary electron donor, and after transfer through an internal pigment-containing electron transport chain (discussed in [36,118]), electrons are donated to a [4Fe-4S] cluster, termed F_X, that is also bound by the RCI core protein dimer. Detection of bacteriochlorophyll *a*, which could potentially serve as the primary RCI electron donor, in the L227-S17 culture, as well as identification of the [4Fe-4S] cluster binding site in the RCI primary sequence, are discussed in the main text. As described in the main text, an additional (bacterio)chlorophyll synthase was identified in the strain L227-S17 genome that is phylogenetically related to both chlorophyll *a* and bacteriochlorophyll *g* synthases of known phototrophs (Supplementary Fig. 2); it is possible that pigments produced by this synthase are also involved in the RCI electron transport chain. Following RCI, electrons are transferred from F_X to two additional [4Fe-4S] clusters, F_A and F_B, that, with the exception of *Heliobacteriales* members[119], are held by a tightly bound electron acceptor protein. We identified a candidate protein (encoded by OZ401_003239), with distant homology to PscB used by *Chlorobiales* members[34], that could potentially serve as the RCI terminal electron acceptor in strain L227-S17. This protein includes the expected eight cysteine residues involved in Fe-S cluster binding[13], and the equivalent associated gene in bin ELA319 was upregulated relative to *dnaK* in metatranscriptome data (with $\log_2$-fold expression ratios of 1.57 ± 0.30 and 1.33 ± 0.35 in Lakes 221 and 304, respectively).

The terminal electron acceptor protein of RCI donates electrons to ferredoxins (Fd)[34]. Within the predicted proteins of strain L227-S17, we identified multiple potential ferredoxins, including one ferredoxin (locus OZ401_001166), with distant homology to PshBI/PshBII used by *Heliobacteriales* members, whose equivalent associated gene in bin ELA319 was highly upregulated based on metatranscriptome data (with $\log_2$-fold expression ratios, compared to *dnaK*, of 2.84 ± 0.14 and 2.00 ± 0.36 in Lakes 221 and 304, respectively). High-energy electrons from ferredoxin can be used for the generation of reducing equivalents, such as NADPH, for carbon fixation. Generation of NADPH is carried out by a Ferredoxin—NADP+ reductase (FNR), either a canonical plant-type FNR, as used by

oxygenic phototrophs, or by a NADPH-dependent thioredoxin reductase (TrxR)-like FNR, as used by *Chlorobiales* members[120]. We were unable to identify a strong candidate gene for FNR in strain L227-S17 with potential orthology to known FNR sequences. Although we detected one gene (locus OZ401_003522) with distant homology to the *Chlorobiales* TrxR-like FNR, expression of the equivalent gene in bin ELA319 was highly downregulated compared to *dnaK* (-2.26 ± 1.70 $\log_2$-fold expression ratio in Lake 221; no detected transcripts in Lake 304). Similarly to how *Chlorobiales* members and oxygenic phototrophs use unrelated FAD-dependent oxidoreductases as FNR, it is possible that strain L227-S17 also uses a novel and yet-unidentified form of FNR. We note that one FAD-dependent oxidoreductase, annotated as a glutamate synthase, was moderately expressed in bin ELA319 (with $\log_2$-fold expression ratios compared to *dnaK* of 0.72 ± 0.26 and 0.40 ± 0.44 in Lake 221 and Lake 304, respectively) and corresponds to gene locus OZ401_003240 in strain L227-S17, which neighbours the candidate *pscB*-like gene described above. Given the diverse proteins involved in photosynthetic electron transport and known examples where functionally equivalent proteins have evolved convergently to perform electron transfer steps[117], biochemical experiments will be needed to validate the photosynthetic electron transport chain of strain L227-S17.

In addition to photosynthetic electron transport, we searched for the genomic potential for other electron transport systems in strain L227-S17. Like has been identified in the genome of the RCII-utilizing *Chloroflexus aurantiacus*[38], we identified two gene sets that potentially encode the NADH-quinone oxidoreductase Complex I. One set of genes was divided into two distantly separated clusters on Chr1 (*nuoA*-*nuoF* at locus OZ401_000336-OZ401_000341, and *nuoG*-*nuoN* at locus OZ401_000998- OZ401_000991) that together encoded the complete 14-gene set expected for Complex I. The second gene set was located in a single cluster, also on Chr1, but encoded only 11 genes (OZ401_002520-OZ401_002530), lacking *nuoE*-*nuoG*. The lack of *nuoG* in one of the Complex I gene clusters is common with the genome of *Chloroflexus aurantiacus*[38]. Although genes from the first 14-gene set were highly downregulated based on mapping to equivalent genes in the ELA319 genome bin, we found that genes from the second 11-gene set were moderately expressed (having a mean $\log_2$-fold expression relative to *dnaK* of 0.48 ± 0.16 and -0.21 ± 0.20 in Lakes 221 and Lake 304, respectively, across the 10 genes that could be linked between ELA319 and strain L227-S17). Additionally, we identified a loose gene cluster that included some of the genes required for a cbb_3 type terminal cytochrome *c* oxidase[121], detecting two copies of subunit I (*ccoN*; OZ401_001043 and OZ401_001052) and subunit II (*ccoO*; OZ401_001041 and OZ401_001045) but no clear homologs of subunits III and IV (*ccoP* and *ccoQ*). This gene cluster was not included in the ELA319 genome bin. It is possible that this gene cluster could encode a novel form of terminal oxidase, but the function of this putative complex is unclear. Overall, detection of two Complex I gene sets, along with a possible cbb_3-like complex, implies that strain L227-S17 might be metabolically flexible and able to change between multiple metabolic modes[122], although we have yet to identify growth conditions to induce these alternative metabolisms experimentally.

Supplementary Note 3

Diversity of the proposed "*Ca.* Chloroheliales" order. Searching the GTDB[46] (release 89) revealed two MAGs associated with the proposed "*Ca.* Chloroheliales" (i.e., "54-19)" order that were assembled

and binned in previous studies. One MAG, "*Chloroflexi* bin 54-19", was recovered from an ammonium sulfate bioreactor metagenome[123] and placed sister to the MAG of strain L227-5C that was recovered in this study (Fig. 3). The other MAG, "*Chloroflexi* RR_metagenome_bin16", was recovered from a polar surface soil metagenome (Robinson Ridge, Antarctica)[124] and placed basally to all other members of the "*Ca.* Chloroheliales" order (Fig. 3). Neither MAG contained detectable genes for chlorophototrophy (Fig. 3). Searching amino acid sequences predicted directly from the unassembled metagenome data used to generate these two bins, using the custom HMMs generated in this study (available in the code repository associated with this work), also did not reveal any RCI-related sequences other than those with a 100% match to chloroplast PscA (data not shown). These data suggest that not all members of the "*Ca.* Chloroheliales" are phototrophic.

In addition to searching the GTDB, we compared the two RCI-encoding MAGs of *Chloroflexota* members, recovered from Boreal Shield lake metagenomes, to the strains we enriched from Boreal Shield lake water. One of the MAGs, "Ca. Chloroheliaceae bin ELA729", had an average nucleotide identity (ANI) of 99.4% to the MAG of strain L227-5C and likely represents the same species. The second MAG, "Ca. Chlorohelix bin ELA319", had an ANI of 87.5% to the "Ca. Chx. allophototropha" L227-S17 genome and could represent a novel but related species. Our lack of recovery of an environmental MAG directly corresponding to strain L227-S17 could imply that strain L227-S17 was a low relative abundance community member in Boreal Shield lakes at the time of sampling. Related RCI-utilizing *Chloroflexota* species (e.g., represented by bin ELA319) may generally be higher in relative abundance compared to strain L227-S17 in Boreal Shield lake ecosystems.

Supplementary Methods

Enrichment of Geothrix *L227-G1*

The main contaminating bacterium in the L227-S17 culture after multiple rounds of cultivation in deep agar dilution series, *Geothrix* sp. L227-G1, was selectively enriched to characterize the strain. Biomass was picked from an agar tube of the L227-S17 culture (subculture generation 21) and was inoculated into liquid freshwater medium[28] amended with 10 mM sodium acetate and 100 mM poorly crystalline iron(III) oxide[125], kept at pH 6.5-7.0. Ferrous chloride (1 mM) was also added as a reducing agent. Cultures were incubated in the dark at 22°C. Due to slow growth on poorly crystalline iron(III) oxide, the ferric iron source was changed in later subcultures to 2.8 mM ferric nitrilotriacetic acid. Reduction of ferric iron to ferrous iron was confirmed using the ferrozine assay[60]. To avoid precipitates in the medium, fermentative conditions were promoted in the culture using 10 mM trisodium citrate, with 0.25 mM ferrous chloride as a reducing agent, prior to microscopy. Culture samples for microscopy were washed once with Fe- and citrate-free medium (via centrifugation at 12,000 x g, 2 min), and a dry mount was then prepared and stained with crystal violet. Light microscopy (phase contrast) was performed using an Axioplan2 imaging microscope system equipped with an Axiocam MRm camera (Carl Zeiss; Oberkochen, Germany). Microscopy images were acquired using the AxioVision software, version 4.6.3 SP1 (Carl Zeiss).

Selection against sulfate-reducing bacteria in enrichment cultures

Sulfate-reducing bacteria growing in enrichment cultures of L227-S17 were enriched in Sulfate Reducing Medium (M803; HiMedia Laboratories; Mumbai, Maharashtra, India). Inoculum from the L227-S17 culture was injected into Hungate tubes containing 10 mL of Sulfate Reducing Medium and a 90:10 N_2:CO_2 headspace, and tubes were incubated for seven days at room temperature in the dark. The enrichment was then subcultured into additional Hungate tubes containing Sulfate Reducing Medium that were spiked with sodium molybdate, a known inhibitor of sulfate reducing bacterial activity[126]. A molybdate concentration range of 0.2-200 mM was used, corresponding to a molybdate:sulfate ratio ranging from 1:100 to 10:1, given the 20 mM sulfate concentration in the medium. Tubes were incubated at room temperature in the dark for 14 days to monitor sulfate reducing bacterial activity, which was indicated by the development of black precipitate.

Biomass collection for spectroscopy

Picked colonies from the L227-S17 culture were centrifuged at 16-20,000 x g for 2 min at 4°C during each of three washes in phosphate buffered saline. During each wash, the golden portion of the pellet was selectively resuspended while discarding overlying agar and inorganic crystalline material that accumulated at the bottom of the pellet. A culture of *Chlorobium ferrooxidans* was grown in Chx3.1 liquid medium with 8 mM ferrous chloride (and no acetate), with medium pH adjusted to ~6.5, and was incubated at 22°C under white fluorescent light (60 µmol photons m^{-2} s^{-1}) until development of brown-coloured iron(III) oxides. Cells were then harvested and concentrated by centrifugation. Culture material was first centrifuged at 16,000 x g for 5 min at 4°C to collect a pellet containing cells mixed with abundant iron(III) oxides. This pellet was washed three times in phosphate-buffered saline, via centrifugation at 20,000 x g for 2 min at 4°C. After each wash step, the green layer that formed at the surface of the brown pellet was selectively resuspended for the subsequent wash, as described previously[103]. Lastly, a liquid culture of *Chloroflexus aurantiacus* J-10-fl was grown in PE medium[19] anaerobically under constant illumination (60 W Tungsten lamp, 30 cm illumination distance) at 50°C. *Chloroflexus aurantiacus* cells were kept cool during shipping to Hokkaido University and were then harvested by centrifugation at 15,000 x g for 5 min at 4°C. The resulting pellet was washed three times in 10 mM Tris-Cl (pH=8) via centrifugation at 12,000 x g for 2 min at 4°C. Cell pellets from the three cultures were either analyzed immediately after collection or frozen at -30°C for a maximum of eight weeks before spectroscopic analyses.

Identification of bacteriochlorophyll a

To identify bacteriochlorophyll *a* in HPLC samples, a reference culture of the purple nonsulfur bacterium *Rhodobacter capsulatus* SB1003[127] was grown in PYS medium[128] at 30°C under ~850 nm LED lights. Cells were harvested by centrifugation at 12,000 x g for 5 min and were then washed three times in phosphate-buffered saline via centrifugation at 12,000 x g for 2 min. Washed cells were kept frozen at -30°C for three days before analysis. Collected cell biomass was then analyzed using the same extraction and HPLC protocol as described in the Methods. At the same time as the *Rhodobacter capsulatus* sample, a sample of *Chloroflexus aurantiacus* culture, from the same culture as described in

the section above, was run to normalize retention time between the original HPLC run and the run for the bacteriochlorophyll *a* standard. Retention time was normalized by linear regression based on the three largest peaks at 667 nm in the HPLC data for *Chloroflexus aurantiacus* samples. Given that the slope of the regression was ~1 (1.002), retention time data were simply offset by the retention time difference (i.e., the y-intercept) calculated via regression. The *Chloroflexus aurantiacus* sample had been stored at -30°C for approximately 20 months before HPLC analysis with the bacteriochlorophyll *a* standard.

Electron microscopy

For TEM analysis, fixed cell pellets were washed, enrobed in 4% Noble agar (ThermoFisher Scientific; Waltham, Massachusetts, USA), and cut into 1 mm cubes. The cubes were fixed in 1% (w/v) osmium tetroxide for 45 min and subjected to a 25-100% ethanol dehydration series. Samples were then subjected to stepwise infiltration using 25-100% LR White Resin (Ted Pella; Redding, California, USA), transferred to gelatin capsules in 100% LR White Resin, and allowed to polymerize overnight at 60°C. An Ultracut UCT ultramicrotome (Leica Microsystems; Wetzlar, Germany) equipped with a diamond knife (DiATOME; Hatfield, Pennsylvania, USA) was used to cut 50 nm thin sections, which were then floated on 100-mesh copper grids and stained with 2% uranyl acetate and Reynold's lead citrate to enhance contrast. Sections were imaged under standard operating conditions using a Tecnai G2 F20 TEM (ThermoFisher Scientific; Waltham, Massachusetts, USA) that was running at 200 kV and was equipped with a Gatan 4k CCD camera (Gatan; Pleasanton, California, USA).

For SEM analysis, fixed cells were washed in phosphate-buffered saline and incubated in 1% (w/v) osmium tetroxide at room temperature for 30 min. Following incubation, cells were washed, deposited on an aluminum stub, dried, and then sputter coated with a gold:palladium mixture using a Desk V TSC Sample Preparation System (Denton Vacuum; Moorestown, New Jersey, USA). Prepared samples were imaged using a Quanta FEG 250 SEM (ThermoFisher Scientific) with a high-voltage setting of 10 kV and working distance of 9.9 mm.

Long read amplicon sequence analysis

For long read amplicon analysis, default settings were used for NanoCLUST (commit a09991c, fork: https://github.com/jmtsuji/nanoclust) for demultiplexed samples, except Medaka (Oxford Nanopore Technologies) was upgraded to version 1.5.0 to use the 'r941_min_sup_g507' model for polishing, and the minimum cluster threshold was decreased to 10 reads. Following NanoCLUST, PCR primers and adapters were trimmed off sequence clusters using CutAdapt version 3.4[78] in two rounds. In the first round, forward primers were removed, and the '--revcomp' flag was used to correct all sequences to the forward orientation. The reverse primer was removed in the second round. Any clusters without both forward and reverse primers were discarded. Trimmed sequences were then clustered at 99% identity[129] across all samples using the easy-cluster module (cov-mode 5, cluster-mode 2) of MMseqs 2 version 13.45111[130] to generate OTUs. Chimera filtration was performed on clustered sequences via UCHIME2 version 11.0.667 (32 bit), using the NCBI 16S rRNA gene database for Bacteria and Archaea type strains (December 2022) as the reference. The known 16S rRNA gene sequence of strain L227-S17

(based on genome sequencing) was added to the NCBI database to improve chimera detection accuracy. After chimera filtration, OTUs that corresponded to strain L227-S17 or strain L227-G1 were identified as described in the Methods. Any OTUs that did not match strain L227-S17 or strain L227-G1 were assigned genus-level taxonomy names according to NanoCLUST's taxonomic classifications, which were based on the NCBI 16S rRNA gene database for Bacteria and Archaea type strains (June 2021).

Read cloud metagenome sequencing

An enrichment culture of L227-S17 (subculture 15.2) was grown using white light (30 µmol photons m^{-2} s^{-1}; mix of incandescent and fluorescent sources) with 5 mM acetate and an additional 100 µM molybdate. High molecular weight genomic DNA was then extracted from picked colony material using a modified salting-out procedure (Manual CG000116, Rev A; 10x Genomics; Pleasanton, California, USA), followed by a phenol:chloroform treatment modified from Zhou and colleagues[131]. Briefly, colonies were picked from agar, centrifuged at 1010 x g for 7 min, and washed once with phosphate-buffered saline to remove excess agar. A lysis buffer consisting of 1.33 mL of 10 mM Tris-HCl (pH=8), 400 mM NaCl, and 2 mM ethylenediaminetetraacetic acid (EDTA; pH=8) was added to the pelleted biomass. In addition, 0.53 mL of 10% sodium dodecyl sulfate and 0.5 mL of 1 mg/mL proteinase K (ThermoFisher Scientific; Waltham, Massachusetts, USA), 1% SDS, and 2 mM EDTA (pH=8) were added. Cells were then gently lysed for 18 hours at 37°C. Following lysis, 4.3 mL of 5 M NaCl was added, and the sample was centrifuged at 1010 x g for 5 min. The supernatant was transferred to 15 mL MaXtract High Density tubes (Qiagen; Venlo, The Netherlands), gently mixed with 1 volume phenol:chloroform:isoamyl alcohol (25:24:1), and centrifuged at 1500 x g for 2 min. The resulting 11 mL of supernatant was transferred into 19.5 mL of ice-cold 100% ethanol, mixed by inversion, and aliquoted into 1.5 mL tubes for ethanol precipitation. Tubes were centrifuged at 4°C and 6200 x g for 5 min to precipitate DNA. After removal of supernatant and drying of DNA pellets, the dry DNA pellets were serially re-suspended into a single 30 µL aliquot of Tris-EDTA (TE; 10 mM Tris-HCl and 1 mM EDTA; pH=8) buffer.

Size selection was performed on the resulting extract using pulsed-field gel electrophoresis and electroelution, as described previously[132]. Briefly, pulsed-field gel electrophoresis was performed using a CHEF MAPPER Pulsed-Field Gel Electrophoresis system (Bio-Rad; Hercules, California, USA) run at 14°C, 5.5 V/cm, 1.0-6.0 s pulse, and 120° angle, for 16 hours. Electroelution of DNA from the excised gel fragment, targeting DNA strands longer than 25 kb, was performed at 120 V for 2 h in cellulose membrane dialysis tubing (12,000 MWCO with 33 mm average flat width; Sigma-Aldrich; St. Louis, Missouri, USA), with current reversed for 1 min before completion. Dialysis tubing was prepared in advance of electroelution by boiling in a 2% sodium bicarbonate and 1 mM EDTA (pH=8) solution for 10 min, then boiling in water for 10 min, and finally storing in 20% ethanol and 1 mM EDTA (pH=8) at 4°C until use. Electroeluted DNA solution was concentrated using Amicon Ultra-15 Centrifugal Filter Units (30 kDa MWCO; Millipore; Burlington, Massachusetts, USA), and DNA was washed via ethanol precipitation and resuspended in 20 µL of TE buffer.

Library preparation for read cloud DNA sequencing was performed on the extracted DNA using the TELL-Seq WGS Library Prep Kit[133] (Universal Sequencing Technology; Canton, Massachusetts, USA) following ultralow input protocol recommendations for small genomes. Amplification of the library was performed using 10 µL TELL-beads and 16 amplification cycles. The library was sequenced using a MiSeq Reagent Kit v2 (300-cycle; Illumina) with 2x150 bp read length on a MiSeq System (using MiSeq control software, version 2.5.0.5) to a depth of 19.6 million total reads. Following sequencing, Picard version 2.21.6 (Picard Toolkit – Broad Institute – http://broadinstitute.github.io/picard/) was used to demultiplex output reads.

Culture metagenome analysis

Short read metagenome data generated for enrichment cultures of strain L227-S17 and strain L227-5C were assembled and binned into MAGs. For a metagenome of the early L227-5C enrichment (subculture 0), the ATLAS pipeline, version 2.2.0[80], was used for read quality control, metagenome assembly, and genome binning. A genome bin corresponding to strain L227-5C (based on classification via the GTDB-Tk[104], version 0.3.3, to the "54-19" order of the *Chloroflexota* phylum) was then manually curated (see next section) and was annotated using PGAP[82] during submission to the NCBI database. A metagenome of the early L227-S17 enrichment (subculture 1) and a read cloud metagenome of a later enrichment culture of L227-S17 (subculture 15.2; described above) were also analyzed using ATLAS version 2.2.0. Read quality control and metagenome assembly were performed normally for the early enrichment (subculture 1) metagenome, but custom steps were needed to utilize the read cloud metagenome data (for subculture 15.2). Read quality control and metagenome assembly steps were first performed normally within the ATLAS pipeline for the read cloud metagenome. Then, to achieve a more contiguous assembly, quality control and assembly were performed again outside of ATLAS. The Tell-Read pipeline, version 0.9.7 (Universal Sequencing Technology), was used to re-analyze raw read outputs from the sequencer, performing demultiplexing and quality control on index reads via default settings, and the reads were then assembled using Tell-Link, version 1.0.0 (Universal Sequencing Technology), using global and local kmer lengths of 65 and 35, respectively. Following assembly using Tell-Link, the assembled scaffolds initially generated by ATLAS for the read cloud metagenome were substituted with the scaffolds generated using Tell-Link, and the ATLAS pipeline was then allowed to continue normally into the differential abundance genome binning step, which used MaxBin 2 version 2.2.4[101] and MetaBAT2 version 2.12.1[102]. (The same genome binning tools were used for analysis of the L227-5C sample.) All ATLAS settings for the analyses of the L227-5C and L227-S17 cultures are available in the code repository associated with this work.

Manual genome bin curation

To manually curate the genome bin of strain L227-5C, which was highly fragmented, all predicted protein sequences in the bin were taxonomically classified using the Kaiju web server[134] (accessed February 2020), via the proGenomes database (May 16th, 2017 version), with default settings. Taxonomic lineages of hits were determined from taxonomy IDs by the script *make-lineage-csv.py* (https://github.com/dib-lab/2018-ncbi-lineages), commit 63e8dc7, using mapping (taxdump) information from the NCBI taxonomy database (October 2019)[135]. Any contigs having at least one hit

classified within the *Chloroflexota* phylum were retained, but other contigs were further screened for possible mis-binning. Protein-coding sequences on other contigs were searched against the NCBI RefSeq protein database (October 2019)[35] using BLASTP[95] version 2.9.0 (e-value cutoff = 10^{-10}), and taxonomic lineages of hits were determined as above. Any contigs having a BLASTP hit with >80% identity to a RefSeq entry were manually screened at a gene-by-gene level for signs of mis-binning. Contigs containing a tRNA or rRNA gene, identified using Prokka version 1.14.6[136], were similarly manually screened. Identified tRNA and rRNA genes were searched against the NCBI nr database (excluding uncultured subjects) via BLASTN using the NCBI BLAST web server (accessed March 2020)[137] to check their taxonomic affiliation. In addition, contigs with median coverage values greater than or less than one standard deviation of the mean contig coverage value across the genome were assessed for signs of mis-binning via gene-by-gene manual curation. Potentially mis-binned contigs (e.g., based on having common and non-*Chloroflexota* taxonomic classifications across multiple genes) were removed from the genome bin.

In addition, although we eventually closed the genome of strain L227-S17, we curated an earlier genome bin of strain L227-S17, which was recovered from read cloud metagenome sequencing data, for preliminary study of the strain. To manually curate the genome bin, genes predicted by Prodigal version 2.6.3[138] were queried against the NCBI RefSeq protein database (October 2019)[35] using BLASTP[95] version 2.9.0 (e-value cutoff = 10^{-10}), and the taxonomic lineages of hits were determined as for strain L227-5C above. Any scaffolds with at least one hit to a subject sequence associated with the phylum *Chloroflexota* were kept, and the top five highest-scoring hits for each gene were considered. Remaining scaffolds were checked for their median coverage, length, and gene classifications, and genes were further assessed against the NCBI nr protein database via BLASTP (on the NCBI BLAST web server[137]; February 2020). Short contigs containing no genes or genes consistently matching the same non-*Chloroflexota* phylum were discarded from the bin. This curated MAG is available as NCBI accession GCA_013390565.1 but was not used for the genomic analyses in this work.

Hybrid genome assembly

We developed a custom pipeline named Rotary (https://github.com/rotary-genomics/rotary, doi:10.5281/zenodo.6951912) for assembly of hybrid long- and short-read genome sequencing data. Commit e636236 was used to assemble a closed genome of strain L227-S17. Within Rotary, long read QC was performed using BBMap version 37.99 (Bushnell B. – sourceforge.net/projects/bbmap/) to remove reads shorter than 1 kb or with quality score <13. The long reads were then assembled using Flye version 2.9-b1768 with the '--nano-hq' input flag in 'meta' mode[139]. Potential short gaps or overlaps on the ends of circular contigs were corrected using a customized wrapper of the 'merge' module of Circlator version 1.5.5[140]. Assembled contigs were then polished using Medaka 1.4.4 (Oxford Nanopore Technologies) with the 'r941_min_sup_g507' model, and short reads were mapped to polished contigs using BWA-MEM version 0.7.17[141]. Short read polishing was then performed on contigs using Polypolish version 0.5.0[142], followed by a second round of short read polishing via POLCA version 4.0.8[143]. After short read polishing, only contigs with a short read coverage depth of >10x were retained for downstream analysis. Circular contigs were then rotated using the 'fixstart' module of Circlator version 1.5.5. The *dnaA* gene, identified using the HMM 'Bac_DnaA'

(PF00308.21, Pfam[86]) via hmmsearch version 3.3.2[85], was set as the start point of the applicable contigs, based on coding sequence predictions from Prodigal version 2.6.3[138]. A final round of short read polishing, using Polypolish as above, was then performed on rotated circular contigs. Rotary was also used to assemble a closed genome bin of *Geothrix* sp. L227-G1 as described below.

Geothrix *L227-G1 genome analysis*

Read cloud sequencing data from a L227-S17 enrichment culture, subculture 15.2 (described above) was combined with long read sequencing of a second L227-S17 enrichment culture, subculture 15.c, to recover a closed genome bin of the partner strain *Geothrix* sp. L227-G1. For subculture 15.c, cells were grown using 0.5-0.6% (w/v) agar, with the same light source and additional molybdate as subculture 15.2 (i.e., 30 µmol photons m^{-2} s^{-1} white light, mix of incandescent and fluorescent sources; 100 µM molybdate), and were harvested via centrifugation of the whole agar slurry at 20,000 x g for ≥30 min at 4°C. The resulting pellets were then used for a total of eight DNA extractions using the DNeasy UltraClean Microbial Kit (Qiagen), and output DNA was concentrated using the DNA Clean and Concentrator-5 kit (Zymo Research). A DNA sequencing library was then prepared via the Ligation Sequencing Kit (SQK-LSK110; Oxford Nanopore Technologies) with Long Fragment Buffer, using 370 ng of the concentrated DNA, combined with a spike-in of 125 ng of Lambda DNA (EXP-CTL001; Oxford Nanopore Technologies), as input. The library was sequenced using a R9.4.1 Flongle flow cell (FLO-FLG001; Oxford Nanopore Technologies). Adaptive sampling was used to deplete reads matching the Lambda phage genome (NC_001416.1) during sequencing via MinKNOW version 21.02.1 (Oxford Nanopore Technologies). Basecalling was performed using Guppy 5.0.16 (Oxford Nanopore Technologies) with the Super Accuracy model, generating 0.23 million reads with a mean length of 3.9 kb.

We performed quality control of short read (read cloud) data from subculture 15.2 via ATLAS 2.2.0, using reads demultiplexed with Picard as input. The long read data from subculture 15.c and this QC-processed short read data were then subjected to hybrid sequence assembly, using Rotary (commit fd5acee) as described for strain L227-S17, to generate a set of assembled and polished contigs. We then mapped the QC-processed short read data to the contigs using BBMap version 37.99 (Bushnell B.), using a minimum sequence identity threshold of 95%, and performed genome binning using MetaBAT2 version 2.15[102]. This analysis resulted in a single-contig, closed and circular genome bin that had 16S rRNA gene sequences with >99% match to 16S rRNA gene amplicon sequences of *Geothrix* sp. L227-G1. All other contigs in the assembly were associated with strain L227-S17, except an 8.1 kb linear contig that had 10-fold lower coverage depth than any chromosomal sequence. We annotated the closed circular genome bin using PGAP version 2022-04-14.build6021[82].

Metagenome-based community comparisons

To compare the microbial community composition of enrichment cultures across all analyzed samples, we clustered MAGs and genomes constructed from the L227-5C metagenome (subculture 0), L227-S17 short read metagenomes (subcultures 1 and 15.2), and the closed genome assemblies of strain L227-S17 and *Geothrix* sp. L227-G1, using FastANI version 1.33[144] with a clustering threshold of

97.5%. When more than one genome belonged to a cluster, which only occurred in the case of strain L227-S17 and *Geothrix* sp. L227-G1, we used the closed circular genome version as the cluster representative. All short read metagenome data were processed using the 'qc' module of ATLAS 2.8.2[80]. We then mapped the QC-processed short-read metagenome data to the clustered genome set using BBMap version 37.99 (Bushnell B.), using a minimum percent identity threshold of 90% and the 'ambiguous=best' setting. Similarly, we mapped all QC-processed long-read metagenome data (QC performed using Rotary, above) to the clustered genome set using Minimap2 version 2.23[145], and we excluded secondary or supplementary alignments using samtools 1.15[146]. Relative abundances were calculated based on the percent recruitment of reads to genomes.

Collection of reference genomes/genes

To compare the genomic properties of *Chloroflexota* phylum members, representative genomes associated with the phylum were downloaded from NCBI based on information in GTDB[46] release 89. All genomes that represented a type species within the *Chloroflexota* according to NCBI and the GTDB were downloaded, as well as genomes representing members of known phototrophic clades (i.e., the *Chloroflexaceae* family[23], the "*Ca.* Thermofonsia" order[24], and the "*Ca.* Roseiliniales" order[147]). Selected genomes of known phototrophs that were deposited in other genome databases were also downloaded (i.e., genomes of "*Ca.* Chloranaerofilum corporosum"[147], "*Ca.* Roseilinea gracile"[147], and "*Ca*. Chlorothrix halophila"[21]; see details in the code repository associated with this work). In addition, any genome bins listed in the GTDB that belonged to the "54-19" order (which represented the name of the "*Ca.* Chloroheliales" order in this database) were downloaded. Non-phototrophic lineages were subsequently pruned to one representative per genus, except for non-phototrophic lineages closely related to phototrophic clades. In total, this left 58 genomes, including genomes of 28 known phototrophs. This genome collection was used for subsequent creation of a *Chloroflexota* species tree.

We collected additional genomes to compare photosynthesis genes encoded by "*Ca.* Chloroheliales" members to genes of other phototrophs. Genomes containing homologs of the genes of interest (*pscA*/*pshA*/*psaAB*, *fmoA*, *csmA*, *bchIDH*/*chlIDH*, *bchLNB*/*chlLNB*, *bchXYZ*, and/or *rbcL*; see Methods) were determined using a combination of automated detection via the AnnoTree web server[148] (relying on GTDB release 89) and descriptions in the literature. Representative genomes from this initial genome set were selected from the GTDB, based on genome quality and taxonomic novelty, before being downloaded from NCBI. Where needed, genome nucleotide files were annotated using Prodigal 2.6.3[138]. Potential orthologs of interest were then identified in the downloaded genomes using bidirectional BLASTP, which was performed using the primary sequences of known reference genes as queries (shown in Supplementary Data 2) via BackBLAST[96] version 2.0.0-alpha3. In addition, for some sequence sets (i.e., Type I reaction centers; Bch proteins; RbcL), additional reference sequences were added manually based on literature references[4,97,99]. Sequence sets were then used to build photosynthesis gene phylogenies.

Environmental RCI-associated gene search

Preparation of the collection of *Chloroflexota*-associated genomes revealed that two existing genomes bins in the GTDB placed taxonomically within the "*Ca.* Chloroheliales" order. Unassembled read files for the environmental metagenomes associated with those bins were downloaded from the European Nucleotide Archive (ENA) to identify any potentially unbinned but novel photosynthesis-associated genes. Short protein sequences were predicted directly from unassembled read data using FragGeneScanPlusPlus[105] commit 9a203d8. These short protein sequences were scanned using the two custom HMMs developed in this study (above) via hmmsearch[85] v3.1b2 using a relaxed e-value cutoff of 10^{-1}. The average coverage and read recruitment of each genome bin was also calculated by mapping the unassembled metagenomic reads onto the bins using bbmap.sh version 38.75 (BBMap – Bushnell B.).

Electron transport gene identification

Genes potentially associated with photosynthetic and non-photosynthetic electron transport were identified in the strain L227-S17 genome using both sequence and structural homology approaches. Iron-sulfur cluster binding motifs were identified in the predicted proteins of strain L227-S17 using the ScanProsite tool[149] with PROSITE release 2023_04[150]. Candidate genes were then identified by both homology to reference sequences of known phototrophs and by checking their motif composition. After initial screening, the tertiary structures associated with candidate genes were predicted using ColabFold version 1.5.2-patch[151,152] with default settings. These structural predictions were searched using the FoldSeek web server, release 8-ef4e960[153], against default structural databases, with particular attention given to hits of reference structures in the Protein Data Bank[154]. Structural alignments to key entries were manually compared using UCSF ChimeraX version 1.3[155]. Where relevant, transmembrane regions of candidate genes were predicted using DeepTMHMM release 1.0.24[156]. As an exception to the above workflow, putative genes involved in Complex III could be identified solely using sequence homology, and putative genes involved in Complex I were identified using gene annotation data from the PGAP[82].

Reciprocal best hit protein searches, via the easy-rbh module of MMseqs version 13.45111[130], were carried out against the predicted proteins of bin ELA319 using all predicted proteins of strain L227-S17 as a query. Default settings were used, except that a minimum coverage threshold of 80% was set. Hits were manually curated based on contiguous neighbouring genes, percent identity, and query coverage to create a one-to-one mapping of genes from strain L227-S17 to bin ELA319. Using this gene mapping information, we checked the gene expression profiles of candidate electron transfer genes in Lake 221 and Lake 304 metatranscriptomes to see if these genes were actively expressed.

(Bacterio)chlorophyll synthase phylogeny

Manually curated protein sequences of chlorophyll *a* synthase, bacteriochlorophyll *a* synthase, and bacteriochlorophyll *g* synthase were selected from known phototrophs. These sequences were compared to the bacteriochlorophyll *a* synthase (BchG) and possible chlorophyll *a* synthase (ChlG)

sequences identified from the strain L227-S17 and strain L227-5C genomes. Predicted primary sequences were aligned via Clustal Omega[89] version 1.2.4, and the resulting alignment was then masked using Gblocks[100] version 0.91b with relaxed settings (-t=p -b3=40 -b4=4 -b5=h). The alignment length after making was 276 residues. A maximum likelihood protein phylogeny was built using the masked alignment via IQ-TREE[91] version 2.2.0.3 with 1000 ultrafast bootstraps[93]. ModelFinder[92] was used to select LG+F+G4 as the evolutionary rate model. The resulting unrooted phylogeny was visualized in Dendroscope version 3.8.3[157]. We confirmed that none of the input sequences grouped with bacteriochlorophyll *c* synthases (BchK) by making a similar phylogeny that also included BchK references.

Lake sampling

We sampled the water columns of eight seasonally anoxic lakes (Lakes 221, 222, 224, 227, 304, 373, 442, and 626) within the IISD-ELA, along with a permanently oxic reference lake (Lake 239), for DNA and/or RNA across four main sampling events. Sampling in June 2016, September 2016, and September 2017 involved multi-depth hypolimnion profiling of Lakes 227 and 442, for which metagenome data have been reported previously[103], along with mid-hypolimnion sampling of other selected lakes. A full water column profile of DNA samples was collected for Lake 227 in September 2017, along with collection of RNA samples from a single depth of Lake 227 in the upper anoxic hypolimnion. Lastly, in July 2018, Lakes 221 and 304 were surveyed again. A single depth in the mid anoxic hypolimnion of both lakes was sampled for both DNA and RNA.

During lake water column sampling, temperature and dissolved oxygen were measured using an EXO multi-parameter sonde (Xylem; Rye Brook, New York, U.S.A.), although for selected sampling events a HQ40D Portable Multi Meter for Water (Hach; Loveland, Colorado, U.S.A.) or handheld water quality meter (Xylem) were used. Light intensity was measured using a LI-192 underwater quantum sensor (LI-COR Biosciences; Lincoln, New England, U.S.A.). Total dissolved iron was measured using the ferrozine assay[60,158] on water samples that were filtered in-line (via 0.45 µm membrane filters) and preserved in 0.5 N HCl, and sulfate and dissolved organic carbon samples were collected and measured as described previously[42].

Environmental DNA/RNA extraction

For each Sterivex filter used for DNA collection, the Sterivex filter case was opened, and the filter membrane was carefully removed using a flame-sterilized scalpel blade. Each filter membrane was cut in half lengthwise along the filter. One half was stored frozen to provide a backup sample, and DNA was extracted from the other half using the DNeasy PowerSoil or DNeasy PowerSoil HTP 96 Kit (Qiagen; Venlo, The Netherlands). Extractions were performed according to the kit protocol, and the optional 10 min incubation at 70°C after adding Solution C1 was performed to enhance cell lysis. Mechanical lysis was performed for samples in PowerBead Tubes using a FastPrep-24 instrument (MP Biomedicals; Santa Ana, California, U.S.A.) set at 5 m/s for 45 s, and mechanical lysis was performed for samples in PowerBead DNA Plates using a mixer mill MM 400 (Retsch; Haan, Germany) set at 30 Hz for 10 min. Resulting DNA concentrations were then quantified using a Nanodrop

spectrophotometer (Thermo Fisher Scientific) or using the Qubit dsDNA HS Assay Kit with Qubit 2.0 fluorometer (Thermo Fisher Scientific).

RNA extraction was performed using the ZymoBIOMICS DNA/RNA Miniprep Kit (Zymo Research) with initial steps of the protocol modified slightly to accommodate the volume of DNA/RNA Shield associated with each filter. Sterivex filters filled with DNA/RNA Shield were thawed, and the DNA/RNA Shield was pumped out of the filter cases and saved for downstream use. Filter cases were opened and filters excised as described above. Each filter half was cut into small pieces. One half was stored in a clean 2 mL microfuge tube along with half of the collected DNA/RNA Shield solution and frozen as a backup sample. The other filter half, along with the remaining DNA/RNA Shield, was added into a dry ZR BashingBead Lysis Tube for extraction. Mechanical lysis was performed using a FastPrep-24 instrument (MP Biomedicals). Lysis tubes were shaken at 6.5 m/s for 60 s twice, and tubes were allowed to cool on ice for at least 60 s between mechanical lysis rounds. After centrifugation, the entire supernatant volume was transferred to a new microfuge tube, and one volume of DNA/RNA Lysis Buffer was added to the tube and mixed. RNA extraction was then performed as described in the Methods. Only RNA (and not DNA) extracts were saved because corresponding Sterivex filters for DNA were collected and processed for the same lake depths using the protocol described above. The resulting RNA extracts were quantified using a Nanodrop spectrophotometer (Thermo Fisher Scientific) and Qubit RNA Assay Kit with Qubit 2.0 fluorometer (Thermo Fisher Scientific). Extracts were also run on a 1% agarose gel stained with GelRed (Biotium; Fremont, California, U.S.A.) to confirm that rRNA of the expected lengths was visible.

Gene expression calculations

To calculate gene expression levels based on environmental metatranscriptome data, the single-copy taxonomic marker gene *dnaK* was identified in each RCI-encoding *Chloroflexota* MAG that was recovered from Boreal Shield lake metagenome data. Homologs of *dnaK* were identified based on eggNOG-mapper[159] annotations (version 1.0.3), and annotations were confirmed using a BLASTP[95] search of the predicted protein sequence against the RefSeq[35] protein database (BLAST version 2.9.0; October 2019 RefSeq version). In the case of bin ELA729, two genes were annotated as *dnaK*, but one of the genes had low predicted sequence identity at the amino acid level to DnaK proteins in RefSeq (~34% identity) and most closely matched DnaK encoded by members of *Chitinophaga* spp. in the *Bacteroidetes* phylum, so this gene was not used for normalization. Relative expression levels of each gene within a MAG were then calculated by dividing the gene length-normalized hit count of each gene by the gene-length normalized hit count of *dnaK*, and expression levels were averaged among replicate metatranscriptomes. Calculations relied on pandas version 1.2.3[160]. Functional annotations of genes with high relative expression were determined based on the results of eggNOG-mapper version 1.0.3, which was run as part of the ATLAS ‘Genecatalog’ module on environmental metagenome data, combined with manual annotation of photosynthesis-associated genes.

Geospatial data processing

A map of lakes at the IISD-ELA was generated using major and minor water body information from the CanMap Water dataset (DMTI Spatial; Markham, Canada)[51]. Topographical data from a digital elevation model (30 m intervals; DMTI Spatial)[52] was displayed as 20 m contour lines on the map. (Discussion of map data reflects the views of the authors and not of DMTI Spatial.) In addition, the approximate range of Boreal Shield regions on Earth was determined using a subset of the Global GIS geospatial data collection (Esri; Redlands, U.S.A.)[53]. Regions defined as the major habitat "Boreal forest/taigas" (within the World Wildlife Fund Ecoregions dataset; wwf_eco.shp)[54], were intersected with geologic provinces that included the term "Shield" in their entry name (within the Geologic Provinces of the World dataset, United States Geological Survey; wep_prvg.shp). Intersection calculations were performed using ArcGIS version 10.3.1.4959 (Esri). The resulting data were visualized on a Natural Earth basemap consisting of 1:50 m land, 1:50 m ocean, and 1:110 m lake vectors (naturalearthdata.com). All map visualizations were performed using QGIS versions 2.14.0 and 3.6.3 (QGIS Association).

108. Tank, M. & Bryant, D. A. Nutrient requirements and growth physiology of the photoheterotrophic Acidobacterium, *Chloracidobacterium thermophilum*. *Front Microbiol* **6**, 226 (2015).
109. Jiao, Y., Kappler, A., Croal, L. R. & Newman, D. K. Isolation and characterization of a genetically tractable photoautotrophic Fe(II)-oxidizing bacterium, *Rhodopseudomonas palustris* strain TIE-1. *Appl Environ Microbiol* **71**, 4487–4496 (2005).
110. Overmann, J. & Garcia-Pichel, F. The phototrophic way of life. in *The Prokaryotes: Prokaryotic Communities and Ecophysiology* (eds. Rosenberg, E., DeLong, E. F., Lory, S., Stackebrandt, E. & Thompson, F.) 203–257 (Springer Berlin Heidelberg, 2013). doi:10.1007/978-3-642-30123-0_51.
111. Imhoff, J. F. The family *Chlorobiaceae*. in *The Prokaryotes* (eds. Rosenberg, E., DeLong, E. F., Lory, S., Stackebrandt, E. & Thompson, F.) 501–514 (Springer Berlin Heidelberg, 2014). doi:10.1007/978-3-642-38954-2_142.
112. Gich, F., Garcia-Gil, J. & Overmann, J. Previously unknown and phylogenetically diverse members of the green nonsulfur bacteria are indigenous to freshwater lakes. *Arch Microbiol* **177**, 1–10 (2001).
113. Orf, G. S. *et al.* Evidence for a cysteine-mediated mechanism of excitation energy regulation in a photosynthetic antenna complex. *Proc Natl Acad Sci USA* **113**, E4486–E4493 (2016).
114. Schütz, M. *et al.* Early evolution of cytochrome *bc* complexes. *J Mol Biol* **300**, 663–675 (2000).
115. Sattley, W. M. *et al.* The genome of *Heliobacterium modesticaldum*, a phototrophic representative of the *Firmicutes* containing the simplest photosynthetic apparatus. *J Bacteriol* **190**, 4687–4696 (2008).
116. Xiong, J., Inoue, K. & Bauer, C. E. Tracking molecular evolution of photosynthesis by characterization of a major photosynthesis gene cluster from *Heliobacillus mobilis*. *Proc Natl Acad Sci USA* **95**, 14851–14856 (1998).
117. Dong, S. *et al.* Structure of the Acidobacteria homodimeric reaction center bound with cytochrome *c*. *Nat Commun* **13**, 7745 (2022).
118. Chen, J.-H. *et al.* Architecture of the photosynthetic complex from a green sulfur bacterium. *Science* **370**, (2020).

119. Romberger, S. P. & Golbeck, J. H. The bound iron-sulfur clusters of type-I homodimeric reaction centers. *Photosynth Res* **104**, 333–346 (2010).
120. Muraki, N., Seo, D., Shiba, T., Sakurai, T. & Kurisu, G. Asymmetric dimeric structure of ferredoxin-NAD(P)$^+$ oxidoreductase from the green sulfur bacterium *Chlorobaculum tepidum*: implications for binding ferredoxin and NADP$^+$. *J Mol Biol* **401**, 403–414 (2010).
121. Pitcher, R. S. & Watmough, N. J. The bacterial cytochrome cbb_3 oxidases. *Biochim Biophys Acta, Bioenerg* **1655**, 388–399 (2004).
122. Spero, M. A. *et al.* Different functions of phylogenetically distinct bacterial complex I isozymes. *J Bacteriol* **198**, 1268–1280 (2016).
123. Kantor, R. S. *et al.* Bioreactor microbial ecosystems for thiocyanate and cyanide degradation unravelled with genome-resolved metagenomics. *Environ Microbiol* **17**, 4929–4941 (2015).
124. Ji, M. *et al.* Atmospheric trace gases support primary production in Antarctic desert surface soil. *Nature* **552**, 400–403 (2017).
125. Umezawa, K., Kojima, H., Kato, Y. & Fukui, M. Disproportionation of inorganic sulfur compounds by a novel autotrophic bacterium belonging to *Nitrospirota*. *Syst Appl Microbiol* **43**, 126110 (2020).
126. Biswas, K. C., Woodards, N. A., Xu, H. & Barton, L. L. Reduction of molybdate by sulfate-reducing bacteria. *Biometals* **22**, 131–139 (2009).
127. Strnad, H. *et al.* Complete genome sequence of the photosynthetic purple nonsulfur bacterium *Rhodobacter capsulatus* SB 1003. *J Bacteriol* **192**, 3545–3546 (2010).
128. Nagashima, K. V. P., Hiraishi, A., Shimada, K. & Matsuura, K. Horizontal transfer of genes coding for the photosynthetic reaction centers of purple bacteria. *J Mol Evol* **45**, 131–136 (1997).
129. Edgar, R. C. Updating the 97% identity threshold for 16S ribosomal RNA OTUs. *Bioinformatics* **34**, 2371–2375 (2018).
130. Steinegger, M. & Söding, J. MMseqs2 enables sensitive protein sequence searching for the analysis of massive data sets. *Nat Biotechnol* **35**, 1026–1028 (2017).
131. Zhou, J., Bruns, M. A. & Tiedje, J. M. DNA recovery from soils of diverse composition. *Appl Environ Microbiol* **62**, 316–322 (1996).
132. Cheng, J., Pinnell, L., Engel, K., Neufeld, J. D. & Charles, T. C. Versatile broad-host-range cosmids for construction of high quality metagenomic libraries. *J Microbiol Methods* **99**, 27–34 (2014).
133. Chen, Z. *et al.* Ultra-low input single tube linked-read library method enables short-read second-generation sequencing systems to generate highly accurate and economical long-range sequencing information routinely. *Genome Res* **30**, 898–909 (2020).
134. Menzel, P., Ng, K. L. & Krogh, A. Fast and sensitive taxonomic classification for metagenomics with Kaiju. *Nat Commun* **7**, 11257 (2016).
135. Schoch, C. L. *et al.* NCBI Taxonomy: a comprehensive update on curation, resources and tools. *Database* **2020**, baaa062 (2020).
136. Seemann, T. Prokka: rapid prokaryotic genome annotation. *Bioinformatics* **30**, 2068–2069 (2014).
137. Boratyn, G. M. *et al.* BLAST: a more efficient report with usability improvements. *Nucl Acids Res* **41**, W29–W33 (2013).
138. Hyatt, D. *et al.* Prodigal: prokaryotic gene recognition and translation initiation site identification. *BMC Bioinform* **11**, 1–11 (2010).
139. Kolmogorov, M. *et al.* metaFlye: scalable long-read metagenome assembly using repeat graphs. *Nat Methods* **17**, 1103–1110 (2020).
140. Hunt, M. *et al.* Circlator: automated circularization of genome assemblies using long sequencing reads. *Genome Biol* **16**, 294 (2015).

141. Li, H. Aligning sequence reads, clone sequences and assembly contigs with BWA-MEM. *arXiv* 1303.3997 (2013) doi:10.48550/arXiv.1303.3997.
142. Wick, R. R. & Holt, K. E. Polypolish: Short-read polishing of long-read bacterial genome assemblies. *PLOS Comput Biol* **18**, e1009802 (2022).
143. Zimin, A. V. & Salzberg, S. L. The genome polishing tool POLCA makes fast and accurate corrections in genome assemblies. *PLOS Comput Biol* **16**, e1007981 (2020).
144. Jain, C., Rodriguez-R, L. M., Phillippy, A. M., Konstantinidis, K. T. & Aluru, S. High throughput ANI analysis of 90K prokaryotic genomes reveals clear species boundaries. *Nat Commun* **9**, 5114 (2018).
145. Li, H. Minimap2: pairwise alignment for nucleotide sequences. *Bioinformatics* **34**, 3094–3100 (2018).
146. Danecek, P. *et al.* Twelve years of SAMtools and BCFtools. *GigaScience* **10**, giab008 (2021).
147. Tank, M., Thiel, V., Ward, D. M. & Bryant, D. A. A panoply of phototrophs: an overview of the thermophilic chlorophototrophs of the microbial mats of alkaline siliceous hot springs in Yellowstone National Park, WY, USA. in *Modern Topics in the Phototrophic Prokaryotes: Environmental and Applied Aspects* (ed. Hallenbeck, P. C.) 87–137 (Springer International Publishing Switzerland, 2017). doi:10.1007/978-3-319-46261-5_3.
148. Mendler, K. *et al.* AnnoTree: visualization and exploration of a functionally annotated microbial tree of life. *Nucleic Acids Res* **47**, 4442–4448 (2019).
149. de Castro, E. *et al.* ScanProsite: detection of PROSITE signature matches and ProRule-associated functional and structural residues in proteins. *Nucl Acids Res* **34**, W362–W365 (2006).
150. Sigrist, C. J. A. *et al.* New and continuing developments at PROSITE. *Nucleic Acids Research* **41**, D344–D347 (2013).
151. Mirdita, M. *et al.* ColabFold: making protein folding accessible to all. *Nat Methods* **19**, 679–682 (2022).
152. Jumper, J. *et al.* Highly accurate protein structure prediction with AlphaFold. *Nature* **596**, 583–589 (2021).
153. van Kempen, M. *et al.* Fast and accurate protein structure search with Foldseek. *Nat Biotechnol* **42**, 243-246 (2023).
154. Berman, H. M. *et al.* The Protein Data Bank. *Nucl Acids Res* **28**, 235–242 (2000).
155. Pettersen, E. F. *et al.* UCSF ChimeraX: structure visualization for researchers, educators, and developers. *Protein Sci* **30**, 70–82 (2021).
156. Hallgren, J. *et al.* DeepTMHMM predicts alpha and beta transmembrane proteins using deep neural networks. *bioRxiv* 2022.04.08.487609 (2022) doi:10.1101/2022.04.08.487609.
157. Huson, D. H. & Scornavacca, C. Dendroscope 3: An interactive tool for rooted phylogenetic trees and networks. *Syst Biol* **61**, 1061–1067 (2012).
158. Viollier, E., Inglett, P. W., Hunter, K., Roychoudhury, A. N. & Van Cappellen, P. The ferrozine method revisited: Fe(II)/Fe(III) determination in natural waters. *Appl Geochem* **15**, 785–790 (2000).
159. Huerta-Cepas, J. *et al.* Fast genome-wide functional annotation through orthology assignment by eggNOG-mapper. *Mol Biol Evol* **34**, 2115–2122 (2017).
160. McKinney, W. Data structures for statistical computing in python. in *Proceedings of the 9th Python in Science Conference* vol. 445 51–56 (Austin, TX, 2010).
