# Supplementary material for: Anoxygenic phototroph of the Chloroflexota uses a type I reaction centre
Source: Nature. 2024 Mar 13;627(8005):915–22. doi: 10.1038/s41586-024-07180-y (PMC10972752; doi:10.1038/s41586-024-07180-y)
Supplement: Supplementary file 2 — Reporting Summary [file 41586_2024_7180_MOESM2_ESM.pdf]

nature portfolio

Corresponding author(s): Jackson M. Tsuji; Josh D. Neufeld

Last updated by author(s): Jan 19, 2024

Reporting Summary

Nature Portfolio wishes to improve the reproducibility of the work that we publish. This form provides structure for consistency and transparency in reporting. For further information on Nature Portfolio policies, see our Editorial Policies and the Editorial Policy Checklist.

Statistics

For all statistical analyses, confirm that the following items are present in the figure legend, table legend, main text, or Methods section.

n/a	Confirmed	
☐	☒	The exact sample size (*n*) for each experimental group/condition, given as a discrete number and unit of measurement
☐	☒	A statement on whether measurements were taken from distinct samples or whether the same sample was measured repeatedly
☒	☐	The statistical test(s) used AND whether they are one- or two-sided<br>*Only common tests should be described solely by name; describe more complex techniques in the Methods section.*
☒	☐	A description of all covariates tested
☒	☐	A description of any assumptions or corrections, such as tests of normality and adjustment for multiple comparisons
☐	☒	A full description of the statistical parameters including central tendency (e.g. means) or other basic estimates (e.g. regression coefficient) AND variation (e.g. standard deviation) or associated estimates of uncertainty (e.g. confidence intervals)
☒	☐	For null hypothesis testing, the test statistic (e.g. *F*, *t*, *r*) with confidence intervals, effect sizes, degrees of freedom and *P* value noted<br>*Give P values as exact values whenever suitable.*
☒	☐	For Bayesian analysis, information on the choice of priors and Markov chain Monte Carlo settings
☒	☐	For hierarchical and complex designs, identification of the appropriate level for tests and full reporting of outcomes
☒	☐	Estimates of effect sizes (e.g. Cohen's *d*, Pearson's *r*), indicating how they were calculated

Our web collection on statistics for biologists contains articles on many of the points above.

Software and code

Policy information about availability of computer code

Data collection	Carl Zeiss AxioVision software, version 4.6.3 SP1, for acquisition of phase contrast microscopy images<br>MinKNOW software, versions 21.02.1 and 21.11.7, for Nanopore sequencing<br>MiSeq control software, version 2.5.0.5, for read cloud metagenome sequencing
Data analysis	ATLAS commit 59da38f for metatranscriptome read QC<br>ATLAS commit 96e47df (jmtsuji fork) for read mapping of metatranscriptome data to genome bins<br>ATLAS version 2.1.4 for environmental metagenome assembly, binning, and annotation<br>ATLAS version 2.2.0 for enrichment cultures metagenome assembly and genome binning<br>ATLAS version 2.8.2 for short read QC prior to hybrid genome assembly<br>Annotree web server (relying on GTDB release 89) for screening of functional genes in reference genomes<br>ArcGIS version 10.3.1.4959 for geospatial data processing<br>BBMap versions 37.78, 37.99, and 38.75 for sequence read mapping<br>BLAST versions 2.9.0 and 2.10.1 for amino acid searches<br>BWA-MEM version 0.7.17 for short read mapping<br>BackBLAST version 2.0.0-alpha3 for gene ortholog detection<br>BioPython version 1.81 for GC content and GC skew calculations<br>CheckM versions 1.0.7 and 1.0.18 for verifying genome completeness and contamination<br>Circlator version 1.5.5 for repair/rotation of circular genome assemblies<br>Circos version 0.69.8 for genome visualization<br>Clustal Omega versions 1.2.3 and 1.2.4 for multiple sequence alignment<br>ColabFold version 1.5.2-patch for structural prediction of electron transport proteins

CutAdapt version 3.4 for adapter trimming of metagenome data and Nanopore-based amplicon sequencing data
CutAdapt version 4.1 for adapter trimming of Illumina-based amplicon sequencing data
DADA2 versions 1.10.0 and 1.22.0 corresponding to the above QIIME2 versions
DeepTMHMM release 1.0.24 for prediction/classification of transmembrane proteins
Dendroscope version 3.8.3 for (bacterio)chlorophyll synthesis gene phylogeny visualization
FastANI version 1.33 to generate genome clustering information
Flye version 2.9-b1768 for long-read genome assembly
FoldSeek webserver release 8-ef4e960 for protein structure searches
FragGeneScanPlusPlus commits 471fdf7 (LeeBergstrand fork) and 9a203d8 for short open read frame prediction
GToTree version 1.4.11 for concatenated core protein phylogenies
Gblocks version 0.91b for alignment masking
Guppy version 5.0.16 and 5.1.12 for basecalling of Nanopore sequencing data
HMMER (hmmsearch and/or hmmbuild) versions 3.1b2 and 3.3.2 for profile Hidden Markov Model building and searching
I-TASSER web server for protein homology modeling (accessed 2019.5 and 2020.3)
IQ-TREE versions 1.6.9 and 2.2.0.3 for maximum likelihood phylogenies
JalView version 2.11.2.7 for visualizing a RCI gene sequence alignment
Kaiju web server for taxonomic classification of metagenome reads (accessed 2020.2; proGenomes database updated 2017.5.16)
MMseqs2 version 13.45111 for clustering of Nanopore 16S rRNA gene amplicon data following sequence polishing
MaxBin 2 version 2.2.4 for genome binning
Medaka 1.4.4 for polishing of long-read genome assemblies
MetaBAT2 versions 2.12.1 and 2.15 for genome binning
Minimap2 version 2.23 for mapping of long read data
NanoCLUST commit a09991c (jmtsuji fork) for analysis of Nanopore 16S rRNA gene amplicon data
PGAP versions 2022-02-10.build5872 and 2022-04-14.build6021 for genome annotation
POLCA version 4.0.8 for short read polishing of genome assemblies
Picard version 2.21.6 for demultiplexing of read cloud data
Polypolish version 0.5.0 for short read polishing of genome assemblies
Prodigal version 2.6.3 for genome annotation
Prokka version 1.14.6 for genome annotation
QGIS versions 2.14.0 and 3.6.3 for geospatial data visualization
QIIME2 versions 2019.10 and 2022.8 for 16S ribosomal RNA gene amplicon sequence analysis
Rotary commit e636236 and fd5acee for hybrid genome assembly (custom code from this study: https://github.com/rotary-genomics/rotary)
ScanProsite web tool with PROSITE release 2023_04 for motif identification
Summary of custom analysis code used for this manuscript: https://github.com/jmtsuji/Ca-Chlorohelix-allophototropha-RCI
Tell-Read (v0.9.7) and Tell-Link (v1.0.0) for demultiplexing, quality control, and assembly of read cloud metagenome sequencing data
UCHIME2 version 11.0.667 (32 bit) for chimera filtration of Nanopore 16S rRNA gene amplicon data
UCSF ChimeraX version 1.3 for protein structure comparisons
featureCounts 1.6.4 and pandas 1.2.3 for metatranscriptome read count calculations
make-lineage-csv.py from https://github.com/dib-lab/2018-ncbi-lineages, commit 0d41546 for taxonomic lineage mapping
samtools version 1.15 to analyze read mapping data

For manuscripts utilizing custom algorithms or software that are central to the research but not yet described in published literature, software must be made available to editors and reviewers. We strongly encourage code deposition in a community repository (e.g. GitHub). See the Nature Portfolio guidelines for submitting code & software for further information.

Data

Policy information about availability of data

All manuscripts must include a data availability statement. This statement should provide the following information, where applicable:

- Accession codes, unique identifiers, or web links for publicly available datasets
- A description of any restrictions on data availability
- For clinical datasets or third party data, please ensure that the statement adheres to our policy

Enrichment culture metagenomes and MAGs from the L227-S17 culture (subcultures 1 and 15.2) and the L227-5C (primary enrichment) culture are available under NCBI BioProject accession PRJNA640240. Amplicon sequencing data are available at the same BioProject accession. The complete strain L227-S17 genome, along with associated raw read and amplicon sequencing data, are available at BioProject accession PRJNA909349. Similarly, the complete Geothrix sp. L227-G1 genome and associated long read data (subculture 15.c) are available at BioProject accession PRJNA975665. Metagenome data from 2016, sequenced by the JGI, are available in the JGI Genome Portal under Proposal ID 502896. Environmental metagenome and metatranscriptome data from 2017-2018 are available under NCBI BioProject accession PRJNA664486. The full set of 756 metagenome-assembled genomes used for read mapping of metatranscriptome data are available at BioProject accession PRJNA1003647; genome and annotation versions used for read mapping are available in a Zenodo repository (doi:10.5281/zenodo.3930110). The SILVA SSU database (release 132) and the Genome Taxonomy Database (release 89) are available at https://www.arb-silva.de/download/archive/ and https://data.gtdb.ecogenomic.org/releases/, respectively. In addition, the NCBI Protein Reference Sequences (RefSeq) database and 16S rRNA gene database for Bacteria and Archaea type strains are both available at https://ftp.ncbi.nlm.nih.gov/blast/db/; taxonomy mapping information is available at https://ftp.ncbi.nih.gov/pub/taxonomy/.

Research involving human participants, their data, or biological material

Policy information about studies with human participants or human data. See also policy information about sex, gender (identity/presentation), and sexual orientation and race, ethnicity and racism.

Reporting on sex and gender	N/A
Reporting on race, ethnicity, or	N/A

other socially relevant groupings	
Population characteristics	N/A
Recruitment	N/A
Ethics oversight	N/A

Note that full information on the approval of the study protocol must also be provided in the manuscript.

Field-specific reporting

Please select the one below that is the best fit for your research. If you are not sure, read the appropriate sections before making your selection.

☐ Life sciences ☐ Behavioural & social sciences ☒ Ecological, evolutionary & environmental sciences

For a reference copy of the document with all sections, see nature.com/documents/nr-reporting-summary-flat.pdf

Ecological, evolutionary & environmental sciences study design

All studies must disclose on these points even when the disclosure is negative.

Study description	Novel photosynthetic bacteria were cultivated from anoxic lake water. Lake water was distributed into glass bottles, amended with medium, incubated under light, and monitored for microbial growth and activity. Bottles where growth was apparent were fed additional medium and eventually subcultured to enrich high biomass of the target bacterium. Genomic DNA was extracted and sequenced from the enriched bacterial culture.<br><br>The environmental relevance of the cultured bacteria was then examined by surveying nine lake water columns. Water was filtered to collect cell biomass, and DNA or RNA was extracted from filters. This DNA or RNA was prepared into metagenome or metatranscriptome libraries, respectively, and was sequenced using third-generation high-throughput sequencing methods. The resulting data were used to identify relatives of the cultured organisms and to determine their environmental distribution and activity.<br><br>The physiology of the culture was also examined using spectroscopic and microscopy-based methods.
Research sample	Anoxic lake water collected from nine iron-rich Boreal Shield lakes. Water from all nine lakes was used for environmental DNA/RNA analysis. Water from two of the lakes was used for cultivation of photosynthetic bacteria.
Sampling strategy	Water was collected anoxically via line and gear pump from anoxic water columns of the lakes. For enrichment cultures, for each sampled depth, water was collected into duplicate glass serum bottles. For environmental DNA analysis, filters were collected in duplicate for each depth, and for environmental RNA analysis, filters were collected and analyzed in biological triplicate for each depth. Because enrichment cultivation was a qualitative study and the environmental survey was exploratory in nature, sampling size calculations for statistical purposes were not applicable.
Data collection	At the field site, dissolved oxygen and temperature data were collected along the water column via sonde to determine the location of the oxic/anoxic zone boundary of the lake. Data was recorded in a field book and then digitized. A field sampling crew including J.M.T. and others acknowledged in this work collected the data. At the laboratory, geochemical measurements (e.g., of ferrous iron concentrations) for the enrichment culture bottles were taken by J.M.T. via spectrophotometer and were stored digitally. Chemical measurements of lake water samples were performed by others acknowledged in this work and were stored digitally. DNA sequencing data for enrichment cultures was collected digitally by J.M.T. and/or N.A.S. using a MiSeq (Illumina) and/or Nanopore MinION sequencer. Environmental DNA and RNA sequencing data was collected digitally by sequencing centers cited in this work using a HiSeq (Illumina). Physiological measures of the cultured bacterium were recorded digitally via spectrophotometers or image recording software used with microscopy equipment.
Timing and spatial scale	All lake water samples for enrichment cultivation were collected during a single field sampling excursion in September 2017. Samples were collected from the point in the lake where the water column was deepest. Because enrichment cultivation work was qualitative in nature, temporal and spatial replication was not applicable for field sampling. The broader lake survey was performed over four sampling excursions in June 2016, September 2016, September 2017, and July 2018 and included nine lakes. All lakes were sampled from the point where the lake water column was deepest. Most surveyed lakes, including all lakes for which metatranscriptome sequencing data are available, were sampled at least two times to provide temporal replication.
Data exclusions	Although multiple points along the oxic and anoxic portions of each lake water column were sampled, this paper only reports samples where metagenome data are available for the lake anoxic zones. Metatranscriptome data were presented for Lake 227 at the oxic/anoxic zone boundary around the same depth from which enrichment culture samples were obtained. Additional metatranscriptome data across the water column of Lake 227 were not presented in this work because they were not relevant to the research question of this study.<br><br>For physiology work, a light vs. dark cultivation test was performed for the L227-S17 culture under photoautotrophic and photoheterotrophic conditions. Spectroscopy data are only reported in this work from cultures grown photoheterotrophically due to poor quality absorption spectra obtained for the photoautotrophic condition. Data from the photoheterotrophic condition was sufficient to demonstrate phototrophic activity.

Reproducibility	We enriched the novel photosynthetic bacterium in at least two different bottles from the experimental setup. However, the bacterium did not grow in all bottles. For the culture where the bacterium was successfully enriched, we could reproducibly grow the bacterium in subculture. For environmental DNA analysis, nine lakes were surveyed to provide spatial replication across the IISD-ELA sampling site. For environmental RNA analysis, all samples were collected and analyzed in biological triplicate to ensure reproducibility. For physiological experiments, light vs. dark growth tests were performed in biological triplicate.
Randomization	Genomic DNA samples from the recovered cultures were randomized when amplifying marker genes for DNA sequencing. This randomization ensured that samples would not be biased by their physical location on the 96 well plate used for DNA sequencing library preparation. Similarly, genomic DNA from environmental samples was randomized prior to DNA extraction to avoid DNA extraction biases. For RNA extraction, randomization was not performed due to the small number of samples that were extracted.
Blinding	Blinding was not possible for enrichment cultures in this study, because the researcher working with the samples had to be aware of the history of each sample to make informed decisions of how to feed or subculture the sample over the course of laboratory incubation. For metagenomes and metatranscriptomes, technicians were not aware of the biological significance of each sample during library preparation and sequencing. Blinding was not performed during DNA and RNA extractions, although DNA samples were pre-randomized (see above). Blinding was not performed during spectroscopic and microscopy-based studies of culture physiology due to low sample size.

Did the study involve field work? ☒ Yes ☐ No

Field work, collection and transport

Field conditions	Samples were collected in the summer or fall of 2016, 2017, and 2018 during daylight hours. Depending on the sampling excursion, sampling conditions ranged from 0% to 100% cloud cover with no to light rainfall. Upper water temperatures ranged from ~16°C to ~24°C during sampling, and anoxic water column temperatures ranged from ~4°C to ~12°C.
Location	Sampling was performed at the International Institute for Sustainable Development Experimental Lakes Area (near Kenora, Ontario, Canada) on Boreal Shield terrain. The GPS coordinates of the IISD-ELA are 49.50-49.75° N, 93.50-94.00° W. The area is generally 360-380 m above sea level.
Access & import/export	Sampling access was provided by staff at the International Institute for Sustainable Development Experimental Lakes Area ,who have established protocols for responsible sample collection. For example, all garbage or chemical wastes generated on site are collected and exported to a safe transfer facility, and wastewater is also treated. Samples were transported domestically after collection. No sampling or import/export permits were required.
Disturbance	No disturbance of the field site occurred outside of normal operations of the International Institute for Sustainable Development Experimental Lakes Area. No garbage, sampling supplies, or fuels were left at the lake.

Reporting for specific materials, systems and methods

We require information from authors about some types of materials, experimental systems and methods used in many studies. Here, indicate whether each material, system or method listed is relevant to your study. If you are not sure if a list item applies to your research, read the appropriate section before selecting a response.

Materials & experimental systems

n/a	Involved in the study	
☒	☐	Antibodies
☒	☐	Eukaryotic cell lines
☒	☐	Palaeontology and archaeology
☒	☐	Animals and other organisms
☒	☐	Clinical data
☒	☐	Dual use research of concern
☒	☐	Plants

Methods

n/a	Involved in the study	
☒	☐	ChIP-seq
☒	☐	Flow cytometry
☒	☐	MRI-based neuroimaging
