# Supplementary material for: Anoxygenic phototroph of the Chloroflexota uses a type I reaction centre
Source: Nature. 2024 Mar 13;627(8005):915–22. doi: 10.1038/s41586-024-07180-y (PMC10972752; doi:10.1038/s41586-024-07180-y)
Supplement: Supplementary file 3 — Peer Review File [file 41586_2024_7180_MOESM3_ESM.pdf]

Peer Review File

Manuscript Title: Anoxygenic phototroph of the Chloroflexota uses a Type I reaction center

Reviewer Comments & Author Rebuttals

Reviewer Reports on the Initial Version:

Referees' comments:

Referee #1 (Remarks to the Author):

This is an important paper that expands the group of bacterial taxa with phototrophic representatives. As such it is a something of a landmark, in that new groups of phototrophs come along only every 15-20 years. The authors have done an outstanding job of isolating and characterizing the organisms, which are members of the Chloroflexota phylum, but have a type I reaction center instead of a type II found in other phototrophic members, and also has electron transfer and carbon fixation pathways that differ from know Chloroflexota species. I strongly support publication in Nature, although there are some revisions that are needed, which will significantly improve the paper.

I have one significant problem that really needs to be resolved before the paper can be published. The authors do not determine what type of bacteriochlorophyll is present in the organism (other than the BChl c in the chlorosome). I anticipate that it is BChl a, but it could be BChl g or possibly some novel pigment. The paper never addresses this critical issue. It should be relatively easy to determine this using HPLC extraction and spectral analysis. Perhaps some of the minor HPLC peaks in Fig. 1b are these pigments, although the three traces shown have no peaks that are common to all three. It is not reasonable to consider that the only pigment is BChl c, as it is only found in chlorosome antennas, never in reaction centers or other antenna complexes such as FMO. Also, some of the genes essential for BChl a or g biosynthesis (BCHXYZ) are found in the genome, strongly suggesting that there is a longer wavelength pigment present. This is a critical issue that must be resolved.

Another point that needs some additional analysis is the electron transport chain, including the cytochrome bc1/b6f complex versus Alternative Complex III. It is only addressed briefly in Supplementary note 2. It seems that these new bugs are most similar to the Heliobacteria (which makes even more critical the pigment determination).

I question the evolutionary conclusions (lines 65-74 and 305-312). The conclusion that the ancestor contained either a type I or type II or both RC tells you absolutely nothing. Those are the only possibilities. My suggestion is to tone down this sort of talk. The paper stands as a landmark without being weighed down with a lot of speculation.

Referee #2 (Remarks to the Author):

Tsuji and colleagues present a fascinating paper on newly isolated bacteria from the Chloroflexota, which are phototrophic and contain Reaction Center I, as opposed to all other known phototrophs this phylum, which contain Reaction Center II. The paper employs an impressive combination of metagenomics, microbial physiology, electron microscopy, environmental studies and phylogenetics. It uncovers what I think is an extremely significant hint towards the evolution of oxygenic photosynthesis. I have absolutely no doubt that this deserves to be published in Nature, as I think this may be a first step towards a radical re-think about how (and in whom) modern forms of photosynthesis evolved.
I cannot comment on the validity of the metagenomic analyses or the physiological measurements, but as far as my expertise goes, I see nothing worth criticizing in this paper. My only comment is that the branches on the RCI phylogeny are all extremely long, so I would view the exact position of these newly discovered RCIs on that tree as somewhat uncertain, especially with respect to Heliobacteriales RCIs. Long branch attraction can produce very high bootstrap support, so the fact that support values are high on this tree is not strong evidence for the exact position of these proteins. This does not, however, undermine any of the conclusions of this paper.
Instead of a list of demands for revisions (of which I have none), I will take this space to explain why I think this paper is extremely important. The evolution of photosynthesis, in particular oxygenic photosynthesis, is one of the most important and enduring mysteries of evolutionary history. Before the present study, only cyanobacteria were known to harbor both types of reaction centers, in their case as part of oxygenic photosynthesis. The group in which these two reaction centers first came together was unknown, and by some people assumed to probably be a lineage that is now extinct. The present paper now implies that this lineage may in fact be the Chloroflexota. Even though this study does not yet prove that this group really contains both centers in the same genome, it strongly hints that it either did in the past, or that as of yet undiscovered members of it have both types of reaction centers. This discovery seems very significant to me, because other circumstantial evidence is slowly accumulating that this particular group may hold the key to understanding the evolution of oxygenic photosynthesis. Oxygen tolerant Form I Rubiscos, for example, probably first evolved in the Chloroflexota as well (described in work from the Banfield, Shi, and Hochberg groups, if the authors are interested in citing this). Indeed, the present study also discovers deep branching Form I Rubiscos in the same group. Many components of what would be necessary for oxygenic photosynthesis are thus beginning to turn up in this group of microbes.
I expect that when this paper is published and publicized, the race to hunt for more members of the Chloroflexota that further close the gap between anoxic and oxygenic photosynthesis will begin to heat up. As far as I can judge, this paper is a beautiful example of how so far uncultured bacteria contain within their genomes evidence that can help resolve some of the deepest questions in evolutionary biology. I think the work is very comprehensive, beautifully done, and highly impactful. I strongly recommend publication in Nature.

Referee #3 (Remarks to the Author):

Tsuji et al present a great scientific advance in our understanding of the modularity and evolution of photosynthesis, highlighting how classical microbiology culturing techniques can lead to remarkable biological discoveries. The discovery of a deep branching RCI in a member of the Chloroflexota has huge implications on our understanding of the evolution of photosynthesis. This study demonstrates for the first time that some bacterial phyla can have members that contain RCI- or RCII- based phototrophy. This is similar – but importantly distinct – from Cyanobacteria, which (for the most part) have members that have both RCI and RCII in the same cell. All other phototrophic bacterial phyla have only been demonstrated to have a single RC represented in a given phylum, which has long suggested that the metabolism of such microbes has evolved over billions of years to accommodate phototrophy and there may be intrinsic challenges to the swapping of RCs. Overall, these interpretations have also fueled hypotheses on the deep origins of oxygenic photosynthesis, which are widely debated and extremely controversial in the field. Tsuji et al's findings provide an important new organism that will further reinvigorate such debate.

The experiments are beautifully carried out; the results and data are clear and direct. My only concern with this manuscript is the interpretations, and how the authors' interpretations may be further misconstrued by others in the community – especially those that may not have a strong background in phylogenetics and microbial evolution. These concerns can be boiled down to the authors being seemingly partial to the proposed "genetic displacement model". For the first portion of the paper, the authors do a great job tiptoeing the line on presenting the two equally probable scenarios: 1) an ancestral Chloroflexota with a single RC (either RCI or RCII) swapped its original RC for another one (genetic displacement model) or 2) an ancestral Chloroflexota that originally had both RCs and subsequently lost one of them (differential loss model). Later in the paper, the authors seem to more overtly prefer the differential loss model. If the data clearly supported this model, I would not have a problem with this – as it would finally provide some clarity on this widely debated subject; however, the phylogenetic evidence for this is not concrete (Fig 5, see below) and the subsequent interpretations are a bit far-reaching. Importantly, even if the data cannot support either model with clarity, the overall findings have huge implications on the field. Thus, I believe if the authors continue their impartial perspective throughout the entirety of the paper, this paper would have more impact, than proposing a model that is not entirely supported by the data.

There may be those in the field (and possibly even some of the reviewers) that may want to jump to conclusions based on Tsuji et al's findings. I believe this paper is a landmark study; and thus, the interpretations must be equally cautious as it will be a paper that is widely cited and will drive many future hypotheses of the evolution/origins of photosynthesis. For this reason, the paper would be strengthened if the authors were more unbiased in their evolutionary interpretations and let the reader make their own interpretation of the competing models (genetic displacement or differential loss).

Major comments:

The authors' interpretation of "shared genetic ancestry of phototrophy" between RCI- and RCII-utilizing

Chloroflexota (line 274) is not especially strong from Figure 5. Perhaps the strongest instance of this is Fig 5b, which shows how RCI-utilizing Chloroflexota are sister to both RCII-utilizing Chloroflexota and Bacteroidota for the BchlDH tree. However, the ChlLNB tree (Fig 5C) doesn't have strong bootstrap support for this same topology, so I'd be hesitant to argue that this is clear evidence for vertical inheritance of phototrophy. Similarly, in Fig 5d for the BchXYZ tree, I would expect the RCI-utilizing and RCII-utilizing Chloroflexota to cluster more closely to one another to give more concrete evidence that this is a case for vertical inheritance. The fact that the authors are suggesting vertical inheritance of chlorosome and bacteriochlorophyll genes given this non-definitive evidence is in line with a potential bias for the differential loss model, which I believe is premature to propose given the data presented.

I believe the paper would be strengthened with text from the authors discussing the seemingly impressive metabolic plasticity of Chloroflexota that seemingly can rewire their photosynthetic electron transport with entirely different RCs (and Complex IIIs). In either the genetic displacement or the differential loss models, it seems undeniable that Chloroflexi have been able to swap in quite disparate forms of phototrophy. In the genetic displacement model, the possible scenario of swapping out these multisubunit complexes is quite extraordinary. In the differential loss model, this the ability to lose one of these halves is also quite amazing. I feel like this is an overlooked and incredibly important observation that should be bolstered in the "Evolutionary implications" section. This not only has huge implications on our understanding of the evolution of photosynthesis, but also synthetic biology efforts that have long dreamt of engineering phototrophy into organisms. The plasticity of microbial metabolisms to adopt and easily foster new phototrophic machinery has been seen as a "holy grail" that is too complex for us to engineer. These findings suggest that Chloroflexota have a uniquely modular metabolism that is able to interchangeably change their photosynthetic electron transport machinery, which is aligned with recent findings that their metabolism and autotrophic capacity is also equally amenable to metabolic changes/innovations/additions/losses.

Line 309: I don't understand why it is likely that an ancestral Chloroflexota member would encode for both RCI and RCII. This statement makes it seem like the authors are preferential to the differential loss model; however, I don't see any reason why it is likely that RCI and RCII would both be encoded in the ancestor. Either flesh out this statement, or omit it. One of the strengths of this paper, up until this point, is that it seems impartial about the interpretation of their incredible finding.

Line 313: I think it's a bit of a reach to say that Chloroflexota have a parallel history to specifically Cyanobacteria. It has a parallel history to all phototrophic bacterial phyla because they have both RCI-containing and RCII-containing members; however, without finding an extant Chloroflexota member that has both RCI and RCII, it seems a bit premature to start specifically drawing comparisons to oxygenic photosynthesis. It is obvious why the authors may want to draw parallels to Cyanobacteria, but I'd urge the authors to not feel pressured to draw parallels to oxygenic photosynthesis, as this is highly speculative and not really within the scope of their discovery.

Line 317: I don't agree that the authors' findings represent evidence that "RCI and RCII have interacted outside the Cyanobacteria". The finding is that there is a Chloroflexota member that utilizes a deep

branching form of RCI. There is no RCII in this genome, so I don't see how the authors can claim that there is any interaction between RCI and RCII in Ca. Chx. allophototropha. This seems like a statement that could be grossly misinterpreted by the photosynthesis field. I suggest omitting this statement or substantially rewriting and qualifying it.

Minor comments:

Line 328: "bases" should be "basis"

Author Rebuttals to Initial Comments:

Responses to peer review for:

Anoxygenic phototroph of the *Chloroflexota* uses a Type I reaction center

Tsuji JM, Shaw NA, Nagashima S, Venkiteswaran JJ, Schiff SL, Watanabe T, Fukui M, Hanada S, Tank M, Neufeld JD

Summary

We are very thankful for the constructive input of the three scientific reviewers of our submitted manuscript. Recognizing the scientific merit of our work and the impact of our cultivation-based discovery for the field of photosynthesis, the reviewers requested that we include additional details about the pigments and electron transport systems used by "*Candidatus* Chlorohelix allophototropha" strain L227-S17 and that we simplify our discussion about the implications of our findings for the evolution of photosynthesis. In response to these requests, we have performed additional cultivation-based experiments and genomic analysis as discussed below. These additional analyses are supported by two additional figures that, with the permission of the editor, we would like to add to the Supplementary Information of our manuscript. We have also streamlined our evolutionary discussion. We hope that the revised manuscript represents a robust work that highlights the importance of our discovery of a highly novel Type I reaction center-utilizing photosynthetic bacterium in the *Chloroflexota* phylum.

Reviewer-specific comments

Reviewer #1

<u>Topic 1: Identification of bacteriochlorophyll *a* or *g*</u>

In response to Reviewer #1's request for additional pigment data, we identified that bacteriochlorophyll *a* is present in the L227-S17 culture and searched for whether the genome of strain L227-S17 encodes a bacteriochlorophyll *g* synthase. Based on these data, it is likely that bacteriochlorophyll *a* is used as a reaction center-associated pigment in strain L227-S17 cells.

For identification of bacteriochlorophyll *a*, we already had HPLC-based spectral data, prior to our initial submission to Nature, that indicated that this pigment was present in the L227-S17 culture. These data were generated by the first author of this work, JM Tsuji, at Hokkaido University. Despite having these data on hand, we were reluctant to include these data in our initial submission because of

weak signal intensity. After receiving peer review feedback on our manuscript, we attempted new experiments on the culture to obtain data with a higher signal-to-noise ratio. Unfortunately, Tsuji moved to a new research institute (the Japan Agency for Marine-Earth Science and Technology; JAMSTEC) this year and is currently the only researcher on our team who is growing the culture. Establishing high-biomass growth of "*Ca.* Chx. allophototropha" L227-S17 in Tsuji's new laboratory is still incomplete, and two additional HPLC attempts have shown that the cultivation conditions at JAMSTEC are not yet properly optimized (e.g., issues were encountered with lower-than-expected biomass yields or signs of pigment degradation). We are confident, based on preliminary data, that Tsuji will be able to resolve these issues in the coming months, but rather than further delay our current manuscript waiting for new data, we ultimately decided to include our original bacteriochlorophyll *a* measurements (from Hokkaido University) in this revised manuscript version.

To validate detection of bacteriochlorophyll *a* in our original data, we ran a sample of the purple nonsulfur bacterium *Rhodobacter capsulatus* SB1003 as a standard on the same HPLC system used for the original analysis. Because of laboratory environment differences between the old and new HPLC runs (which were performed in the winter and summer, respectively, affecting room temperature and pump/oven performance; Japan is heavily conserving energy usage), we ran a backup of the originally used *Chloroflexus aurantiacus* sample alongside the *R. capsulatus* sample so that we could normalize retention times between the old and new HPLC run data. Combining our original HPLC data with this new bacteriochlorophyll *a* standard allowed us to produce Supplementary Fig. 1, which we include in the Supplementary Information of the revised manuscript (and is included below for reference). This figure gives strong support that the L227-S17 culture included low levels of bacteriochlorophyll *a*, with characteristic *in vitro* absorption peaks at ~365 and ~770 nm (± 2 nm).

For the purpose of peer review, in this response file, we also include intermediate analysis data for the normalization of retention times between the old and new HPLC run data (Fig. R1 below). In short, the new HPLC run data simply needed to be shifted by a retention time of 6.163 min to align with our original data. Methods for identification of bacteriochlorophyll *a* are included in a dedicated sub-section of the Supplementary Methods ("Identification of bacteriochlorophyll *a*", lines 285-298). We comment on the presence of bacteriochlorophyll *a* in the L227-S17 culture in the main manuscript on lines 88-91.

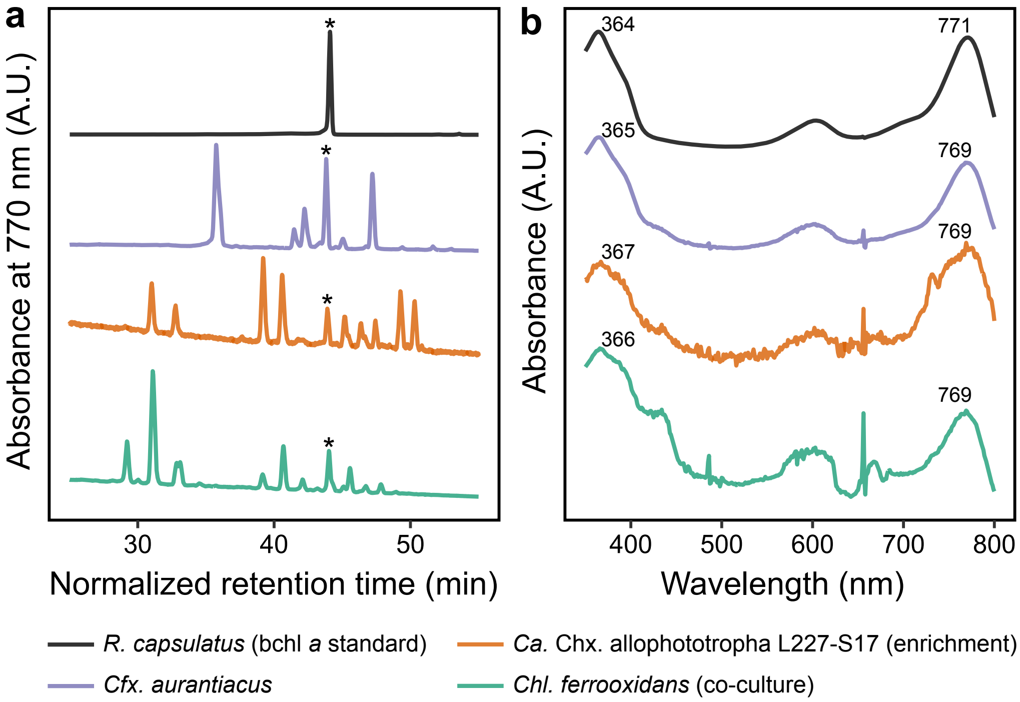

Supplementary Fig. 1 | Identification of bacteriochlorophyll *a* in the L227-S17 culture. a, HPLC profiles of absorbance at 770 nm. The HPLC profiles are the same as shown at an absorbance of 667 nm in Fig. 1b, except a sample of *Rhodobacter capsulatus* was added as a bacteriochlorophyll *a* standard. **b**, *In vivo* absorption spectra associated with HPLC peaks marked with asterisks in **a**. Sharp absorption peaks at 485-486 nm and 655-656 nm are anomalies associated with the HPLC system.

As Reviewer #1 pointed out, several photosynthesis genes used by strain L227-S17 are phylogenetically related to those used by *Heliobacteriales* members, making it important to assess whether bacteriochlorophyll *g* might be produced by the strain. Well-characterized genes involved in bacteriochlorophyll *g* synthesis are homologous to those used in other bacteriochlorophyll synthesis pathways, so detection of gene homologs alone is insufficient to determine whether strain L227-S17 has genomic potential to synthesize bacteriochlorophyll *g*. Because the (bacterio)chlorophyll synthase used by *Heliobacteriales* members for bacteriochlorophyll *g* synthesis is phylogenetically distinct from other (bacterio)chlorophyll synthases, we decided to evaluate the phylogenetic placement of all (bacterio)chlorophyll synthases encoded by strain L227-S17 to assess the possibility for one of these synthases to function in bacteriochlorophyll *g* synthesis. The results of our analysis are presented in Supplementary Figure 2, which we include in this peer review file (below) for convenience.

We found that strain L227-S17 encodes four possible (bacterio)chlorophyll synthase genes. One can be clearly identified as a bacteriochlorophyll *a* synthase gene (*bchG*; shown in Supplementary Fig. 2), and two more cluster phylogenetically with bacteriochlorophyll *c* synthase (*bchK*; omitted from Supplementary Fig. 2 for clarity). The fourth gene was described in our original submission as a

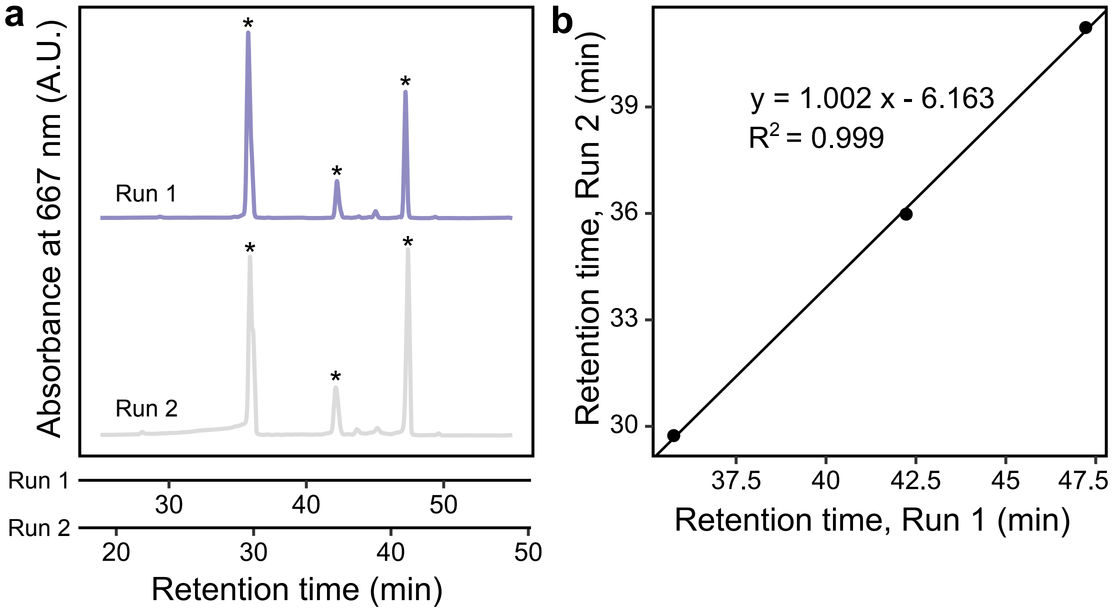

Reviewer Response Fig. R1. Normalization of HPLC run retention time between original and new HPLC data using a sample of *Chloroflexus aurantiacus* cells. Replicate tubes of the same *Chloroflexus aurantiacus* sample were used for two HPLC runs: the HPLC run used for bacteriochlorophyll *c* analysis in our originally submitted manuscript (Run 1) and a new HPLC run containing a bacteriochlorophyll *a* standard (Run 2). **a**, Comparison of bacteriochlorophyll *c* species between the two samples based on absorbance at 667 nm. The Run 1 data is identical to that shown in Fig. 1b-c of the main manuscript. Retention times of the two runs are shown along the x-axis. **b**, Linear regression comparing the retention times of three highest peaks from panel **a** (marked with asterisks) between Run 1 and Run 2. Because the slope of the regression was ~1, we could simply add 6.163 min to the retention times of the new HPLC run (Run 2) to normalize the retention times. The detected bacteriochlorophyll *a* peak was identified at a Run 1 retention time of ~44 min (Fig. S1), meaning that this peak is within the retention time range of the linear regression.

possible chlorophyll *a* synthase (*chlG*). Phylogenetic analysis in Supplementary Fig. 2 supports that this fourth gene could indeed represent a chlorophyll *a* synthase (*chlG*), but the gene is deep-branching compared to known *chlG* genes and is sister to the *Heliobacteriales* bacteriochlorophyll *g* synthase clade, meaning we cannot rule out that the gene could function in bacteriochlorophyll *g* synthesis. Other characterized RCI-utilizing phototrophs, such as *Chlorobaculum tepidum*, use bacterio-chlorophyll *a* and chlorophyll *a* in their reaction centers, but a microorganism using both bacterio-chlorophyll *a* and *g* for phototrophy has never been reported. We thus consider it more likely that the gene represents a deep-branching chlorophyll *a* synthase but leave open either possibility for the role of this gene in our manuscript. Overall, we feel that our evidence of usage of bacteriochlorophyll *a* by the culture is sufficient for the primary goal of this paper, that is, to demonstrate active RCI-based

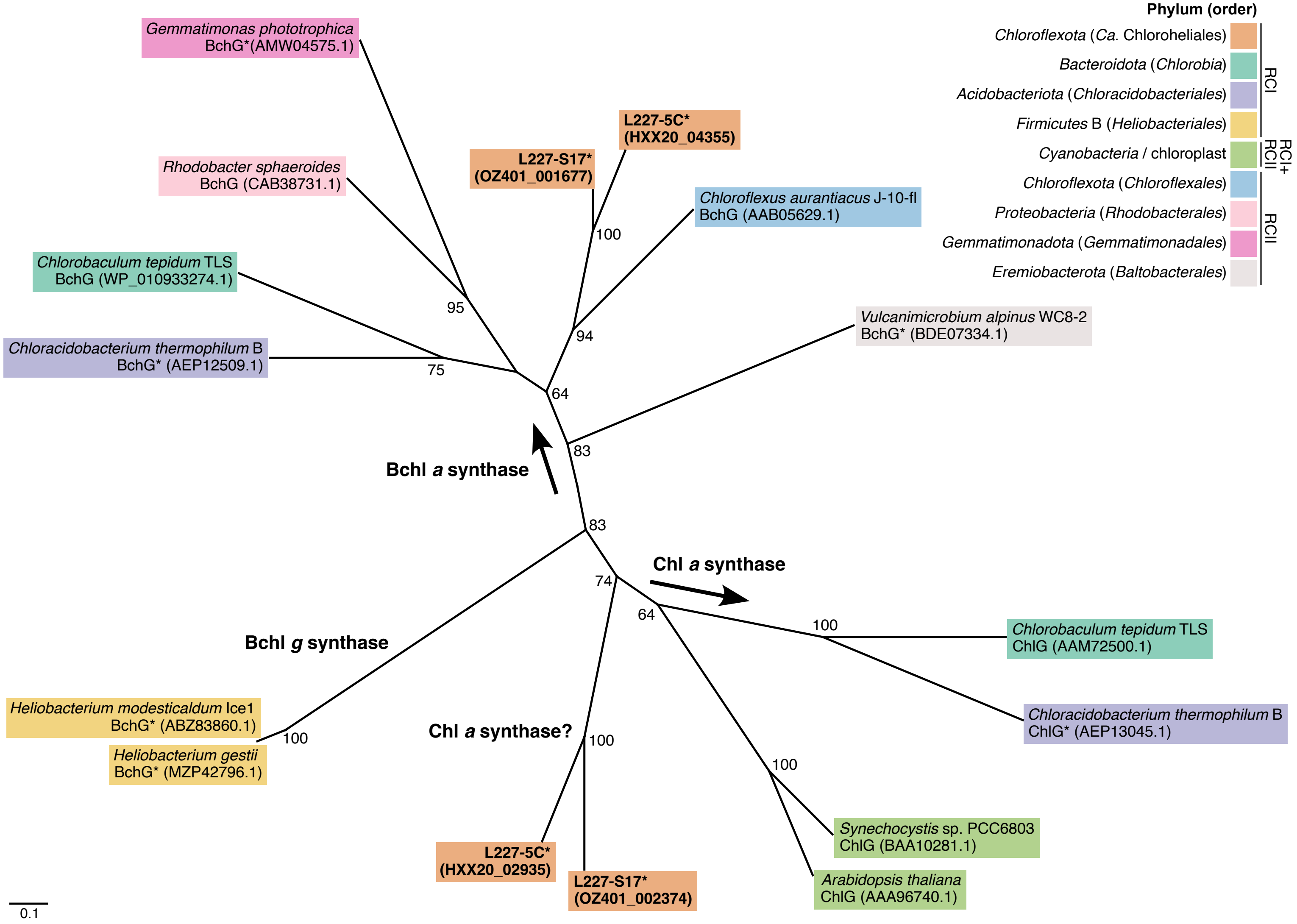

Supplementary Fig. 2 | Possible chlorophyll *a* synthase encoded by strain L227-S17. The maximum likelihood sequence phylogeny shows (bacterio)chlorophyll synthase genes associated with synthesis of chlorophyll *a*, bacteriochlorophyll *a*, and bacteriochlorophyll *g* from representatives of known phototroph-containing phyla. Sequence names followed by an asterisk indicate that the sequence was identified by homology rather than based on a direct reference in the literature. Bootstrap values over 50% are shown. The scale bar represents the expected proportion of amino acid change.

phototrophy by strain L227-S17. Future experimental work could examine more closely the minor pigments associated with the novel RCI complex of this strain.

In the main text, we mention the fourth (bacterio)chlorophyll synthase as a possible *chlG* homolog that also bears some similarity to heliobacterial bateriochlorophyll *g* synthases (lines 160-162). Methods for performing our phylogenetic analysis of the (bacterio)chlorophyll synthases of strain L227-S17 are included in the Supplementary Methods in lines 547-559.

Topic 2: Characterizing the possible electron transport system of "*Ca.* Chx. allophototropha" L227-S17

We appreciate the suggestion to more thoroughly characterize the electron transport system of the novel phototroph using genome data. We performed a rigorous search for homologs of genes involved in photosynthetic electron transport in strain L227-S17 using genes of other RCI-utilizing phototrophs and RCII-utilizing *Chloroflexota* members as references. Many proteins involved in photosynthetic electron transport, such as the direct electron donor protein to RCI, are not conserved between different phototroph lineages (e.g., due to convergent evolution), and other proteins (e.g., heme-binding proteins) have short primary sequences and are difficult to identify accurately. As such, most of our electron transport gene hits do not have the same level of confidence as other well-defined photosynthesis marker genes, such as (bacterio)chlorophyll synthase genes and RCI genes. Although we were unable to detect any clear electron transport gene homologs in the L227-S17 genome, compared to known phototrophs, for some genes (e.g., genes encoding the direct electron donor protein to RCI), we did detect a possible homolog of a gene encoding the terminal electron acceptor protein of RCI, referred to as *pscB* in *Chlorobiales* members. We also identified multiple ferredoxin genes in the L227-S17 genome that could be involved in subsequent electron transfer after PscB.

To increase our confidence in our electron transport gene findings, we mapped genes of "*Ca.* Chx. allophototropha" L227-S17 to genes of "*Ca.* Chlorohelix bin ELA319", which was recovered from Boreal Shield lake metagenome data, and we then checked whether candidate genes in bin ELA319 were highly expressed in Boreal Shield lake metatranscriptome data. Genes for both the PscB candidate and one of the identified ferredoxins were upregulated in bin ELA319, which adds support that these genes could be of functional importance for phototrophy. Although we could not detect a strong candidate gene for Ferredoxin—NADP+ reductase (FNR), involved in linking phototrophy to carbon fixation, we identified an alternative FAD-dependent oxidoreductase gene that is adjacent to the *pscB*-like gene of strain L227-S17; the equivalent gene in bin ELA319 was highly expressed. This gene could be of interest as a convergently evolved FNR, given that *Chlorobiales* members are already known to use a distinct form of FNR compared to other phototrophs. Altogether, this analysis provides

some support to our conclusion that "*Ca.* Chx. allophototropha" L227-S17 is a RCI-based phototroph but also highlights the need for subsequent biochemical study to confirm our findings and identify unknown proteins in the electron transport chain.

In addition to genes involved in photosynthetic electron transport, we also searched for and identified other electron transport gene systems in strain L227-S17. We identified two gene clusters for Complex I, like have been identified for *Chloroflexus aurantiacus*, implying that "*Ca.* Chx. allophototropha" L227-S17 might possess metabolic flexibility like has been observed for other RCII-utilizing *Chloroflexota* members. Future cultivation-based work will be needed to verify these predictions.

We briefly comment on our findings from this analysis in the main text on lines 150-157. In addition, we provide a long-form explanation of our findings, including locus tags of identified genes, in Supplementary Note 2 within the Supplementary Information (lines 91-181). We also added a panel to Extended Data Fig. 4 (panel b), with description on lines 132-136 in the main text, to show that the novel PscA-like protein sequence contains the conserved residues needed to bind the F_X iron-sulfur center required for photosynthetic electron transfer. Methods for our analyses are described in the Supplementary Methods on lines 525-546.

<u>Topic 3: Evolutionary comments</u>

As suggested by Reviewer #1, we remove mention of the use of RCI vs. RCII vs. both RC's (by the most recent common ancestor of phototrophic *Chloroflexia* members) from the manuscript. We agree that the wording of this phrase was confusing and distracted from the purpose of the work. In general, we have reduced our evolutionary comments in the revised manuscript so that the focus of the work rests more on our cultivation-based and ecological findings. Our specific changes are summarized in response to Reviewer #3 below.

In summary, we thank Reviewer #1 for their constructive feedback on our work. We agree with the value of each of Reviewer #1's requests and think that the revised manuscript is stronger from having integrated Reviewer #1's suggestions.

Reviewer #2

We appreciate Reviewer #2's enthusiasm about this work and strongly agree with the reviewer's comment that this work "may be a first step towards a radical re-think about how (and in whom) modern forms of photosynthesis evolved". In response to Reviewer #2's suggestions, we removed our

comment about relative branch placements in the RCI phylogeny (Fig. 2), given that these branch placements may not be reliable. As suggested, we also incorporated references to recent works by the Banfield, Shi, and Hochberg groups that suggest that oxygen-tolerant Form I RuBisCOs may have originated in the *Chloroflexota* phylum (lines 275-277 of the main text). Altogether, we greatly appreciate Reviewer #2's feedback about the value of our findings and their potential implications that could drive future work.

Reviewer #3

We thank Reviewer #3 for their carefully thought-out comments about the evolutionary implications of our work. We agree that a distinction needs to be made between the core findings of our manuscript (i.e., usage of RCI by a *Chloroflexota* member) and the implications of those findings. We also agree that presentation of our findings in a way that does not appear biased is key for this paper to be robust and helpful for long-term scientific progress.

In response to Reviewer #3's feedback, we made several major changes to the Introduction and Discussion our of manuscript. In summary, these changes implement suggestions by Reviewer #3 in the following ways:

- We simplify the thesis of our manuscript, in the final paragraph of the introduction (lines 56-61), to focus more on our core cultivation-based discovery and less on evolutionary implications,
- We remove our reference to possible parallels between the evolution of the *Chloroflexota* phylum and the *Cyanobacteria* phylum, given that this statement is ambiguous, and replace it with a better-defined statement that future study of the evolutionary history of the *Chloroflexota* phylum could yield insights into the evolution of oxygenic photosynthesis (lines 272-274, also incorporating feedback from Reviewer #2),
- We greatly reduce the total text length describing the evolutionary implications of our work,
- We simplify our description of the two possible evolutionary models that could explain the diversification of phototrophic *Chloroflexota* members (lines 248-257), and we only briefly comment on the implications of either model,
- We intentionally avoid biased wording in the subsequent text following the description of the two possible evolutionary models,
- We remove our claim that an ancestral *Chloroflexota* member likely encoded both RCI and RCII at some point during the history of the phylum in either model,

- We omit our statement that "RCI and RCII have interacted outside the *Cyanobacteria*" – we agree that this statement stretched beyond the scope of our work and could be misleading – and
- We create a new "Discussion" subsection at the end of the manuscript (lines 260-285) to more broadly discuss the implications of our work for photosynthesis research, including comments about the metabolic flexibility of *Chloroflexota* members (lines 267-272).

Although evolutionary implications are still a central component of this work, given that the significance of our discovery is made clearer via comparison to previously known RCII-utilizing *Chloroflexota* members, we feel that the key focus of the work is now more directly on the discovery and physiology of "*Ca.* Chx. allophototropha" L227-S17, compared to the implications of our findings. We hope that this matches the intentions of Reviewer #3's feedback that "the experiments are beautifully carried out; the results and data are clear and direct, [but] my only concern… is the interpretations, and how the authors' interpretations may be further misconstrued by others in the community – especially those that may not have a strong background in phylogenetics and microbial evolution". By focusing more on the direct findings of our work in the updated manuscript version, we hope to better distinguish our core results from how those results could affect broader theory. We leave for future research to explore the implications of our discovery on the biochemistry, physiology, and evolution of photosynthesis.

Responding to other comments by Reviewer #3:

- Bootstrap values in Fig. 5c (ChlLNB phylogeny): we wonder if Reviewer #3 mis-interpreted the bootstrap labels in this figure panel. We omitted any bootstrap labels that were 100/100 to de-clutter the figure, but indeed, this is not typical for bootstrap value presentations (usually low-value bootstraps are omitted, not high-value bootstraps). The node that Reviewer #3 was concerned about actually had 100/100 bootstrap support. To avoid confusion, we have updated Figure 5 so that 100/100 bootstrap labels are shown. Given that the bootstrap support was 100/100, we feel that our claim remains robust that the most likely and unbiased explanation of our phylogenomic data is that the most recent common phototrophic ancestor of RCI- and RCII-utilizing phototrophs in the *Chloroflexia* class was itself phototrophic. As such, we leave this claim in the updated manuscript (in lines 235-247), although we only briefly explore the implications of this idea in the updated work.

- “Bases” vs. “basis”: we have corrected this word in the revised manuscript (line 272).

Taken together, we are thankful for Review #3’s feedback and agree that “even if the data cannot support either [proposed evolutionary] model with clarity, the overall findings have huge implications on the field”. We are excited to see how this work is used by the scientific community to further our understanding of photosynthesis in the coming years.

Additional revisions

Other than revisions for clarity, reduction of text length, and fixing of minor errors or inconsistencies, the following additional areas of the manuscript were edited during our revision process:

- The grammar of the manuscript title was simplified.
- A full species description of “*Ca.* Chx. allophototropha” L227-S17 was added at the end of the main text to meet the requirements of publishing a new bacterial species name (even if Candidatus).
- Additional text labels were added to Fig. 5 so that the clades in the phylogeny are discernable if colour information are not available.
- Gene/genome information in Extended Data Table 2 was updated and clarified.
- GPS coordinates for the nine surveyed Boreal Shield lakes were added to Extended Data Table 3.
- All sequencing data associated with this work were publicly released, and accession numbers were updated in the Data Availability Statement.
- The order and format of the manuscript file was adjusted to match Nature’s requested format.
- Researchers who provided us with Bchl *a* standards were added to the Acknowledgments, along with anonymous peer reviewers who provided critical feedback to the manuscript text.

Reviewer Reports on the First Revision:

Referees' comments:

Referee #1 (Remarks to the Author):

I think the authors have done an excellent job of responding to the questions that I and the other reviewers posed. I support the publication of this important paper in Nature.

Referee #2 (Remarks to the Author):

This was already a great paper in its original form and I have no further comments. I'd like to congratulate the authors to a wonderful piece of work.

Referee #3 (Remarks to the Author):

The authors have addressed all of my comments. I apologize for misinterpreting Figure 5 previously. As the authors guessed, I did not realize that unlabelled nodes signified 100/100 bootstrap values.
